# p16^INK4A^ flow cytometry of exfoliated cervical cells: Its role in quantitative pathology and clinical diagnosis of squamous intraepithelial lesions

**DOI:** 10.1002/ctm2.1209

**Published:** 2023-03-07

**Authors:** Yifeng He, Jun Shi, Hui Zhao, Yuefei Wang, Chi Zhang, Sai Han, Qizhi He, Xiaolan Li, Shangji Li, Wenjing Wang, Muhua Yi, Xiaoling Hu, Zhihua Xing, Hao Han, Yinshuang Gao, Qing Zhou, Linlin Lu, Jianfen Guo, Hui Cao, Caiping Lu, Yanqiang Hou, Dan Chen, Fengyun Yang, Ping Lei, Wen Di, Ji Qian, Yi Xia, Youzhong Zhang, Yang Deng, Jianlong Zhu, Congjian Xu

**Affiliations:** ^1^ Department of Obstetrics and Gynecology Ren Ji Hospital School of Medicine Shanghai Jiao Tong University Shanghai China; ^2^ Shanghai Key Laboratory of Gynecologic Oncology Ren Ji Hospital School of Medicine Shanghai Jiao Tong University Shanghai China; ^3^ Department of Obstetrics and Gynecology Pudong Hospital Fudan University Shanghai China; ^4^ Department of Gynecology Taiyuan Maternal and Child Health Hospital Taiyuan Shanxi China; ^5^ Department of Gynecology Obstetrics and Gynecology Hospital Fudan University Shanghai China; ^6^ Shanghai Key Laboratory of Female Reproductive Endocrine Related Diseases Shanghai China; ^7^ Department of Obstetrics and Gynecology Qilu Hospital Shandong University Jinan Shandong China; ^8^ Department of Pathology First Maternity and Infant Health Hospital Tongji University Shanghai China; ^9^ Department of Obstetrics and Gynecology The Second People's Hospital, Three Gorges University Yichang Hubei China; ^10^ Department of Pathology Dongguan Hospital Southern Medical University Dongguan Guangdong China; ^11^ Department of Obstetrics and Gynecology Yongcheng People's Hospital Yongcheng Henan China; ^12^ Department of Obstetrics and Gynecology Zouping People's Hospital Zouping Shandong China; ^13^ Department of Pathology The Central Hospital of Zibo Mining Group Co. Ltd. Zibo Shandong China; ^14^ Department of Obstetrics and Gynecology The Central Hospital of Zibo Mining Group Co. Ltd. Zibo Shandong China; ^15^ Department of Obstetrics and Gynecology Chifeng College Affiliated Hospital Chifeng Inner Mongolia China; ^16^ Department of Clinical Laboratory Songjiang Hospital, School of Medicine, Shanghai Jiao Tong University Shanghai China; ^17^ Fosun Diagnostic Technology (Shanghai) Co., Ltd Shanghai China; ^18^ Department of Cervical Diseases Jiading Maternal and Child Health Care Hospital Shanghai China; ^19^ Department of Gynecology Zhuhai Center for Maternal and Child Health Care Zhuhai Guangdong China; ^20^ State Key Laboratory of Oncogene and Related Genes Shanghai Cancer Institute Ren Ji Hospital, School of Medicine, Shanghai Jiao Tong University Shanghai China; ^21^ State Key Laboratory of Genetic Engineering Institute of Genetics, School of Life Sciences, Fudan University Shanghai China

**Keywords:** cervical cancer, flow cytometry, human papillomavirus, immunostaining, p16^INK4A^, papanicolaou smear, pathology, quantification, squamous intraepithelial lesion

## Abstract

**Background:**

P16^INK4A^ is a surrogate signature compensating for the specificity and/or sensitivity deficiencies of the human papillomavirus (HPV) DNA and Papanicolaou smear (Pap) co‐test for detecting high‐grade cervical squamous intraepithelial lesions or worse (HSIL+). However, traditional p16INK4A immunostaining is labour intensive and skill demanding, and subjective biases cannot be avoided. Herein, we created a high‐throughput, quantitative diagnostic device, p16INK4A flow cytometry (FCM) and assessed its performances in cervical cancer screening and prevention.

**Methods:**

P16^INK4A^ FCM was built upon a novel antibody clone and a series of positive and negative (p16^INK4A^‐knockout) standards. Since 2018, 24 100‐women (HPV‐positive/‐negative, Pap‐normal/‐abnormal) have been enrolled nationwide for two‐tier validation work. In cross‐sectional studies, age‐ and viral genotype‐dependent expression of p16^INK4A^ was investigated, and optimal diagnostic parameter cut‐offs (using colposcopy and biopsy as a gold standard) were obtained. In cohort studies, the 2‐year prognostic values of p16^INK4A^ were investigated with other risk factors by multivariate regression analyses in three cervicopathological conditions: HPV‐positive Pap‐normal, Pap‐abnormal biopsy‐negative and biopsy‐confirmed LSIL.

**Results:**

P16^INK4A^ FCM detected a minimal ratio of 0.01% positive cells. The p16^INK4A^‐positive ratio was 13.9 ± 1.8% among HPV‐negative NILM women and peaked at the ages of 40–49 years; after HPV infection, the ratio increased to 15.1 ± 1.6%, varying with the carcinogenesis of the viral genotype. Further increments were found in women with neoplastic lesions (HPV‐negative: 17.7 ± 5.0–21.4 ± 7.2%; HPV‐positive: 18.0 ± 5.2–20.0 ± 9.9%). Extremely low expression of p16^INK4A^ was observed in women with HSILs. As the HPV‐combined double‐cut‐off‐ratio criterion was adopted, a Youden's index of 0.78 was obtained, which was significantly higher than that (0.72) of the HPV and Pap co‐test. The p16^INK4A^‐abnormal situation was an independent HSIL+ risk factor for 2‐year outcomes in all three cervicopathological conditions investigated (hazard ratios: 4.3–7.2).

**Conclusions:**

FCM‐based p16^INK4A^ quantification offers a better choice for conveniently and precisely monitoring the occurrence of HSIL+ and directing risk‐stratification‐based interventions.

## Graphical abstract

• The quantification detection system—p16^INK4A^ FCM was established for triaging cervical high‐grade neoplasia/cancer.

• 24 100‐women cross‐sectional studies revealed that HSIL+‐triaging efficacy of HPV and p16^INK4A^ FCM co‐test is significantly higher than that of HPV and Pap co‐test.

• 2‐year cohort studies indicated that HPV‐positive/Pap‐abnormal p16^INK4A^‐abnormal women would run a HSIL+‐risk > 4‐fold higher than those with a p16^INK4A^‐normal level.

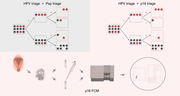

## 1 BACKGROUND

Cervical lesions caused by human papillomavirus (HPV) undergo a series of molecular events to eventually develop into invasive cancers; through this process, the affected cells acquire unrestricted proliferation and metastasis features.^1^, ^2^, ^3^ The remarkable events, in order of occurrence, include the expression of viral E6 and E7 oncoproteins, defunction and degradation of the host tumour suppressors p53 and RB, disinhibited expression of a minor tumour suppressor – p16^INK4A^, and finally, reactivated expression of Ki‐67 and hTERT, which are two necessary components of genome DNA replication.^4^, ^5^, ^6^ Current cervical cancer‐monitoring techniques, such as the HPV DNA test and Papanicolaou smear (Pap), only inform clinicians of the onset of HPV infection and the cytological outcome of infection‐induced molecular events,^7^ while the latter, that is, the Pap test, which provides a cytopathological diagnosis, is currently categorised by the Bethesda System (TBS) terms, namely, negative for intraepithelial lesion or malignancy (NILM), atypical squamous cells of undetermined significance/cannot exclude a high‐grade lesion (ASC‐US/ASC‐H), low‐grade/high‐grade squamous intraepithelial lesion (LSIL/HSIL), squamous cell carcinoma (SCC) and so on.^8^ To date, there is a lack of tools to probe HPV‐induced intracellular molecular alterations before the formation of pathomorphological lesions. Nevertheless, p16^INK4A^ overexpression has been confirmed to be a key event in cervical carcinogenesis and is a unique signature for estimating viral E6‐ and E7‐induced p53 and RB defunctions.^4^, ^9^, ^10^ However, although the p16^INK4A^ mono‐ or p16^INK4A^/Ki‐67 dual‐immunostaining techniques have been widely applied by pathologists to recognise pre‐cancerous/cancerous lesions and have achieved significant success in cervical pathology,^11^, ^12^, ^13^, ^14^, ^15^, ^16^ detection tools for intimately monitoring p16 ^INK4A^ dysregulation events as well as for real‐time assessment of cervical pre‐cancer/cancer risks are not yet available.

The flow cytometric (FCM) technique offers a high‐throughput, quantitative and automatic platform for determining the absolute number (or percentage) of abnormal cells.^17^ This technique has been successfully used in the immunophenotyping and counting of a specific group/histotype of peripheral blood cells (e.g., leukaemia).^18^, ^19^ However, unlike the cytotype‐specific expression pattern of clusters of differentiation (CD proteins) in blood cells,^19^, ^20^ the viral E6‐/E7‐induced dysregulated expression of p16^INK4A^ (e.g., overexpression) continuously changes from extremely low levels to excessively high levels, which spans a wide profile of pathological conditions, namely, normal, inflammatory, pre‐cancerous (e.g., ASC, LSIL, ASC‐H and HSIL) and cancerous (e.g., invasive cancer) conditions.^10^ Despite an increasing overexpression trend of p16^INK4A^ in the high‐grade lesion and invasive cancer conditions, there are no substantial gaps or nicks visible in the cell number versus p16^INK4A^‐signal intensity two‐dimensional curve, which might be used as a signature to divide abnormal/neoplastic cells from normal cells during FCM analysis^21^; conversely, for other histotypes of cells, such as leukaemia cells, these gaps can be easily observed and used to discriminate neoplastic cells from non‐neoplastic cells, thereby dividing them into two independent groups.^19^, ^20^ Moreover, due to a scarce number (1/10 000–10/10 000) of neoplastic (pre‐cancer/cancer) cells, true p16^INK4A^‐positive cells can be submerged by a number of mistakenly labelled pseudo/autofluorescent cells, and this comprises a major source of background noise in FCM.^21^ Other reasons for false‐positive signals include unstable photon‐electron transformation during the FCM detection process and/or physical‐physiological variations/changes in the collected cells, such as extreme cell sizes and cell‐to‐cell adhesions.^21^, ^22^, ^23^

In recent years, we have sought to construct a reliable detection system to quantitatively profile the p16^INK4A^ expression status in cervical epithelial cells. Based on the p16^INK4A^ quantification system developed, which was modified from a traditional FCM platform, most random measurement errors, such as those caused by the optoelectronic fluctuations of FCM signal‐processing or biophysical heterogeneity of the exfoliated cells, were satisfactorily controlled by a parallelly prepared reference sample (blank‐labelled); additionally, the systemic errors caused during sample‐harvesting/labelling processes and/or FCM read in/readout procedures were rectified by a set of external standards (a calibrating ladder). With these refinements, the utility of FCM in detecting abnormal cells of extremely low abundance (1/10 000–10/10 000) that were labelled with a difficult‐to‐measure dose of fluorescence signals was enabled. Herein, we report the diagnostic performance of this novel system. The physiological and pathological expression patterns of p16^INK4A^ in normal/abnormal cervical cells were quantitatively investigated. The age‐, HPV genotype‐ and lesion‐dependent expression of p16^INK4A^ was precisely appraised, and the use of the p16^INK4A^‐positive ratio for assessing the risks of high‐grade lesions was validated. The obtained FCM data provided important evidence for the applicability of p16^INK4A^‐based quantitative pathology in the risk stratification‐oriented management of women threatened with neoplastic lesions.^16^, ^24^, ^25^

In general, the current study aimed to address the critical questions that might be encountered during clinical application of p16^INK4A^ FCM, namely, (1) why was the ‘p16^INK4A^‐positive ratio’ selected as a more sensitive measurement scale for p16^INK4A^ FCM; (2) what is the normal expression pattern of p16^INK4A^ at the cervical epithelium; (3) what changes of cervical p16^INK4A^ expression pattern could be detected by FCM after HPV infection; (4) how is the diagnostic performance of p16^INK4A^ FCM in identifying histological HSIL+ lesions; (5) could the ‘p16^INK4A^‐positive ratio’ be applied to predict the 2‐year pre‐cancer/cancer risks of women with abnormal HPV/Pap tests, especially under complex pathological conditions.

## 2 METHODS

### 2.1 Study population

The study population comprised women who attended an annual gynaecological examination (including a cervical cancer‐screening program) between 20 September 2018 and 20 March 2020, at 10 local medical centres of mainland China, namely, (1) Ren Ji Hospital, School of Medicine, Shanghai Jiao Tong University, Shanghai, (2) Pudong Hospital, Fudan University, Shanghai, (3) Songjiang Hospital (Songjiang Central Hospital), Songjiang, Shanghai, (4) Qilu Hospital, Shandong University, Jinan, Shandong, (5) Zouping People's Hospital, Zouping, Shandong, (6) The Central Hospital of Zibo Mining Group Co. Ltd., Zibo, Shandong, (7) Chifeng College Affiliated Hospital, Chifeng, Inner Mongolia, (8) The Second People's Hospital, Three Gorges University, Yichang, Hubei, (9) Yongcheng People's Hospital, Yongcheng, Henan and (10) Taiyuan Maternal and Child Health Care Hospital, Taiyuan, Shanxi. Women were excluded if they were pregnant, a virgin (or <16 years old), had undergone hysterectomy or had been treated for cervical intraepithelial neoplasia (CIN) or any malignant diseases during the past 5 years. Women with severe acute or chronic mental/infectious/internal/surgical diseases as well as those with autoimmune/posttransplant diseases who relied on daily immunosuppressive drugs or those with endocrinological syndromes/abnormal uterine bleeding who had to use hormone medicines long term were also excluded. The enrolment process contained three phases (Figure S1). For phase 1, 20 September 2018 to 20 March 2019, according to their HPV DNA and Pap testing results, HPV‐negative Pap‐normal women were consecutively enrolled from participating medical centres; signed informed consent was collected; clinical information was recorded; and cervical exfoliate samples (the remnant collection of Pap) were stored for later application of p16^INK4A^ FCM. At each medical centre, eligible HPV‐positive and/or Pap‐abnormal women who attended during the same period were systematically enrolled as a candidate pool, and the information and cervical samples of these women were properly kept until phase 2 or 3. During phase 2, after the age compositions of the enrolled HPV‐negative cervical healthy women were determined, age‐matched HPV‐positive women who had normal Pap results were selected from the candidate pool and called back to participate in our observational study; their informed consent was obtained before the first round of follow‐up. In addition, the candidate pool was continuously expanded by collecting eligible HPV‐positive and/or Pap‐abnormal women during the same phase. Phase 2 enrolment ceased on 20 September 2019, and the study quickly entered phase 3 as the HPV genotyping process had been completed for each of the phase 2 women (i.e., the age‐matched HPV‐positive Pap‐normal women). In phase 3, HPV‐positive/‐negative Pap‐abnormal women were selected from the pool based on the following rules: (1) the enrolled HPV‐negative Pap‐abnormal women should comprise a group with the same/similar age composition as that of the group of HPV‐negative Pap‐normal women; (2) the enrolled HPV‐positive Pap‐abnormal women should comprise a group with the same/similar age composition and HPV genotype composition as those of the group of HPV‐positive Pap‐normal women; (3) the women were randomly selected and called to determine their willingness to participate if they met the age and HPV genotype requirements; informed consent was obtained from the enrolled women before the first round of follow‐up; and (4) if there were still vacancies regarding a specific age and/or HPV genotype condition that could not be fulfilled by the pool, the candidates were selected and enrolled directly from outpatient departments of the participating centres before 20 March 2020. Upon the enrolment deadline, the remnant and redundant candidates in the pool were released, and their information and cervical samples were deleted or discarded under surveillance. The study protocol (Figure S2) was approved by the ethics committee of Ren Ji Hospital and approved by the participating medical centres.

### 2.2 Antibodies and cell lines

The human p16^INK4A^‐specific monoclonal antibodies used for FCM purposes were prepared from a set of novel hybridomas (clones 1H1087, 1A72 and 1B517). There were no clones for FCM purposes commercially available to us before this work (even at the time of publication). To build anti‐human p16^INK4A^ antibody hybridomas, the CDKN2A cDNA sequence (NM 000077.4) was synthesised and inserted into the prokaryotic expression vector pET‐28a, which was then transferred into *Escherichia coli* strain BL21 to produce the full‐length p16^INK4A^ antigen. The *E. coli*‐expressed product was purified, concentrated and applied to immunise BALB/c mice. The obtained novel hybridoma clones were sequentially tested for their immune reactivity and specificity against human p16^INK4A^ protein by ELISA and Western blotting (Figure S3). HeLa cells (ATCC, Manassas, VA, USA) were used as a p16^INK4A^‐positive standard in Western blotting as well as in FCM and were cultured in Dulbecco's modified Eagle medium supplemented with 15% foetal bovine serum in 5% CO_2_ at 37°C. The cell strain used as the p16^INK4A^‐negative standard was constructed by knocking the CDKN2A gene out of HeLa cells, which was implemented through the CRISPR‒Cas9 technique.^26^ The obtained Δ(CDKN2A) HeLa strain was named HeLa16.

### 2.3 Western blotting

Whole‐cell protein extracts of HeLa and HeLa16 cells were separated in 10% SDS‐PAGE gels and transferred to polyvinylidene fluoride (PVDF) membranes (GE Healthcare, Piscataway, NJ, USA). The protein bands were first immunoreacted with our novel p16^INK4A^‐specific monoclonal antibodies (1:500, clones 1H1087, 1A72 and 1B517), an immunochemistry‐purposed mouse anti‐human p16^INK4A^ monoclonal antibody (1:500, clone E6H4; Roche, Basel, Switzerland) or an anti‐human β‐actin antibody (1:500; CW Biotechnology, Beijing, China). The immunoreactions were then detected with a (secondary) HRP‐conjugated rabbit anti‐mouse IgG polyclonal antibody (1:500; Abcam, Cambridge, MA, USA) and visualised with ECL Western Blotting Detection reagents (GE Healthcare).

### 2.4 p16^INK4A^ FCM

For p16^INK4A^ FCM, each of the cell suspensions (prepared from HeLa/HeLa16 cultures or cervical cytological samples) was divided into two equal parts; one part (i.e., the test portion) was sequentially immunoreacted with one of the three novel p16^INK4A^‐specific (primary) monoclonal antibodies (1:100, clones 1H1087, 1A72 and 1B517) and a (secondary) FITC‐conjugated rabbit anti‐mouse IgG monoclonal antibody (1:250; Abcam) in pH 7.4 phosphate‐buffered saline (PBS) at room temperature for 0.5–2 h; and the other (i.e., the reference portion) was immunoreacted with the secondary FITC‐conjugated rabbit anti‐mouse IgG antibody in PBS at room temperature for 0.5 h. A NovoCyte 2060 flow cytometer (Agilent, Santa Clara, CA, USA) was used for testing, and a total of 10 000 events were recorded for each run of the sample. The p16^INK4A^‐positive ratio was defined as the ratio of cells in the test portion of the sample with FITC signals stronger than a reference cut‐off. The reference cut‐off was defined as an FITC signal intensity equal to the lower limit of signal intensity of the top 10, 1 or 0.1% autofluorescent/falsely labelled cells. The FITC signal intensity data of each run were exported into Excel tables (Microsoft, Redmond, WA, USA) to calculate p16^INK4A^‐positive ratios at a given reference cut‐off. The cellular mixtures with HeLa/(HeLa+HeLa16) ratios of 0 (i.e., the negative standard), 1, 5, 25, 50 and 100% (i.e., the positive standard) were used as a ladder that was tested in parallel to calibrate the p16^INK4A^ FCM result in each run (Figure S4).

### 2.5 HPV DNA testing and genotyping

The details of the HPV DNA and genotyping tests have been described previously^27^ with a few modifications. Briefly, for each woman, the total DNA was isolated from a 5 mL liquid‐based cervical cytological sample (collected by cervical brushing) using a QIAamp DNA Mini Kit (Qiagen, Shenzhen, Guangdong, China) and maintained in PBS at −20°C. The quality of the isolated DNA was tested by measuring the β‐actin copies. The consensus HPV L1 primer pair MY09/11 was adopted to amplify viral genomic DNA via the polymerase chain reaction (PCR).^28^ The resultant PCR products were used to construct a DNA fragment library and subjected to next‐generation sequencing on a NovaSeq6000 platform (Illumina, San Diego, CA, USA). The sequencing results were aligned with full‐length L1 DNA information of 27 known HPVs (including HPV‐6, ‐11, ‐16, ‐18, ‐31, ‐33, ‐35, ‐39, ‐42, ‐43, ‐44, ‐45, ‐51, ‐52, ‐53, ‐54, ‐55, ‐56, ‐58, ‐59, ‐60, ‐66, ‐67, ‐68, ‐73, ‐81 and ‐82) to determine the viral genotype(s). If the DNA sequencing result of an HPV‐positive woman did not match any known HPV genotypes or matched a genotype other than the 27 HPVs, the woman was excluded from the study population (or the candidate pool).

### 2.6 Cervical cytology

Cervical cytological samples were collected in ThinPrep fixative (Hologic, Marlborough, MA, USA) for the liquid‐based Pap test.^29^ ThinPrep slides were prepared, stained and processed by using the ThinPrep 2000 System (Hologic). According to TBS 2001, the diagnostic terms on Pap smears were classified into categories as follows: NILM, ASC‐US, LSIL, ASC‐H, HSIL and SCC. All Pap slides were independently analysed by two pathologists. Discrepant diagnoses were reviewed for consensus. The remnant cervical sample of each woman after the preparation of a liquid‐based Pap slide was further used for p16^INK4A^ FCM (10 mL) and HPV DNA testing (5 mL), that is, approximately 15 mL in total.

### 2.7 Follow‐up

HPV‐positive and/or Pap‐abnormal women were followed up regularly at a 3‐month interval for 2 years. Women with HPV infections or cytologically diagnosed with ASC‐US or worse lesions (i.e., ≥ASC‐US) were referred to colposcopy before initiating a regular follow‐up schedule. If their biopsy results indicated an intraepithelial squamous lesion lower than HSIL (e.g., cervicitis, LSIL), the women were then involved in one of the three follow‐up cohorts, namely, HPV‐positive Pap‐normal, Pap‐abnormal biopsy‐negative and biopsy‐confirmed LSIL, based on their initial HPV, cytological and biopsy diagnoses. During follow‐up, the women were offered regular HPV DNA and Pap tests. If their regular Pap results revealed a persistent LSIL lesion over 1 year or a lesion worse than their initial cytological/biopsy diagnoses, the women were referred to colposcopy again. For women whose HPV DNA test revealed evidence of an infection with a novel viral genotype(s) or a relapse of former viral infection following an intermittent negative viral DNA test, colposcopy and biopsy (if necessary) were also referred. The (primary) endpoint event was biopsy‐confirmed HSIL or invasive cervical cancer within 2 years of follow‐up.

### 2.8 Statistics

Two‐sided *χ*2 test/Fisher's exact test and ANOVA/Student's *t*‐test were used to compare enumeration (e.g., age composition, viral genotype composition) and measurement (e.g., p16^INK4A^‐positive ratio, p16^INK4A^ increment) data, respectively. The linear relationship between the actual and FCM‐detected nominal p16^INK4A^‐positive ratios as well as that between the viral genotype‐specific p16^INK4A^ increments and multiple‐infection‐induced variations of genotype‐specific p16^INK4A^‐positive ratios were analysed using Pearson's product‐moment correlation coefficient. The changes in p16^INK4A^ increments between two paired sets of groups of women, for example, HPV‐negative versus HPV‐positive women (age‐group pairs), single infections versus multiple infections (viral genotype pairs) and Pap‐normal versus Pap‐abnormal women (age‐group pairs), were analysed using the Wilcoxon signed‐rank test. The cumulative risk of high‐grade lesions in p16^INK4A^‐normal versus ‐abnormal women was compared using Kaplan–Meier curves and the log‐rank test. The independencies of the hazards (presented as hazard ratios [HRs]) of clinical‐pathological risk factors (e.g., age, viral genotype, Pap test, p16^INK4A^ expression status) contributing to the occurrence of high‐grade lesions during follow‐up were analysed using a univariate or multivariate Cox regression model. The receiver operating characteristic curve was used to determine the optimal cut‐offs of the p16^INK4A^‐positive ratio in diagnosing p16^INK4A^‐abnormal cases among p16^INK4A^ expression higher‐than‐normal (i.e., higher than the average level of p16^INK4A^ expression) and lower‐than‐normal populations. SPSS 18.0 software (IBM, Armonk, NY, USA) was used for analyses, and *p* < .05 was considered statistically significant.

## 3 RESULTS

### 3.1 The p16^INK4A^ FCM quantification system detected p16^INK4A^‐positive cells at a threshold as low as 0.1%

**FIGURE 1:**
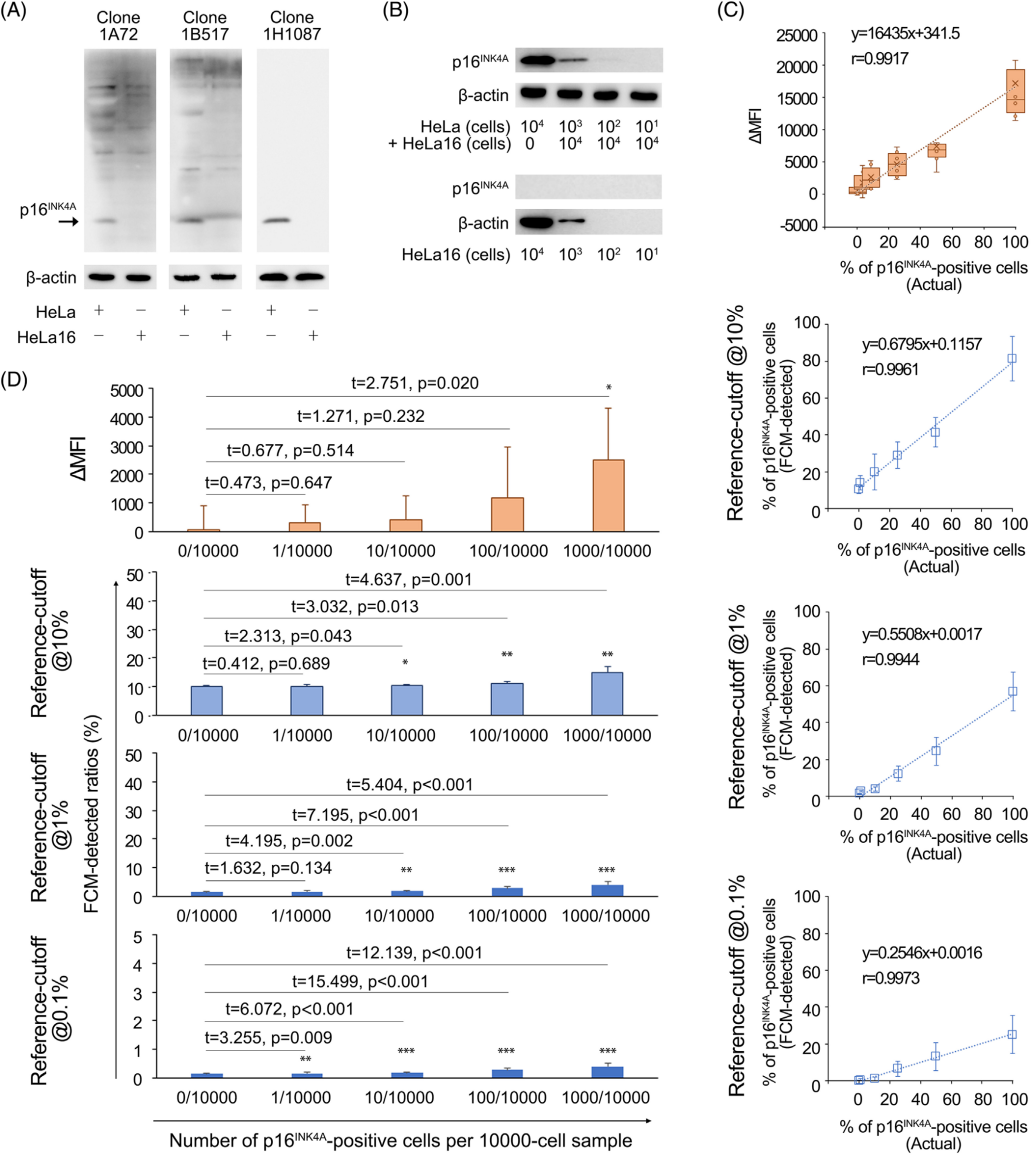
Establishment and evaluation of the p16^INK4A^ FCM detection system. (A) Examination of the immunospecificities of novel antibody clones against human p16^INK4A^ protein by Western blotting. HeLa and HeLa16 cells were used as p16^INK4A^‐positive and p16^INK4A^‐negative protein sources, respectively. Whole‐cell protein extracts were reacted seriatim with one of the three newly prepared monoclonal antibodies, while only clone 1H1087 displayed a clear immunoblotting background, suggesting its high specificity against p16^INK4A^ and low reactivity to non‐p16^INK4A^ cellular proteins. (B) Determination of the detection sensitivity of clone 1H1087 to the p16^INK4A^ proteins at different HeLa/HeLa16 ratios (upper panel) and examination of the thoroughness of the CRISPR‐based p16^INK4A^‐knockout effect in HeLa16 cells (lower panel) by Western blotting. The minimal detectable level of p16^INK4A^ protein by clone 1H1087 was equal to 10^2^ HeLa cells per 10^4^ HeLa16 cells (i.e., HeLa/HeLa16 ratio = 1%), and no p16^INK4A^‐specific bands were observed on the PVDF membrane as up to 10^4^ HeLa16 cells/lane‐equivalent cellular proteins were loaded. (C) The linear relationships between actual percentages of p16^INK4A^‐positive cells and FCM measurements in multiple measuring scales (e.g., ΔMFI and p16^INK4A^‐positive ratios at reference‐cut‐off = 10, 1 and 0.1%, respectively). The tested samples were composed of HeLa/Hela16 mixtures, within which the overall number of cells was fixed at 10.^4^ The actual percentage of the tested sample was deliberately tuned based on the HeLa/(HeLa+HeLa16) ratio. The linear regression functions and coefficients were given for each pair of measurement parameters compared. (D) The minimal detection thresholds of p16^INK4A^ FCM according to the measuring scales. Each FCM measurement for a given percentage of p16^INK4A^‐positive cells (mimicked using a HeLa/Hela16 mixture) was compared with that of a HeLa/(HeLa+HeLa16) ratio = 0 (p16^INK4A^‐negative) sample. The experiments were performed in triplicate, and a two‐sided Student's *t*‐test was used for comparison. The minimal detection threshold conferred by a measuring scale was determined by the minimal number of p16^INK4A^‐positive cells that could be detected by FCM to give a significantly different measurement (compared with a p16^INK4A^‐negative sample) using the indicated scale. The overall number of cells was fixed at 10^4^ for each sample tested (i.e., HeLa/HeLa16 mixture). *, *p* < .05; **, *p* < .01; ***, *p* < .001.

Clone 1H1087, a mouse anti‐human p16^INK4A^ monoclonal antibody obtained by immunising the animal with *E. coli*‐expressed full‐length p16^INK4A^ protein, was selected as a candidate clone to detect p16^INK4A^‐positive cells in the modified FCM platform. The p16^INK4A^ protein‐binding ability (specificity and sensitivity) of this antibody was examined by Western blotting. For whole‐cell protein extracts of HeLa at a loading amount equal to 104 cells/lane (loading volume: 20 μL), clone 1H1087 showed no nonspecific bands on the PVDF membrane, while the other two clones, 1A72 and 1B517, which were prepared in parallel, detected bands for irrelevant proteins in the lysates of HeLa or HeLa16 cells (note, HeLa16 is a p16^INK4A^‐knockout strain of HeLa; Figures 1A and S3). The minimal detectable amount of p16^INK4A^ by clone 1H1087 was equivalent to a protein extract of 10^2^ HeLa cells/lane in Western blotting (Figure 1B). The FCM performance of this clone was further examined with a set of external standards, where HeLa cells were serially diluted with HeLa16 cells. Linear regression analysis indicated that the number of p16^INK4A^‐positive cells (presented as the ‘p16^INK4A^‐positive ratio’ in a 10000‐cell sample) detected by p16^INK4A^ FCM exhibited a perfect linear relationship with the actual numbers (presented as the ‘actual p16^INK4A^‐positive ratio’ in Figure 1C) as reference cut‐offs were set at 10, 1 and 0.1%, respectively (note, ‘FCM‐detectable/FCM‐detected cells’ indicates cells with FITC signal intensities greater than the top 10, 1 and 0.1% of pseudo/autofluorescent cells within a 10 000‐cell sample); a similar linear relationship was found between the increments of mean fluorescence intensity (ΔMFIs) measured and the actual numbers of p16^INK4A^‐positive cells for a series of external standards (Figure 1C). The regression coefficient *r* values between the FCM‐detected ratios of p16^INK4A^‐positive (reference‐cut‐off = 10%), strong‐positive (reference‐cut‐off = 1%) and extremely strong‐positive (reference‐cut‐off = 0.1%) cells and their actual numbers (percentages) reached 0.9961, 0.9944 and 0.9973, respectively, and were highly close to the performance (*r* = 0.9917) of the ΔMFIs (Figure 1C). Regarding the minimal detection threshold (or detection resolution) of the clone 1H1087‐based FCM, we found that a slight variation of 0.1% or even 0.01% of positive cells (i.e., 1–10 cells) within a 10 000‐cell sample was sensitively detected as the reference cut‐off was set to 1 or 0.1% (Figure 1D and Table S1). However, the ΔMFI measurement system could only detect a variation in the positive ratio > 10% (i.e., alteration of the number of positive cells > 1000 cells per 10 000‐cell sample). Therefore, compared with ΔMFI, the ‘p16^INK4A^‐positive ratio’ is a more sensitive measurement scale (Figure 1D). Since the minimal detectable variation in the p16^INK4A^‐positive ratio (i.e., highest resolution) using FCM reached 0.1% at the reference cut‐off of 10% (i.e., 10 cells per 10000‐cell sample; Figure 1D and Table S1), which satisfied the requirement for the establishment of an effective cervical cancer screening program, we then applied the reference cut‐off of 10% as a routine FCM setting for testing clinical samples in the next tasks in our study.

### 3.2 FCM‐detected p16^INK4A^‐positive cells displayed an age‐dependent distributional mode in the normal cervix

**FIGURE 2:**
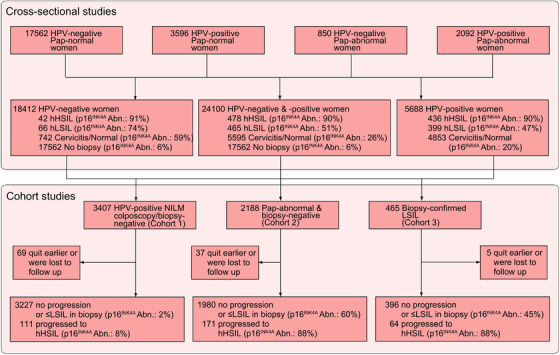
The research profile of two‐tier clinical validation work for p16^INK4A^ FCM. We performed two tiers of clinical investigations on the diagnostic and prognostic efficacies of p16^INK4A^ FCM. First, 24 100 women with different HPV infection statuses and Pap testing results were enrolled from ten medical centres nationwide. Cross‐sectional diagnostic studies were conducted and the diagnostic efficacies of p16^INK4A^ FCM on histological HSIL+ were determined by comparing its performances with a gold standard technique: colposcopy‐guided biopsy. Shown were the p16^INK4A^ FCM detection abnormal rates in women with specific histopathological categories as confirmed by biopsy. The optimal detection criterion was obtained by calculating the maximal Youden's index under various cut‐off‐ratios of p16^INK4A^ FCM. Second, the prognostic efficacies were validated in three cervicopathological conditions, namely, HPV‐positive Pap‐normal, Pap‐abnormal biopsy‐negative and biopsy‐confirmed LSIL. The cohorts for performing the corresponding 2‐year prospective observational studies were collected from the 24 100 women in initial cross‐sectional diagnostic studies. Women, who have completed colposcopy examinations with biopsy pathological results meeting the criteria, were subenrolled into the corresponding follow‐up cohort. hLSIL, histological LSIL. hHSIL, histological HSIL. p16^INK4A^ Abn., the percentage of cases with abnormal p16^INK4A^ FCM detection results within a specific women population.

**FIGURE 3:**
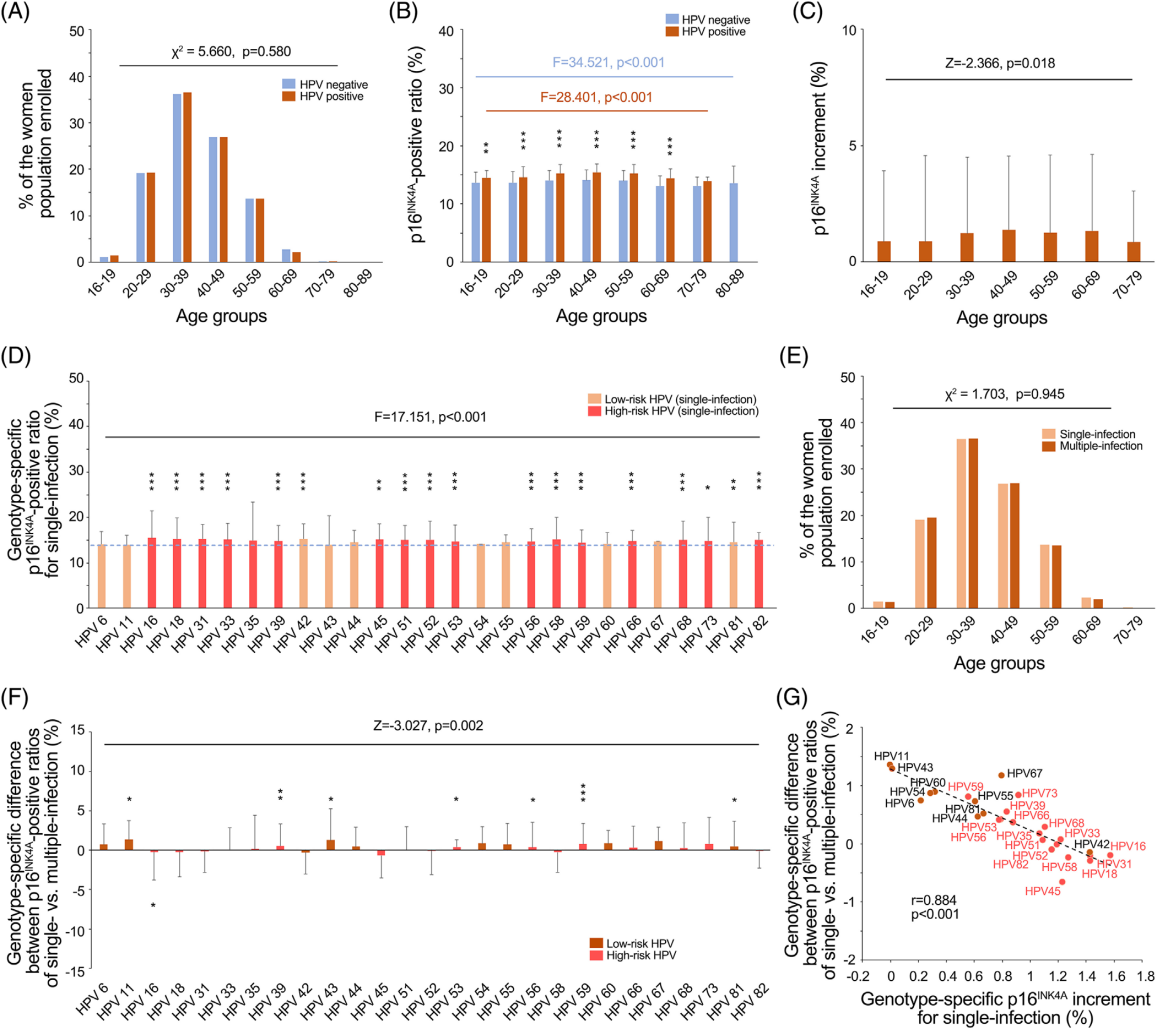
The age‐ and viral genotype‐dependent expression of p16^INK4A^ in the Pap‐normal condition. (A) The age compositions of the HPV‐negative and HPV‐positive NILM women, which were compared using the two‐sided *χ*2 test. (B) The age‐dependent expression of p16^INK4A^ in the HPV‐negative and HPV‐positive NILM women, which was analysed by ANOVA among the respective populations. For each age group, the expression levels (positive ratios) of p16^INK4A^ were compared between HPV‐negative and HPV‐positive women using the two‐sided Student's *t*‐test. (C) The p16^INK4A^ increments of each age group, which were compared using the Wilcoxon signed‐rank test. (D) The viral genotype‐dependent expression pattern of p16^INK4A^ in HPV‐positive NILM women, which was analysed using ANOVA. The expression level of p16^INK4A^ (i.e., p16^INK4A^‐positive ratio) in women infected with a specific viral genotype was seriatim compared with the average p16^INK4A^ level of HPV‐negative NILM women using the two‐sided Student's *t*‐test. The dotted line signifies the average level of p16^INK4A^ in an HPV‐negative NILM population. (E) The age compositions of the women with single and multiple infections in the HPV‐positive NILM population, which were compared using the two‐sided *χ*2 test. (F) The viral genotype‐specific p16^INK4A^ increments of multiple infections relative to their respective single‐infection counterparts. The overall difference in p16^INK4A^ increments between single and multiple infections was compared using the Wilcoxon signed‐rank test. The intragenotypic p16^INK4A^ increment difference was compared using the two‐sided Student's *t*‐test between cases with single and multiple infections. (G) The relationship between the genotype‐specific p16^INK4A^ increments of single infections relative to the average p16^INK4A^ level of HPV‐negative NILM women and the genotype‐specific p16^INK4A^ increments of multiple infections relative to the corresponding single‐infection counterparts was analysed using Pearson's product‐moment correlation coefficient (the dotted line). The linear regression coefficient *r* and *p* value are given. HR HPVs are highlighted in red characters. *, *p* < .05; **, *p* < .01; ***, *p* < .001.

We consecutively enrolled 17562 women (Figures 2 and 3A, also Figure S2) who visited the outpatient department for an annual gynaecological examination to explore the age‐dependent p16INK4A expression pattern in the normal cervix (see Table S2 for the demographic, clinical and pathological characteristics of the enrolled women). All these women were negative for cervical HPV DNA and Pap tests. At the reference cut‐off of 10%, the detected p16^INK4A^‐positive ratio was highest in women aged 40–49 years (i.e., 14.1 ± 1.7%), while this ratio dropped in women aged 60 years (Figure 3B and Table S6). The actual number of p16^INK4A^‐positive cells decreased to approximately 70% of the peak in women aged ≥60 (4.1 vs. 3.1 per 100 sample cells; note, the FCM‐detected positive ratio was 13.1 ± 1.7% for women in the age group of 60–69 years; Table S6). Considering the age‐related variation of p16^INK4A^‐positive ratios was prominent and non‐negligible, we thereafter adopted age‐adjusted criteria to assess p16^INK4A^ overexpression in exfoliated cervical cells.

### 3.3 Aberrant expression of p16^INK4A^ in HPV‐infected cervical epithelial cells

Women with a positive HPV DNA test and a normal Pap (see Table S2 for the demographic, clinical and pathological characteristics of the enrolled women) were enrolled to explore the effect of HPV infection on p16^INK4A^ expression (i.e., p16^INK4A^‐positive ratio) in the cervix. We applied an age‐ and HPV genotype‐matching strategy to build study cohorts (i.e., age groups; Tables 1 and 2), which ensured that the genotypic composition of HPV was not significantly different between each cohort and thereby avoided genotype‐related biases in the collected data (Table S3). In the six age groups enrolled (3596 women in total; see Figure 2), HPV‐induced increments of p16^INK4A^‐positive ratios (i.e., p16^INK4A^ increment) varied with the age of the women (Figures 3A–C and Table S3). The detected p16^INK4A^ increment among the HPV‐infected women aged 16–19 years was <1% (0.87%, exactly; Table S6), which was slightly higher (*p* = .001) than that among their HPV‐negative counterparts (Figures 3B and C); however, for women aged 30–69 years, this value increased to >1% (1.22%; Figure 3C) and reached a maximal level of 1.38% in the age group of 40–49 years (Figure 3C), reflecting substantial harm to the host genome after a long‐term HPV infection. Since our age‐ and genotype‐matching strategy also ensured that the populations infected with various viral genotypes shared the same age composition (Tables 2 and S3), we further investigated the age‐independent but viral genotype‐specific p16^INK4A^ increments in the enrolled women. Compared with the p16^INK4A^‐positive ratios detected in the HPV‐negative controls (13.9 ± 1.8%; Table S6), women infected with HPV‐16 (single infections) were associated with the highest p16^INK4A^ increments (1.6%; Figure 3D and Table 2), while those infected with HPV‐18 or other high‐risk (HR) genotypes (single infections) exhibited relatively lower increments (0.5–1.4%; Figure 3D and Tables 2, also Table S3a). For women infected with low‐risk (LR) HPVs, the detected increment was further reduced (−0.002 to 1.4%; Figure 3D and Tables 2, also Table S3a). The differences among genotype‐specific increments were statistically significant (Figure 3F and Table 2, also Tables S3 and S6). Notably, the p16^INK4A^‐positive ratios of HR genotype‐related multiple infections were generally lower than those of their single‐infection counterparts (Figures 3E and F). Although most HR single infections were associated with higher p16^INK4A^ increments (Table 2 and Figure 3D), these increments were significantly reduced as women were co‐infected with LR genotypes (Table 2 and Figure 3F), where the inhibitory effect was proportional to the increment‐inducive effect of the original HR genotype (Figure 3G). In contrast, LR HPVs evoked a lower increment in single‐infection statuses but gained significantly higher promotions in the p16^INK4A^ increment after co‐infection with HR genotypes (Figures 3D, F and G).

**TABLE 1:** The age compositions of HPV‐negative and HPV‐positive women (Pap‐normal) enrolled in this study.

Study populations	16–19^a^ *n* (%)	20–29 *n* (%)	30–39 *n* (%)	40–49 *n* (%)	50–59 *n* (%)	60–69 *n* (%)	70–79 *n* (%)	80–89 *n* (%)	*χ* ^2^	*p* Value^b^
Population of HPV‐negative women (*n* = 17 562)	203 (1)	3373 (19)	6351 (36)	4727 (27)	2396 (14)	475 (3)	35 (0)	2 (0)	5.660	.580
Population of HPV‐positive women (*n* = 3596)	52 (1)	692 (19)	1312 (36)	966 (27)	489 (14)	79 (2)	6 (0)	0 (0)		
Population of women with single‐infections (*n* = 2205)	32 (1)	420 (19)	804 (36)	592 (27)	301 (14)	51 (2)	5 (0)	0 (0)	1.703	.945
Population of women with multiple‐infections (*n* = 1391)	20 (1)	272 (20)	508 (37)	374 (27)	188 (14)	28 (2)	1 (0)	0 (0)		
Equivalent population of single‐infections (*n* = 2205)^c^	32 (1)	420 (19)	804 (36)	592 (27)	301 (14)	51 (2)	5 (0)	0 (0)	7.291	.295
Equivalent population of multiple‐infections (*n* = 3357)	45 (1)	672 (20)	1296 (39)	860 (26)	413 (12)	68 (2)	3 (0)	0 (0)		

^a^ For each age group, the data are presented as number (%). The percentages were calculated based on the number of women enrolled in the indicated study population for each line.

^b^ The two‐sided *χ* ^2^ test was adopted for comparing the differences in age compositions between the HPV‐negative versus HPV‐positive women, women with single infections versus multiple infections and person‐times of single infections versus multiple infections.

^c^ The equivalent population of single or multiple infections took each infection event as one infection person‐time. Additionally, each viral genotype contained in a multiple infection was seen as an independent infection event. Thus, the person‐time number of an age group was equal to the total number of viral genotypes that each woman of this age group was infected with.

**TABLE 2:** The age‐ and viral genotype‐compositions of HPV‐positive Pap‐normal women (single‐infections) and their corresponding p16^INK4A^ increments.

HPV genotypes	16–19^a^ *n* (%)	20–29 *n* (%)	30–39 *n* (%)	40–49 *n* (%)	50–59 *n* (%)	60–69 *n* (%)	70–79 *n* (%)	*χ* ^2^	*p* Value^b^	p16^INK4A^ increment (%)^c^
HPV‐16 (*n* = 489)	8 (2)	100 (20)	163 (33)	132 (27)	71 (15)	13 (3)	2 (0)	3.725	.811	1.6 ± 2.0
HPV‐18 (*n* = 172)	3 (2)	28 (16)	61 (35)	48 (28)	27 (16)	4 (2)	1 (1)	3.215	.864	1.4 ± 1.6
HR HPVs (*n* = 1386)	19 (1)	262 (19)	527 (38)	365 (26)	183 (13)	28 (2)	2 (0)	4.760	.689	1.1 ± 1.5
LR HPVs (*n* = 158)	2 (1)	30 (19)	53 (34)	47 (30)	20 (13)	6 (4)	0 (0)	1.916	.964	0.7 ± 1.5

^a^ For each age group, the data are presented as number (%). The percentage data were calculated based on the number of women infected with a specific genotype of HPV for each line and given in the form of integers in the parentheses.

^b^ For the viral genotypes (for simplicity, presented as HPV‐16, ‐18, HR HPVs and LR HPVs), the age‐related distributional characteristics of the infected women were compared seriatim with that of the HPV‐negative women. The two‐sided *χ*2 test was used for the analysis. Additionally, for the age groups, the viral genotype‐related distributional characteristics of the infected women were also compared with one another using the two‐sided *χ*2 test. All the differences compared in this table were of no statistical significance; details of the statistical analyses can be found in Table S3.

^c^ Data are presented as the mean ± SD. The viral genotype‐related p16^INK4A^ increment was calculated based on the FCM data of those with single infections. The p16^INK4A^‐positive ratios of the women infected with HPV‐16, ‐18, HR HPVs and LR HPVs were seriatim compared with that of the women with no viral infections using the two‐sided Student's *t*‐test (see Figure 3D). The genotype‐specific p16^INK4A^ increments and their related standard deviations (SD) were given. The differences of p16^INK4A^ increments among various HPV genotypes were of statistical significance (*F* = 18.793, *p* < .001, ANOVA). .

### 3.4 Aberrant expression of p16^INK4A^ in pathomorphologically abnormal cervical epithelial cells

**FIGURE 4:**
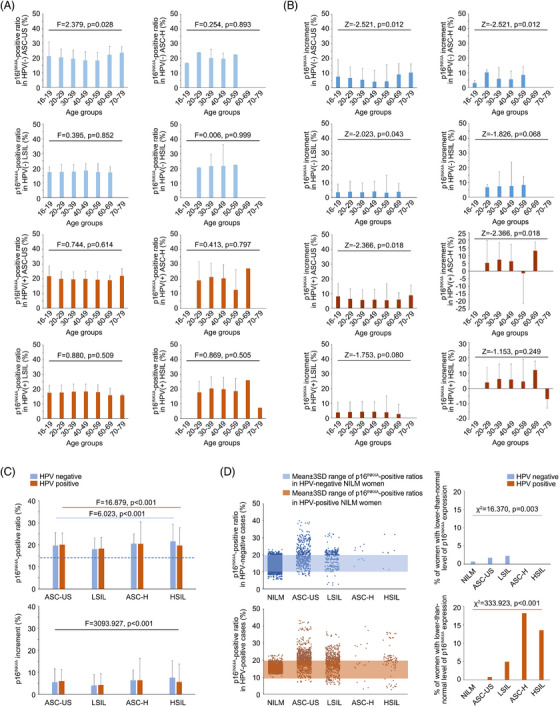
The age‐ and lesion‐related expression patterns of p16^INK4A^ in the Pap‐abnormal condition. (A) The age‐dependent expression of p16^INK4A^ in HPV‐negative and HPV‐positive Pap‐abnormal women. The age‐dependent p16^INK4A^‐positive ratios were separately displayed as per the HPV DNA and Pap test results and were analysed using ANOVA. (B) The age‐dependent patterns of p16^INK4A^ increments per the HPV DNA and Pap test results. For each dataset, the Wilcoxon signed‐rank test was used for statistical analysis. (C) The lesion‐related (classified based on TBS terms) expression patterns (positive ratios and increments) of p16^INK4A^, which were compared using ANOVA. Blue bar, the statistical analysis was performed only for HPV‐negative women. Brown bar, the statistical analysis was performed only for HPV‐positive women. Black bar, the statistical analysis was performed for all Pap‐abnormal women. The dotted line, the average p16^INK4A^ level (positive ratio) of the HPV‐negative NILM population. (D) The lesion‐related distribution patterns of p16^INK4A^‐positive ratios under different HPV DNA and Pap‐abnormal situations. The two‐sided *χ*2 test was performed to analyse the distribution patterns in the HPV‐negative and HPV‐positive conditions. Notably, in the HPV‐negative condition, there were more p16^INK4A^ lower‐than‐normal events found in women with low‐grade lesions, while in the HPV‐positive condition, there were more p16^INK4A^ lower‐than‐normal events found in women with high‐grade lesions; both distribution patterns were of statistical significance. SD, standard deviation.

**FIGURE 5:**
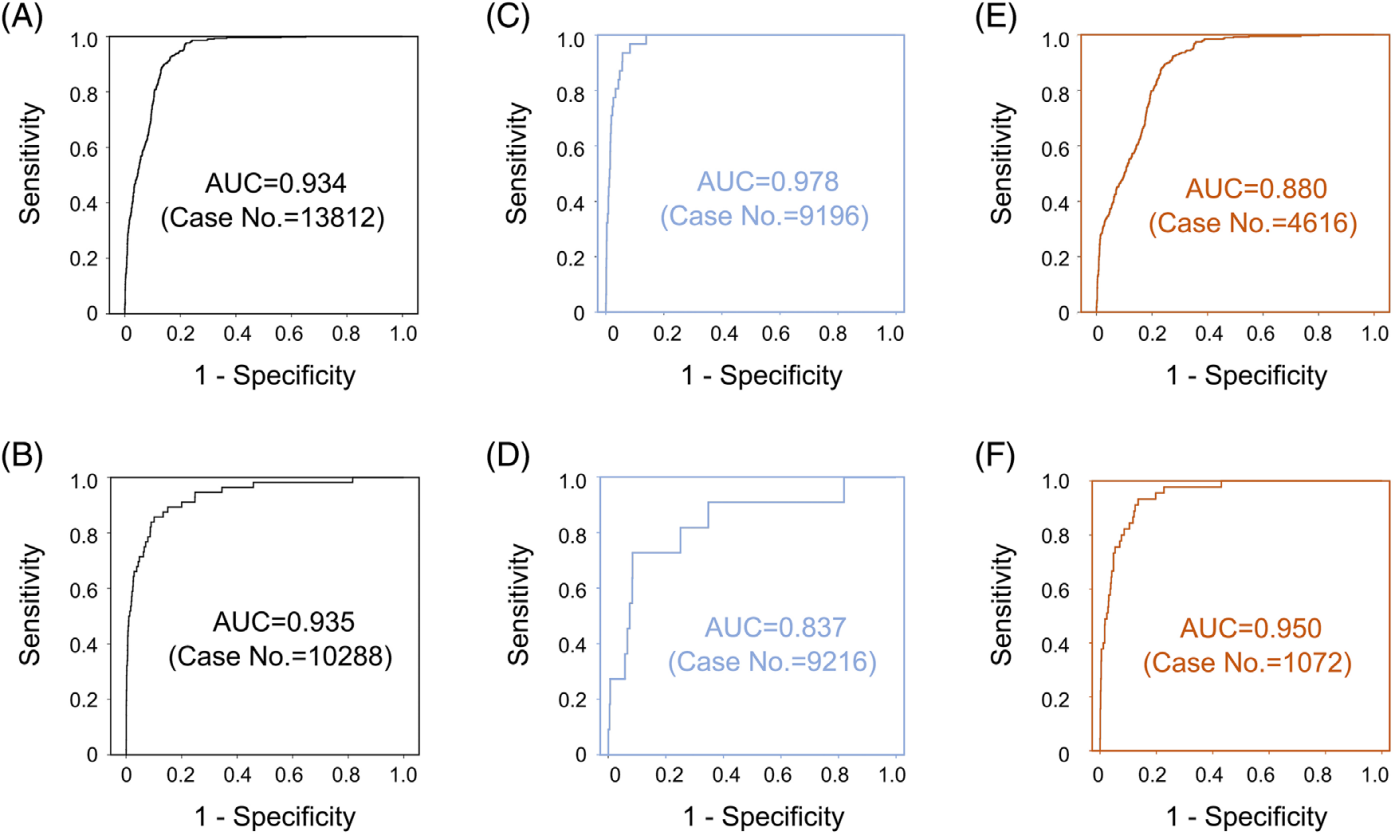
The ROC curves for evaluating the diagnostic performance of p16^INK4A^ FCM. (A) p16^INK4A^ FCM's ROC performance for detecting high‐grade lesions in women with higher‐than‐normal expression levels of p16^INK4A^ (positive ratios ≥14.27%). (B) p16^INK4A^ FCM's ROC performance for detecting high‐grade lesions in women with lower‐than‐normal expression levels of p16^INK4A^ (positive ratios < 14.27%). (C) p16^INK4A^ FCM's ROC performance for detecting high‐grade lesions in HPV‐negative women with higher‐than‐normal expression levels of p16^INK4A^. (D) p16^INK4A^ FCM's ROC performance for detecting high‐grade lesions in HPV‐negative women with lower‐than‐normal expression levels of p16^INK4A^. (E) p16^INK4A^ FCM's ROC performance for detecting high‐grade lesions in HPV‐positive women with higher‐than‐normal expression levels of p16^INK4A^. (F) p16^INK4A^ FCM's ROC performance for detecting high‐grade lesions in HPV‐positive women with lower‐than‐normal expression levels of p16^INK4A^. For each population of women, the area under the curve (AUC) was calculated, and the case number was given.

Women with abnormal Pap results (in either HPV‐positive or HPV‐negative conditions; see Figure 2) were enrolled to test the quantitative relationship between p16^INK4A^‐positive ratios and Pap pathomorphological changes as well as to evaluate the performance of p16^INK4A^ FCM in diagnosing neoplastic lesions (using colposcopy‐guided biopsy as a gold standard). According to the age‐ and HPV infection‐dependent distributional patterns of the aforementioned 17562 (HPV‐negative, NILM) and 3596 cases (HPV‐positive, NILM), age‐ and viral genotype‐matched women with abnormal Pap tests were assessed and comprised each study cohort based on TBS categories (see Tables S2, S4 and S5 for the demographic, clinical and pathological characteristics of the enrolled women). The NILM cohorts were used as negative controls to scale the lesion‐specific p16^INK4A^ increments in HPV‐positive and HPV‐negative conditions. The FCM‐detected p16^INK4A^‐positive ratios were significantly higher in Pap‐abnormal women than in NILM women (NILM women vs. non‐NILM women: 13.9 ± 1.8 vs. 17.7 ± 5.0–21.4 ± 7.2% in HPV‐negative women and 15.1 ± 1.6 vs. 18.0 ± 5.2–20.0 ± 9.9% in HPV‐positive women; Table S6). For each TBS category, an age‐dependent distributional pattern of p16^INK4A^ increment was observed (Table S6), while the age group with peak increment varied among the cohorts (Figures 4A and B). For HPV‐positive Pap‐abnormal women, the average increments of p16^INK4A^‐positive ratios were relatively higher in those with ASC‐US/‐H conditions (6.0 and 6.3%, respectively) than in those with HSILs (5.6%) and were lowest in those with LSILs (4.1%; Figure 4C and Table S6). However, for women with negative HPV DNA tests, although the p16^INK4A^ increments were higher in those with ASC‐US/‐H (5.5 and 6.4%, respectively) than in those with LSILs (3.9%), the detected increments reached the highest (7.5%) in those with HSILs, justifying the utility of p16^INK4A^ as a signature for triaging women with high‐grade lesions in this subsituation (Figure 4C). We then explored the possible factors contributing to the paradoxical increments observed in HPV‐positive ASC‐US/‐H cases (Table S6). The scatter plot revealed that HPV‐positive high‐grade lesions (i.e., HSILs) were more frequently associated with lower‐than‐normal levels of p16^INK4A^‐positive ratios (i.e., <p16^INK4A^ physiological expression level 3 standard deviation; Figure 4D), and the number of these cases increased with the severity of Pap abnormalities (Figure 4D), leading to a decreased average p16^INK4A^ increment in LSIL/HSIL cases (Figures 4C and D). To evaluate the performance of p16^INK4A^ FCM for predicting biopsy results of the subsequent colposcopy, we adopted age‐adjusted p16^INK4A^‐positive ratios (normalised to the age group of 40–49 years) and applied a double‐cut‐off‐ratio criterion (Table 3 and Figure 5). The optimal cut‐offs were 18.0% for p16^INK4A^ overexpression cases and 11.4% for lower‐than‐normal cases (both related to a maximal Youden's index in their respective populations according to the definition described in section *Methods* and Figures 5A and B). Using this criterion, the clinical accuracy (assessed by Youden's index) in predicting histological HSILs (biopsy‐confirmed) was improved by 5.3% (p16^INK4A^ FCM vs. ASC‐US), 21.7% (p16^INK4A^ FCM vs. LSIL), 57.0% (p16^INK4A^ FCM vs. ASC‐H) and 60.9% (p16^INK4A^ FCM vs. HSIL) (Table 3, also Table S7a). Alternatively, as an HPV DNA test‐combined p16^INK4A^ FCM criterion was adopted, the optimised cut‐offs of the p16^INK4A^‐positive ratio were 18.3% (upper cut‐off) and 11.9% (lower cut‐off) for HPV‐positive cases and 18.6% (upper cut‐off) and 11.2% (lower cut‐off) for HPV‐negative cases, by which a maximal Youden's index of 0.78 was obtained, superior to that of the HPV DNA and Pap co‐test by 5.6% (Table 3 and Figures 5C–F). In total, 90.5% of HPV DNA test‐unidentified and 100% of Pap‐unidentified HSIL cases were recognised by p16^INK4A^ FCM using the HPV DNA‐combined criterion (Tables 3, also Tables S6 and S7).

**TABLE 3:** Diagnostic efficacy of p16^INK4A^ FCM for colposcopy referral and its comparison with known HSIL+‐triaging strategies.

Risk‐assessing criteria^a^	Colposcopy referral rates^b^ (%)	Sensitivity (%)	Specificity (%)	PPV (%)	NPV (%)	LR+	LR‐	Youden's Index^c^
The single‐cut‐off‐ratio strategy p16^INK4A^‐positive ratio ≥17.2%	13.9	86.0	87.5	12.2	99.7	6.9	6.2	0.73*
The double‐cut‐off‐ratio strategy p16^INK4A^‐positive ratio ≥18.0% or p16^INK4A^‐positive ratio < 11.4%	15.0	90.3	86.5	12.0	99.8	6.7	9.0	0.77*
The HPV DNA‐combined double‐cut‐off‐ratio strategy p16^INK4A^‐positive ratio ≥18.6% or p16^INK4A^‐positive ratio < 11.2% (in the HPV‐negative condition) p16^INK4A^‐positive ratio ≥18.3% or p16^INK4A^‐positive ratio < 11.9% (in the HPV‐positive condition)	13.0	89.5	88.5	13.6	99.8	7.8	8.5	0.78
HPV status								
Any HPV DNA positive	23.6	91.2	77.8	7.7	99.8	4.1	8.9	0.69*
HR HPV DNA positive^d^	22.5	90.2	78.9	8.0	99.7	4.3	8.0	0.69*
HPV‐16 and/or ‐18 positive	10.5	52.5	90.3	9.9	98.9	5.4	1.9	0.43*
Pap test								
≥ASC‐US	12.2	83.9	89.2	13.6	99.6	7.8	5.5	0.73*
≥LSIL	5.0	60.7	96.1	23.9	99.2	15.5	2.4	0.57*
≥ASC‐H	0.6	21.5	99.9	76.3	98.4	159.1	1.3	0.21*
≥HSIL	0.4	17.6	99.9	80.8	98.4	207.6	1.2	0.17*
HPV DNA and Pap co‐test Any of the followings: (1) HPV‐16 and/or ‐18 positive (2) HR HPV DNA positive and Pap ≥ASC‐US (3) HR HPV DNA negative and Pap ≥ASC‐H (as per ASCCP guidelines)	15.0	86.0	86.4	11.3	99.7	6.3	6.2	0.72*

^a^ The criteria listed above were intended to assess the immediate risk of histological HSIL+ in the enrolled population (HPV‐positive/HPV‐negative and Pap‐normal/abnormal women, 24 100 in total) and to triage the high‐risk women for colposcopy and biopsy (if necessary). HPV DNA and Pap tests were currently widely used HSIL+ (CIN2/3+)‐triaging strategies in the clinic, and their combinations were the standard cervical cancer risk assessment method as suggested by the ASCCP 2012 and 2019 guidelines.

^b^ The term ‘colposcopy referral rate’ indicates the percentage of HSIL+ high‐risk women identified by a triage strategy in the enrolled population who need to undergo colposcopy and biopsy (if necessary) to confirm or exclude the existence of a high‐grade lesion.

^c^ Diagnostic parameters, namely, sensitivity, specificity, positive predicative value (PPV), negative predicative value (NPV), positive likelihood ratio (LR+), negative likelihood ratio (LR−) and Youden's index, were compared seriatim between the HPV DNA‐combined double‐cut‐off‐ratio strategy (i.e., a reference strategy) and each of any other HSIL+‐triaging strategies using the two‐sided McNemar test. *, statistically significant.

^d^ The HR HPV DNA positive cases involved those with HPV‐16 and HPV‐18 infections.

### 3.5 FCM‐based p16^INK4A^‐positive ratios predicted 2‐year pre‐cancer/cancer risks in women with HPV infections and/or Pap abnormalities

**FIGURE 6:**
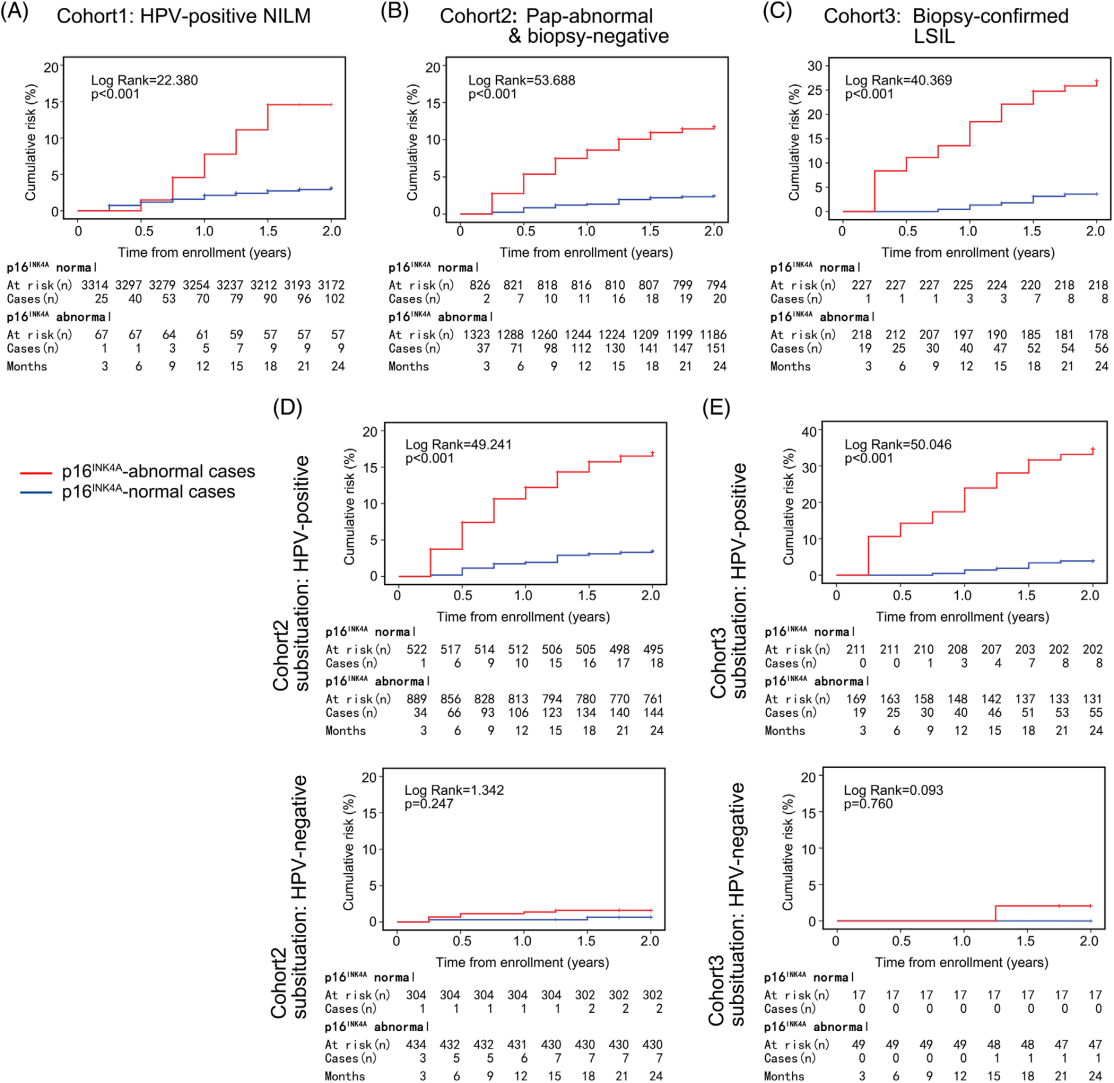
Prospective comparison of the 2‐year cumulative risks of histological HSIL+ between p16^INK4A^‐normal and p16^INK4A^‐abnormal women. (A) The 2‐year prospective cohort study comparing the cumulative histological HSIL risks of p16^INK4A^‐normal and p16^INK4A^‐abnormal women in the HPV‐positive NILM population (i.e., Cohort 1, total case number: 3407). (B) The 2‐year cohort study comparing the histological HSIL risks of p16^INK4A^‐normal and p16^INK4A^‐abnormal women in the Pap‐abnormal biopsy‐negative population (i.e., Cohort 2, total case number: 2188). (C) The 2‐year cohort study comparing the histological HSIL risks of p16^INK4A^‐normal and p16^INK4A^‐abnormal women in the biopsy‐confirmed LSIL population (i.e., Cohort 3, total case number: 465). (D) The subsituational analyses for Cohort 2, that is, comparing the histological HSIL risks in HPV‐positive (*n* = 1446) and HPV‐negative (*n* = 742) women. (E) The subsituational analyses for Cohort 3, that is, comparing the histological HSIL risks in HPV‐positive (*n* = 399) and HPV‐negative (*n* = 66) women. The log‐rank test was used for the statistical analyses.

There were both HPV‐positive and HPV‐negative women (1428 in total) with abnormal p16^INK4A^‐positive ratios who had negative colposcopy‐guided biopsy results (including 68 women with biopsy‐negative Pap‐normal results and 1360 women with biopsy‐negative Pap‐abnormal results). Additionally, there were women with biopsy‐confirmed LSILs (465 in total) who were followed up to 2 years as required by the American Society of Colposcopy and Cervical Pathology (ASCCP) guidelines. Therefore, we conducted a prospective study of these women to investigate whether they would run a higher risk of HSIL+ (i.e., HSIL and worse lesions, see Figure 2) in 2 years compared with their p16^INK4A^‐normal counterparts (i.e., 11.2–18.6% in the HPV‐negative condition or 11.9–18.3% in the HPV‐positive condition). As expected, for all three conditions, namely, the HPV‐positive Pap‐normal (NILM) condition, Pap‐abnormal biopsy‐negative condition and biopsy‐confirmed LSIL condition, women with an abnormal p16^INK4A^ status exhibited a significantly higher incidence rate of histological (i.e., biopsy‐confirmed) HSILs (Tables 4 and S8). In the cohorts studied, abnormal p16^INK4A^ status was consistently an independent prognostic determinant compared with other risk factors, namely, HPV DNA status, viral genotype, preceding Pap test and age at diagnosis, whose HRs remained at high levels (4.3–7.2‐fold relative to the ‘1.0’ reference) and were statistically significant in multivariate analyses (Tables 5, 6, 7). During the follow‐up period, for p16^INK4A^‐abnormal cases within HPV‐positive NILMs and Pap‐abnormal biopsy‐negative (subsituation: HPV‐positive) cohorts, their cumulative risks (PPVs at year 2: 13.2–15.6%) of histological HSILs exceeded the ASCCP‐recommended threshold for colposcopy referral (i.e., immediate risk of CIN3+ ≥4%), while in the biopsy‐confirmed LSIL cohort HPV‐positive subsituation, this risk was further doubled for p16^INK4A^‐abnormal women (21.3% at year 1, 29.3% at year 2; Figure 6 and Table 5, also Table S8), suggesting the necessity of early intervention for these women (considering that the threshold recommended by ASCCP for immediate treatment was an immediate risk of CIN3+ ≥25%). In our observation, only the women with HPV‐negative biopsy LSIL and Pap‐abnormal biopsy‐negative status with normal p16^INK4A^ expression displayed significantly lowered cumulative HSIL risks (PPVs at year 2: 0–0.7%), which were close to the ASCCP‐recommended ‘1‐year return’ threshold (i.e., 5‐year CIN3+ risk ≥0.55%) in 2 years (Table 5). However, for HPV‐positive women, either in the Pap‐normal or Pap‐abnormal cohorts (including two subsituations: biopsy‐negative and biopsy‐confirmed LSIL), we observed that the cumulative HSIL risks of women with a p16^INK4A^‐normal status persistently ascended and crossed the 1‐year return threshold at year 1 (PPVs at year 1: 1.4–2.1%) while remaining below the colposcopy referral threshold at year 2 (PPVs at year 2: 3.1–3.8%, Figure 6 and Table S8). Hence, differences in 2‐year outcomes between p16^INK4A^‐normal and p16^INK4A^‐abnormal women should be mainly attributed to prognostic performances of p16^INK4A^ among HPV‐positive women in the three cohorts studied (Figure 6).

**TABLE 4:** Prognostic value of p16^INK4A^ FCM in women with specific cervical pathological situations and HPV DNA statuses

	1‐year follow‐up					2‐year follow‐up				
Cohorts and subsituations	HSIL rates^a^ (%)	Sensitivity^b^ (%)	Specificity (%)	PPV (%)	NPV (%)	HSIL rates (%)	Sensitivity (%)	Specificity (%)	PPV (%)	NPV (%)
Cohort 1: HPV‐positive NILM	2.2	6.7	98.1	7.4	97.9	3.3	8.1	98.2	13.2	96.9
HR HPV‐infected women^c^	2.3	6.8	98.1	7.7	97.8	3.4	8.3	98.2	13.8	96.9
HPV‐16 and/or ‐18‐infected women	3.1	8.7	97.6	10.3	97.1	4.1	8.1	97.6	12.8	96.1
Cohort 2: Pap‐abnormal Biopsy‐negative	5.6	91.1	39.6	8.2	98.7	7.8	88.3	40.1	11.1	97.6
HPV‐positive women	8.0	91.4	38.6	11.5	98.1	11.2	88.9	39.3	15.6	96.6
HPV‐negative women	0.9	85.7	41.4	1.4	99.7	1.2	77.8	41.3	1.6	99.3
Cohort 3: Biopsy‐confirmed LSIL	9.2	93.0	53.3	16.9	98.7	13.8	87.5	54.9	23.6	96.5
HPV‐positive women	10.8	93.0	58.4	21.3	98.6	15.8	87.3	60.4	29.3	96.2
HPV‐negative women	0	NA^d^	25.8	0	100	1.5	100	26.2	2.0	100

^a^ The term ‘HSIL rate’ refers to the incidence rate of histological HSIL (biopsy‐confirmed) occurring within the women of the cohort studied or with a subsituation specifically indicated during the follow‐up period.

^b^ The parameters listed above, namely, sensitivity, specificity, PPV and NPV, reflect the performance of p16^INK4A^ FCM (normal range of FCM‐detected p16^INK4A^‐positive ratios: 11.2–18.6% in the HPV‐negative condition or 11.9–18.3% in the HPV‐positive condition) in predicting the occurrence of histological HSIL+ within 1 or 2 years.

^c^ The HR HPV‐infected cases included HPV‐16 and HPV‐18 infections.

^d^ This parameter could not be calculated because the incidence rate of histological HSIL+ was zero within the HPV‐negative women of Cohort 3 during the first‐year follow‐up. NA, not available.

**TABLE 5:** Univariate and multivariate analyses of the prognostic factors influencing the 2‐year HSIL+ outcomes of HPV‐positive NILM women (Cohort 1)

	2‐year outcomes		Univariate analysis^a^		Multivariate analysis	
Characteristics	Lesions < HSIL (*n* = 3296)^b^	Biopsy‐confirmed HSIL+ (*n* = 111)	HR (95% CI)	*p* Value	HR (95% CI)	*p* Value
Age (year)				.637	–	–
16–19	48 (1)	2 (2)	1 (reference)			
20–29	648 (20)	14 (13)	0.523 (0.119–2.303)			
30–39	1200 (36)	40 (36)	0.806 (0.195–3.334)			
40–49	876 (27)	36 (32)	0.987 (0.238–4.097)			
50–59	446 (14)	16 (14)	0.862 (0.198–3.748)			
60–69	72 (2)	3 (3)	1.007 (0.168–6.024)			
70–79	6 (0)	0 (0)	0 (0–∞)			
HPV genotype^c^				.028*		.041*
LR HPV	165 (5)	2 (2)	1 (reference)		1 (reference)	
HR HPV	1442 (44)	62 (56)	2.275 (0.553–9.366)		2.296 (0.558–9.450)	
HPV‐16 and/or ‐18	1689 (51)	47 (42)	3.490 (0.854–14.266)		3.416 (0.835–13.966)	
p16^INK4A^ quantification				<.001*		<.001*
Normal	3237 (98)	102 (92)	1 (reference)		1 (reference)	
Abnormal	59 (2)	9 (8)	4.540 (2.296–8.975)		4.309 (2.176–8.532)	

^a^ Prognostic factors were analysed separately (univariate) or in a group (multivariate) using the Cox regression model. HR, hazard ratio; CI, confidence interval; *, statistically significant.

^b^ The outcome data are presented as number (%). The percentages were calculated based on the number of women with a specific outcome in a column.

^c^ As for women with multiple infections, if there were any HR genotypes (except for HPV‐16 and HPV‐18) involved in the infection, it was classified as an HR HPV infection. Similarly, if either HPV‐16 or HPV‐18 was involved in the infection, it was classified as an HPV‐16/‐18 infection.

**TABLE 6:** Univariate and multivariate analyses of the prognostic factors influencing the 2‐year HSIL+ outcomes of Pap‐abnormal biopsy‐negative women (Cohort 2)

	2‐year outcomes		Univariate analysis^a^		Multivariate analysis	
Characteristics	Lesions < HSIL (*n* = 2017)^b^	Biopsy‐confirmed HSIL+ (*n* = 171)	HR (95% CI)	*p* Value	HR (95% CI)	*p* Value
Age (year)				.087	–	–
16–19	25 (1)	1 (1)	1 (reference)			
20–29	383 (19)	36 (21)	2.306 (0.316–16.816)			
30–39	764 (38)	51 (30)	1.661 (0.230–12.019)			
40–49	533 (27)	42 (25)	1.950 (0.268–14.167)			
50–59	263 (13)	33 (19)	3.021 (0.413–22.085)			
60–69	43 (2)	7 (4)	3.850 (0.474–31.291)			
70–79	6 (0)	1 (0)	3.721 (0.233–59.494)			
HPV genotype^c^				<.001*		<.001*
No HPV	733 (36)	9 (5)	1 (reference)		1 (reference)	
LR HPV	75 (4)	4 (2)	4.226 (1.302–13.724)		6.412 (1.924–21.368)	
HR HPV	675 (33)	84 (49)	9.569 (4.812–19.030)		10.529 (5.089–21.785)	
HPV‐16 and/or ‐18	534 (27)	74 (44)	10.501 (5.257–20.977)		11.005 (5.302–22.844)	
Pap test				<.001*		<.001*
ASC‐US	1341 (67)	123 (72)	1 (reference)		1 (reference)	
LSIL	671 (33)	45 (26)	0.741 (0.527–1.042)		0.892 (0.632–1.257)	
ASC‐H	5 (0.2)	2 (1)	3.642 (0.901–14.726)		11.298 (2.741–46.572)	
HSIL	0 (0)	1 (1)	53.157 (7.283–387.992)		297.389 (36.317–2435.209)	
p16^INK4A^ quantification				<.001*		<.001*
Normal	808 (40)	20 (12)	1 (reference)		1 (reference)	
Abnormal	1209 (60)	151 (88)	4.798 (3.010–7.650)		4.550 (2.832–7.311)	

^a^ Prognostic factors were analysed separately (univariate) or in a group (multivariate) using the Cox regression model. HR, hazard ratio; CI, confidence interval; *, statistically significant.

^b^ The outcome data are presented as number (%). The percentages were calculated based on the number of women with a specific outcome in a column.

^c^ As for women with multiple infections, if there were any HR genotypes (except for HPV‐16 and HPV‐18) involved in the infection, it was classified as an HR HPV infection. Similarly, if either HPV‐16 or HPV‐18 was involved in the infection, it was classified as an HPV‐16/‐18 infection. ‘No HPV’ refers to the HPV DNA‐negative status.

**TABLE 7:** Univariate and multivariate analyses of the prognostic factors influencing the 2‐year HSIL+ outcomes of biopsy‐confirmed LSIL women (Cohort 3)

	2‐year outcomes		Univariate analysis^a^		Multivariate analysis	
Characteristics	Lesions < HSIL (*n* = 401)^b^	Biopsy‐confirmed HSIL+ (*n* = 64)	HR (95% CI)	*p* Value	HR (95% CI)	*p* Value
Age (year)				.380	–	–
16–19	2 (1)	1 (2)	1 (reference)			
20–29	63 (16)	10 (16)	0.422 (0.054–3.295)			
30–39	156 (39)	32 (50)	0.539 (0.074–3.948)			
40–49	118 (29)	11 (17)	0.259 (0.033–2.006)			
50–59	53 (13)	8 (12)	0.401 (0.050–3.206)			
60–69	9 (2)	2 (3)	0.572 (0.052–6.307)			
HPV genotype^c^				.004*		.001*
No HPV	65 (16)	1 (2)	1 (reference)		1 (reference)	
LR HPV	16 (4)	2 (3)	7.762 (0.704–85.599)		14.135 (1.277–156.432)	
HR HPV	182 (45)	23 (36)	7.780 (1.051–57.610)		14.106 (1.897–104.894)	
HPV‐16 and/or ‐18	138 (35)	38 (59)	15.579 (2.139–113.470)		27.693 (3.784–202.651)	
Pap test				.011*		.008*
NILM	108 (27)	4 (6)	1 (reference)		1 (reference)	
ASC‐US	126 (31)	26 (41)	5.019 (1.751–14.380)		4.398 (1.518–12.741)	
LSIL	150 (37)	27 (42)	4.539 (1.588–12.972)		4.123 (1.430–11.891)	
ASC‐H	4 (1)	1 (2)	6.482 (0.724–58.007)		9.515 (1.041–86.945)	
HSIL	13 (3)	6 (9)	9.837 (2.775–34.868)		10.368 (2.915–36.876)	
p16^INK4A^ quantification				<.001*		<.001*
Normal	220 (55)	8 (12)	1 (reference)		1 (reference)	
Abnormal	181 (45)	56 (88)	7.508 (3.578–15.752)		7.222 (3.412–15.285)	

^a^ Prognostic factors were analysed separately (univariate) or in a group (multivariate) using the Cox regression model. HR, hazard ratio; CI, confidence interval; *, statistically significant.

^b^ The outcome data are presented as number (%). The percentages were calculated based on the number of women with a specific outcome in a column, which are given as an integer in the parentheses.

^c^ As for women with multiple infections, if there were any HR genotypes (except for HPV‐16 and HPV‐18) involved in the infection, it was classified as an HR HPV infection. Similarly, if either HPV‐16 or HPV‐18 was involved in the infection, it was classified as an HPV‐16/‐18 infection. ‘No HPV’ refers to the HPV DNA‐negative status.

## 4 DISCUSSION

For cervical cancer, the implication of p16^INK4A^ as a qualitative pathological signature has been fully documented. Previously, pathological and clinical guidelines and/or instructions were issued for labelling p16^INK4A^ on cytological (Pap) or histological (biopsy) slides with proper immunostaining techniques as well as for evaluating the severity of pre‐cancerous/cancerous lesions (CIN1, CIN2/3 and invasive cancer) with qualitative criteria of p16^INK4A^.^9^, ^10^, ^11^, ^12^, ^30^, ^31^, ^32^ Our current work extended p16^INK4A^ pathology to women with premorbid, ambiguous or transitional pathological statuses of cervical lesions (i.e., HPV‐positive Pap‐normal, biopsy‐negative Pap‐abnormal and biopsy‐confirmed LSIL), which enabled both immediate and short‐term (2‐year) risk assessments of high‐grade lesions in more specified and difficult clinical settings. Our data showed that p16^INK4A^ FCM (an in vitro diagnostic device, IVD) can be used to quantitatively and precisely determine the cervical p16^INK4A^ expression level, by which both age‐dependent physiological and infection‐induced aberrant expression of p16^INK4A^ can be measured; therefore, the related intracellular molecular changes upon HPV infection (as Pap is still normal) can be surveilled. These findings justify the utility of p16^INK4A^‐based quantitative pathology for detecting/predicting cervical carcinogenesis and directing early interventions (e.g., colposcopy, physical ablation, loop excision) among high‐risk women.

The history of p16^INK4A^ as an immunostaining signature for cervical pre‐cancer/cancer lesions has been 20 years.^9^ p16^INK4A^ was originally proposed to eliminate discrepancies between pathologists as they interpreted Pap or biopsy findings into TBS‐categorised terms. As the initial inventors of the p16^INK4A^ immunostaining technique, Klaes and his colleagues observed intensive and diffuse immunohistochemical staining of p16^INK4A^ in 100% (60 out of 60) of cases of HSIL and 97% (58 out of 60) of cases of invasive cancer.^9^ The authors therefore believed that this signature would ensure a significantly lowered misdiagnosis rate for detecting histological high‐grade lesions and a minimised overdiagnosis rate in inflammatory/hyperplastic conditions during microscopic examination. However, it was not until Bibbo et al., who developed a specific immunostaining protocol for securing overexpressed p16^INK4A^ to be labelled on thin‐layer, liquid‐based Paps, that the qualitative correlation between immunocytochemical staining of p16^INK4A^ and histological HSIL+ lesions was formally established.^33^ Despite its practical significance, p16^INK4A^ immunocytochemistry is still a labour‐intensive and skill‐demanding technique that requires pathologists to carefully discern truly stained cells from false‐positive cells under a nonspecific immunostaining background. Moreover, per Bibbo's approach, the semiquantitative count of true‐positive cells is a prerequisite for predicting HSIL+ lesions. Sahebali et al. subsequently created a holo‐quantitative scale for p16^INK4A^‐based detection of HSILs by counting the exact number of positive cells in a Pap, which obtained a 21.2% PPV at the 95% detection sensitivity.^10^ Notably, both Bibbo's and Sahebali's algorithms implicated a default principle, that is, the number (or ratio) of p16^INK4A^‐positive cervical cells should be proportional to the risk of high‐grade lesions. This principle can be summarised as a ‘pyramid theory’ with an emphasis that the cervical pre‐cancer/cancer should originate from a vast number of morphologically normal but molecularly altered cells (e.g., p16^INK4A^ overexpression). In this study, we applied this principle to triage potential lesions; nevertheless, our technique avoided the burden of manually counting p16^INK4A^‐positive cells under a microscope. The data we obtained were quite objective due to the nature of machine‐reading and automatic analysis of p16^INK4A^ FCM, which were also improved by the addition of external standards to calibrate this detection system (see section *Methods*). We, therefore, obtained a better diagnostic performance with p16^INK4A^ with a much higher Youden's index (0.78) compared with earlier works (0.26–0.56, regardless of HPV DNA status).^10^, ^33^, ^34^, ^35^, ^36^ In addition, compared with the lower PPVs of previous studies,^33^, ^34^, ^35^, ^36^ which were between 16.6 and 22.0%, our system exhibited a significantly improved HSIL risk‐assessing ability with a PPV at 25.0% among HPV‐positive women (Tables S7c), reflecting the importance of the precise quantification of p16^INK4A^‐positive cells rather than microscopically assessing their morphology.^10^

As a critical finding of this study, we observed that the physiological expression of p16^INK4A^ in the cervix varied with the woman's age. The overall expression of p16^INK4A^ was significantly lower in women aged 20–29 and 50–59 years than in women aged 30–39 and 40–49 years, suggesting that normal levels of female hormones (oestrogen, progesterone) might be essential for maintaining the physiological expression of p16^INK4A^, while for women at the menopausal period, diminished proliferation activity of the cervical epithelium was associated with decreased p16^INK4A^ expression (e.g., ≥50 years of age; Figure 3B). To our knowledge, this phenomenon has never been reported before, especially in studies that employed traditional immunochemical techniques. As early as the 2000s, research groups reported the existence of p16^INK4A^ physiological expression in cervical epithelial cells. This observation led them to define p16^INK4A^ overexpression as a count of ≥10‐15 positive cells in the Pap test.^10^, ^34^, ^35^, ^37^ However, earlier authors failed to eliminate the age‐related fluctuations caused by physiological p16^INK4A^‐positive cells, which could have influenced their diagnostic accuracy on HSIL+ lesions (Youden's indexes: 0.26–0.56 vs. our current work: 0.78). The possible reasons for their neglect might include that (1) they overlooked the importance of age‐related p16^INK4A^ expression in pursuing a proper cut‐off ratio for cervical neoplasia and that (2) the traditional techniques did not allow probing a tiny change in protein expression within a normal range or below the detection threshold (all such cases would be judged as negative). Owing to the precise quantification ability of p16^INK4A^ FCM, we characterised the age‐related variations in p16^INK4A^ expression in normal cervixes (i.e., HPV‐negative NILM cases). Therefore, directly mixing p16^INK4A^‐positive ratios of younger women (20‐29 years old) with those of elderly women (≥30 years old) would result in a significantly lowered cut‐off ratio to be set for neoplastic lesions, leading to raised false‐positive cases in age groups of 30–39 and 40–49 years. Such data would undermine the diagnostic accuracy of p16^INK4A^. We therefore adopted age‐adjusted p16^INK4A^ criteria and achieved an improved PPV for HSILs (Table 3). Moreover, we observed age‐dependent p16^INK4A^ increments after HPV infection. Our data showed that p16^INK4A^ increments in the HPV‐positive condition also varied with ages and viral genotype. Interestingly, the variations in p16^INK4A^ increments we detected could be used to support several known molecular theories of HPV infection. (1) First, in previous reports, the 5‐year cumulative incidence of HSIL+ presented a first peak at 11% in women aged 40–44 years and a second at 10% in women aged 60–64 years.^38^, ^39^, ^40^, ^41^ We similarly found a major peak of p16^INK4A^ increment in women aged 40–49 years and a minor peak in women aged 60–69 years (Figures 3B and C). In addition, in the literature, the HPV infection statuses of both age groups remained stable from the initial baseline investigation throughout the follow‐up period.^40^ Hence, the degree of p16^INK4A^ increments should be more closely associated with the occurrence of HSIL+ lesions than the persistence of HPV infection per se, reflecting the exacerbation of the internal tumour suppressive environment in age‐related high‐risk women. (2) Second, we found that women in the 20–29 years age group, who were reported to have the lowest risk of HSIL+ lesions, had a minimal increase in p16^INK4A^ after HPV infection. Additionally, current clinical experiences have indicated that HPV infections scarcely cause severe neoplastic changes in the cervixes of women aged 20–24 years.^38^, ^39^ We therefore confirmed that the carcinogenetic capability of HPVs could be measured by their ability to induce p16^INK4A^ dysregulation. (3) Third, after reviewing the literature, we noted that the peak serum oestrogen and progesterone concentrations emerge at ages 40–49 years,^42^ which was synchronised with peak p16^INK4A^ positive ratios/increments in the observed age group in our study (Figure 3 and Table S6). This phenomenon implies that sex hormones might play a role in accelerating cervical neoplasia formation. However, this inference does not apply to women aged >60 years, who accounted for nearly 20% of cervical cancer cases in previous reports.^39^, ^40^ More risk factors need to be weighed in this age group. Per recent advances in aetiologies of cervical cancer, the long‐term infection‐caused viral integration and aftermath disarrangement of the host genome could be an attributable reason.^43^, ^44^, ^45^, ^46^ In our work, this possibility has been partially reflected in the fact that the infection‐induced p16^INK4A^ increment peaked again in women aged 60–69 years (Figures 3B and C and Table S6b).

Our study described the practical performance of p16^INK4A^‐based quantitative pathology in identifying high‐grade cervical lesions. Similar to earlier studies focused on the application of the HPV DNA and p16^INK4A^ immunocytochemical co‐test for triaging women with higher risks of cervical cancer,^11^, ^12^, ^13^, ^14^, ^15^ we verified that the combination of p16^INK4A^ FCM and HPV DNA tests could be superior to the traditional HPV DNA and Pap co‐test in screening of HSILs with regard to their respective diagnostic parameters, namely, sensitivity (89.5 vs. 86.0%), specificity (88.5 vs. 86.4%), PPV (13.6 vs. 11.3%) and NPV (99.8 vs. 99.7%, respectively; Table 3). Moreover, we gained several lines of evidence that could influence current opinions on the molecular mechanisms of HPV persistent infection and carcinogenetic activity. (1) First, through p16^INK4A^ FCM, we established a statistical correlation between viral genotypes and p16^INK4A^‐positive ratios (or increments), where even a nuance of positive ratio/increment between single and multiple infections could be discriminated (Table S1). The data indicated that HPV‐16 and HPV‐18, the two most dangerous genotypes, induced the highest overexpression of p16^INK4A^, while the less dangerous HR and LR genotypes were associated with insufficient overexpression (Figure 3D). This finding corroborated previous works of Sahebali et al.^10^ and Sano et al.^47^ Sano et al.^47^ reported that strong staining of p16^INK4A^ could be predictive of HR HPV infection of the cervix (sensitivity 84%, specificity 98%, PPV 97%, NPV 86%). Sahebali et al.^10^ found that HPV‐16 induced a significantly higher number of p16^INK4A^ overexpression events than did other HR genotypes, while LR HPVs caused the lowest p16^INK4A^ overexpression. Therefore, both previous reports and our own data pointed to a common fact that p16^INK4A^, as a downstream element of RB, can be used to measure the harm an HPV infection could do to the host cells. The hazards of each HPV genotype, therefore, can be ranked per the p16^INK4A^ increments they have induced. However, we have other profound findings. We observed that an extra infection of less dangerous genotypes can interfere with the carcinogenetic capacity of a more dangerous genotype (Figure 3F and Table 2). Our epidemiological data also indicated that the immediate risk (i.e., PPV) of HSIL in women with multiple infections might be lower than that of women with a single infection of an HR HPV genotype (Table S7d). Similar findings have been reported by earlier studies, implying that multiple infections per se are not a pure accelerating factor for carcinogenesis.^48^, ^49^, ^50^, ^51^ (2) Second, we observed that for HPV‐negative women, those with HSILs had the highest p16^INK4A^ increments, whereas in HPV‐positive women, those with ASC‐US/‐H exhibited the highest p16^INK4A^ increments. Thus, a question was raised: Why does the performance of p16^INK4A^ in diagnosing HSILs become less sensitive in HPV‐positive women? Notably, Murphy et al.^52^ also reported that the intensity of p16^INK4A^ immunostaining was decreased in HPV‐positive women with histological HSILs (i.e., CIN2+), and in two of such cases, p16^INK4A^ immunochemistry was even negative. Similar p16^INK4A^‐negative HSIL+ cases have also been reported in other studies.^16^ For our own work, although a >90% sensitivity for p16^INK4A^ FCM in detecting HPV‐positive HSILs has been ensured after adopting a lower cut‐off ratio (Figure 5), more histologically normal or LSIL cases could be incorrectly diagnosed (triaged) as high‐grade lesions, and thus, these patients undergo excessive colposcopy, compromising the PPV of p16^INK4A^. A potential explanation for these paradoxical phenomena could be the fact that most women with p16^INK4A^‐negative HSILs had a lower‐than‐normal level of p16^INK4A^ expression (defined as ‘extremely low expression’). The existence of these cases lowered the overall expression level of p16^INK4A^ in HSIL cases and hence influenced the diagnostic accuracy of p16^INK4A^ FCM, especially when a single‐cut‐off‐ratio strategy was applied. Previously, a study conducted by Nuovo et al.^53^ indicated that aberrant promoter hypermethylation of the p16‐encoding gene CDKN2A is also a molecular trait of cervical neoplasia, which could occur at a very early stage of HPV infection. By this point, there should be two forces co‐propelling an initial lesion to a later worse lesion. One is the cell cycle dysregulation induced by the viral oncogenes E6 and E7, which has been manifested by p16^INK4A^ overexpression per se; the other is CDKN2A promoter hypermethylation, which could be reflected by the extremely low expression of p16^INK4A^. The latter produces a distinctive population of neoplastic lesions associated with a poor prognosis.^53^ In our study, we identified a group of women with HSILs and extremely low p16^INK4A^ expression by FCM‐based detection (note, transformation zone‐ or sampling‐related factors have been excluded; see Tables S6e and S6f). These cases could not be easily identified by traditional qualitative p16^INK4A^ techniques, such as immunochemistry, which could have been judged as negative. However, as the double‐cut‐off‐ratio criterion (i.e., p16^INK4A^‐positive ratios >18.0% or < 11.4%) was adopted, the sensitivity and Youden's index of p16^INK4A^ FCM were improved by 4.3% and 0.04, respectively (relative to the single‐cut‐off‐ratio strategy; Table 3). Therefore, unlike immunochemistry, FCM offered an opportunity to detect both overexpression and extremely low expression of p16^INK4A^. Future efforts to apply advanced analytic tools, for example, support vector machine (SVM), should be focused on creating a fitter nonlinear algorithm for p16^INK4A^ to predict more carcinogenesis events.

Our study investigated the prognostic value of p16^INK4A^ FCM in Pap‐/biopsy‐normal (or LSIL) populations. The roles of p16^INK4A^ in predicting women with cervicopathologically normal or low‐grade abnormal status are of interest to gynaecological clinicians worldwide. However, the outcomes of p16^INK4A^‐abnormal populations with different initial situations of HPV DNA, Pap and/or biopsy were not clear. Before 2008, Carozzi et al.^11^, ^12^ conducted a randomised controlled study (i.e., NTCC study) to investigate the efficacy of p16^INK4A^ in triaging biopsy‐negative/LSIL women threatened with increased risks of high‐grade lesions during 3‐year follow‐up. The design of the NTCC study was basically consistent with ours with a few exceptions: (1) in the NTCC study, only HPV‐positive women were referred to colposcopy, whereas in our work, both those with HPV infections and those with abnormal Pap results (even if HPV‐negative) underwent colposcopy; and (2) in the NTCC study, CIN2+ (i.e., histological HSIL+) cases detected by initial colposcopy and biopsy were mixed with those diagnosed during follow‐up for calculating p16^INK4A^ predictive values; however, in our study, cumulative risks of HSILs were calculated independent of initial colposcopy/biopsy examination results and were studied separately for three particular conditions (i.e., HPV‐positive Pap‐normal, biopsy‐negative Pap‐abnormal and biopsy‐confirmed LSIL), providing more detailed information for p16^INK4A^‐based prognosis. Carozzi et al.^11^, ^12^ indicated that p16^INK4A^ immunocytochemistry could improve both immediate and short‐term assessments for CIN2/3+ risks (sensitivity 91%, specificity 59%, PPV 20%, NPV 95%), and the calculated relative cumulative 3‐year risk for histological CIN2/3+ (HSIL+) lesions was 3.74 in p16^INK4A^‐positive women compared with negative controls. Later, in 2012, Wentzensen et al.^13^ performed a similar study in HPV‐positive Pap‐normal women by using a p16^INK4A^/Ki‐67 dual‐staining technique.^13^, ^14^ The authors supposed that an additional Ki‐67 counterstaining could ensure detected positive cells to be strictly epithelial cells but not contaminated inflammatory cells so that the p16^INK4A^ diagnostic specificity could be enhanced. They finally obtained a 5‐year risk assessment performance by dual‐staining for HSIL+ lesions with sensitivity 83%, specificity 59%, PPV 21% and NPV 96%, which were very close to the parameters reported by Carozzi et al.,^12^ implying a minimal contribution of Ki‐67 to the PPV and NPV improvements of p16^INK4A^ prognosis (approximately 1%). In our study, the 2‐year HSIL‐risk predictive performances of p16^INK4A^ differed with study cohorts. The sensitivity (100%) and NPV (100%) of p16^INK4A^ FCM reached the highest levels in the HPV‐negative biopsy‐confirmed LSIL women, while the highest specificity (98.2%) was found in the HPV‐positive NILM women. Furthermore, in the biopsy‐confirmed LSIL cases with positive HPV DNA tests, the PPV (i.e., 2‐year cumulative risk) of p16^INK4A^‐abnormal cases reached its highest level (29.3%), implying this particular population of women with LSILs had a much greater risk of HSIL+ than had been suggested by the ASCCP guidelines (i.e., 2.8–6.5%),^30^, ^31^ which, therefore, denied the rationality of the expectant management for these LSIL patients. Taken together, our study provided important evidence to support the prognostic significance of p16^INK4A^ to be interpreted on an individualised basis where both viral infection history and Pap/biopsy status need to be co‐weighed. This has mirrored the spirit of the 2019 version of the ASCCP guidelines.^31^

The main strengths of this study include the following: (1) the establishment of a novel p16^INK4A^ detection system, allowing the quantitative depiction of the expression level and pathological increment of p16^INK4A^ in cervical epithelial cells; (2) a large population‐based p16^INK4A^ quantification database, revealing the age‐ and viral genotype‐dependent expression of p16^INK4A^ in the cervix, necessitating an age‐ and HPV DNA test‐adjusted p16^INK4A^ diagnostic criterion to be made; (3) an improved performance in detecting/predicting high‐grade lesions in the cervix, allowing precise colposcopy referral and early intervention among high‐risk women; and (4) insights into the relationships between p16^INK4A^ quantification and the ASCCP guidelines, promoting evidence‐based decisions for treating women with ambiguous HPV and/or Pap results.

This study has limitations. (1) Due to the need to evaluate age‐ and HPV genotype‐specific expression of p16^INK4A^, we applied an age‐ and genotype‐matching strategy to enrol the study populations. Although HPV‐negative Pap‐normal cases were collected consecutively, the matched HPV‐positive Pap‐normal/abnormal cases were semi‐objectively enrolled, which might disrupt their natural time‐line continuity. (2) We did not determine the association between infection titre and p16^INK4A^ increment. Although the viral infection titres could be detected via quantitative PCR (qPCR), there are currently no United States Food and Drug Administration‐approved HPV DNA qPCR detection kit available worldwide. (3) No HPV‐negative Pap‐normal women were referred for colposcopy, and a few women with HPV‐negative HSIL might be misdiagnosed. However, considering that the number of such cases could be extremely low and the reported incidence was <0.04%,^11^, ^12^ our main findings, including the detection sensitivity and PPV of p16^INK4A^ FCM, should not be affected.

In summary, our work has developed the traditional immunochemistry‐based p16^INK4A^ qualitative pathology into a quantifiable and interlaboratory cross‐verifiable IVD, which was implemented through an FCM platform. This technological innovation built a bridge for pathologists to reach a more objective and consistent opinion on the nature of Pap abnormalities and inform clinicians of the molecular stage of HPV infection(s). The same technique can be applied to other known immunochemical signatures with clear clinical or pathological significance, such as Ki‐67, hTERT, EGFR, HER2 and ALK, which could provide more precise and quantitative information for pre‐therapy evaluation and post‐therapy surveillance.

## CONFLICT OF INTEREST STATEMENT

The authors declare no conflicts of interest.

## Supporting information

Supporting Information
