# Correction to “p16^INK4A^ flow cytometry of exfoliated cervical cells: Its role in quantitative pathology and clinical diagnosis of squamous intraepithelial lesions”

**DOI:** 10.1002/ctm2.70622

**Published:** 2026-02-15

He Y, Shi J, Zhao H, et al. p16^INK4A^ flow cytometry of exfoliated cervical cells: Its role in quantitative pathology and clinical diagnosis of squamous intraepithelial lesions. Clin Transl Med. 2023;13(3):e1209. doi:10.1002/ctm2.1209

In the list of authors, there are several spelling and/or typographical errors. The name of one author, Shanji Li, was wrongly spelt as “Shangji Li.” The superscript of another author, Yi Xia, should be 17, not 16. The name of the corresponding author, Jianlong Zhu, should be placed after the name of another corresponding author, Congjian Xu. Therefore, the list of authors should have read: “Yifeng He^1,2,3,*^, Jun Shi^1,*^, Hui Zhao^4^, Yuefei Wang^5,6^, Chi Zhang^4^, Sai Han^7^, Qizhi He^8^, Xiaolan Li^9^, Shanji Li^1^, Wenjing Wang^2^, Muhua Yi^10^, Xiaoling Hu^11^, Zhihua Xing^12^, Hao Han^12^, Yinshuang Gao^12^, Qing Zhou^13^, Linlin Lu^14^, Jianfen Guo^15^, Hui Cao^16^, Caiping Lu^16^, Yanqiang Hou^16^, Dan Chen^17^, Fengyun Yang^18^, Ping Lei^19^, Wen Di^1,2,20^, Ji Qian^21^, Yi Xia^17^, Youzhong Zhang^7^, Yang Deng^4^, Congjian Xu^5,6^, Jianlong Zhu^3^”

In the section “Correspondence,” the corresponding authors Jianlong Zhu and Congjian Xu should be accordingly re‐ordered, that is, the contact information of Jianlong Zhu should be placed after that of Congjian Xu. They should have read:

“Yifeng He”, Department of Obstetrics and Gynaecology, Ren Ji Hospital, School of Medicine, Shanghai Jiao Tong University, 160 Pujian Road, Shanghai 200127, China. Email: he_yifeng@hotmail.com.

Youzhong Zhang, Department of Obstetrics and Gynaecology, Qilu Hospital, Shandong University, 107 West Wenhua Road, Jinan, Shandong 250012, China. Email: zhangyouzhong@sdu.edu.cn

Yang Deng, Taiyuan Maternal and Child Health Care Hospital, Taiyuan, Shanxi 030000, China. Email: dengyang0717@163.com

Congjian Xu, Obstetrics and Gynaecology Hospital, Fudan University, 419 Fanxie Road, Shanghai 200011, China. Email: xucj@hotmail.com

Jianlong Zhu, Department of Obstetrics and Gynaecology, Pudong Hospital, Fudan University, 2800 Gongwei Road, Shanghai 200120, China. “Email: zhujianlong9999@sina.com”

In the section “Funding Information,” the grant from “Shanghai Pudong New District Health Commission, Grant/Award Number PW2022D‐06” should be added. This section should have read: “Shanghai Pudong New District Health Commission, Grant/Award Number PW2022D‐06; Shanghai Municipal Science and Technology Commission, Grant/Award Numbers: 15441905700, 15DZ1940502 and 17441908000; Natural Science Foundation of China, Grant/Award Number: 81572548.”

In the section “Abstract” of the text “Herein, we created a high‐throughput, quantitative diagnostic device, p16INK4A flow cytometry (FCM) and assessed its performance in cervical cancer screening and prevention,” the term “p16INK4A” was incorrectly typed. It should have read “p16^INK4A^.”

In the section “Acknowledgements,” the text “This work was supported by grants from the Science and Technology Commission of Shanghai Municipality (Nos. 15441905700, 15DZ1940502 and 17441908000) and the Natural Science Foundation of China (No. 81572548)” was incorrect. This should have read: “This work was supported by grants from the Shanghai Pudong New District Health Commission (No. PW2022D‐06), the Shanghai Municipal Science and Technology Commission (Nos. 15441905700, 15DZ1940502 and 17441908000) and the Natural Science Foundation of China (No. 81572548).”

We apologise for these errors.

