# Supplementary material for: p16INK4A flow cytometry of exfoliated cervical cells: Its role in quantitative pathology and clinical diagnosis of squamous intraepithelial lesions
Source: Clin Transl Med. 2023 Mar 7;13(3):e1209. doi: 10.1002/ctm2.1209 (PMC9991008; doi:10.1002/ctm2.1209)
Supplement: Supplementary file 1 — Supporting Information [file CTM2-13-e1209-s001.docx]

**p16^INK4A^ flow cytometry in cervical exfoliated cells: its role in quantitative pathology and clinical diagnosis for
squamous intraepithelial lesions**

**Supporting Information**

**Authors**

Yifeng He, Jun Shi, Hui Zhao, Yuefei Wang, Chi Zhang, Sai Han, Qizhi He, Xiaolan Li, Shangji Li,
Wenjing Wang, Muhua Yi, Xiaoling Hu, Zhihua Xing, Hao Han, Yinshuang Gao, Qing Zhou,
Linlin Lu, Jianfen Guo, Hui Cao, Caiping Lu, Yanqiang Hou, Dan Chen,
Fengyun Yang, Ping Lei, Wen Di, Qi Qian, Yi Xia,
Youzhong Zhang, Yang Deng, Jianlong Zhu, Congjian Xu

**Contents**

[Figure S1. 2](#_Toc108938453)

[Figure S2. 3](#_Toc108938454)

[Figure S3. 4](#_Toc108938455)

[Figure S4. 5](#_Toc108938456)

[Table S1. 6](#_Toc108938457)

[Table S2a-b 7](#_Toc108938458)

[Table S3a-e 9](#_Toc108938459)

[Table S4. 18](#_Toc108938460)

[Table S5a-b 19](#_Toc108938461)

[Table S6a-f 21](#_Toc108938462)

[Table S7a-d 27](#_Toc108938463)

[Table S8a-e 32](#_Toc108938464)

| 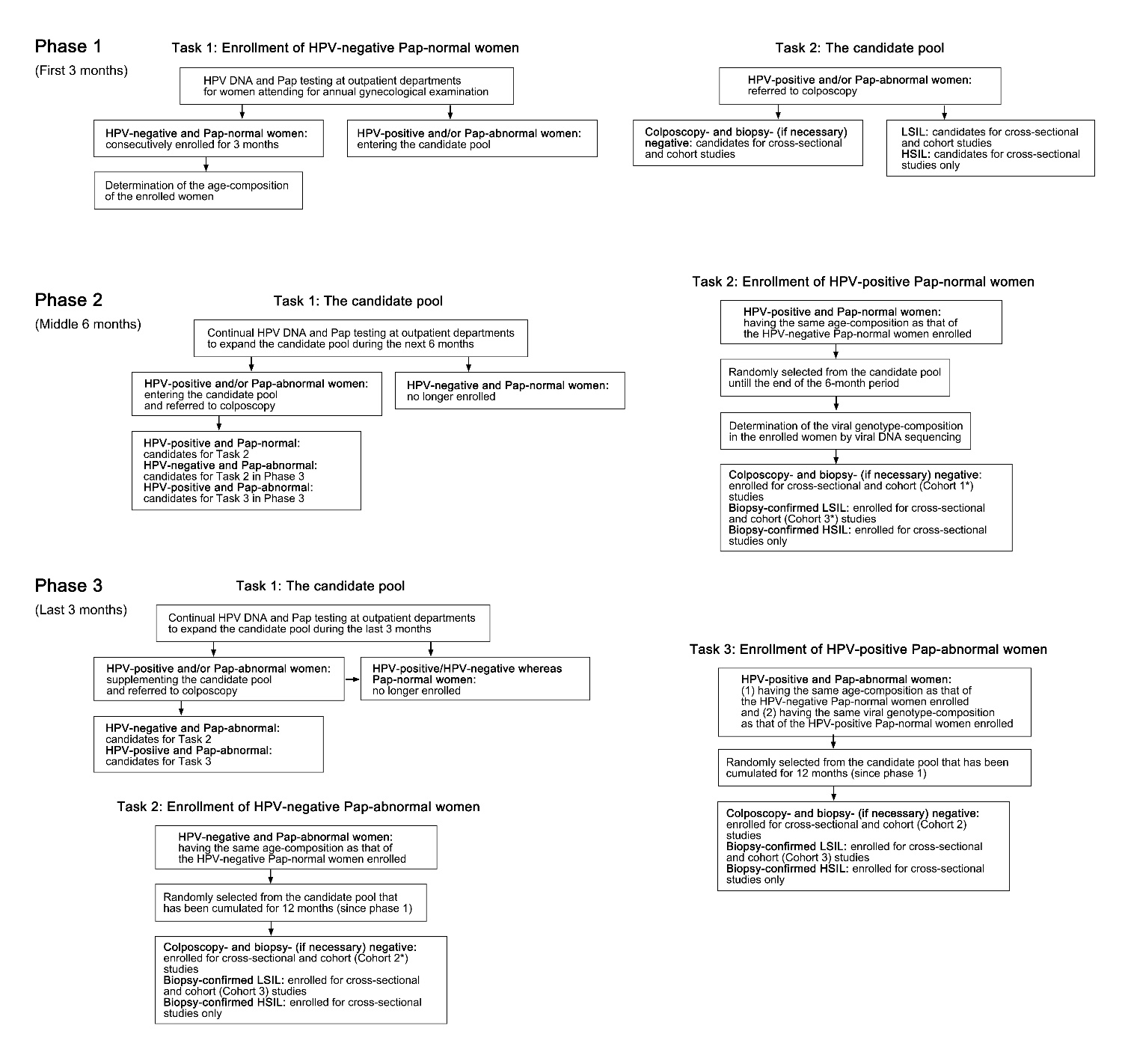 |
| --- |
| Figure S1. p16^INK4A^ FCM study protocol at the enrollment stage  The process of enrolling the study population contained three phases. Phase 1, HPV-negative Pap-normal women were consecutively enrolled at ten medical centers nationwide; in addition, a candidate pool that involved HPV-positive and/or Pap-abnormal women was established. Phase 2, the age-matched HPV-positive Pap-normal women were selected from the candidate pool, and the pool was further expanded. Phase 3, the age-matched HPV-negative Pap-abnormal women and the age- and viral genotype-matched HPV-positive Pap-abnormal women were selected from the candidate pool. The pool was immediately closed as the enrollment task had been fulfilled.  Because the HPV-negative Pap-normal women comprised the main body of the outpatient and gynecological examination population, who could easily meet the demand of enrolling an enough number of normal controls (Task 1), our focus was then put on how to collect an enough number of age- and viral genotype-matched HPV-positive and/or Pap-abnormal women (see Tasks 2 of Phases 2 and 3 and Task 3 of Phase 3). The second task (Task 2) of Phase 1 was to prepare a candidate pool for enrolling such women. Continual expansion of the candidate pool was a critical work during the entire enrollment process, which was also listed as Task 1 in Phase 2 and Phase 3.  We did not apply any intervention measurements to the candidates; and all women were routinely referred to colposcopy (indications for colposcopy referral adopted by most gynecological units in China include HPV infection per se and/or an abnormal Pap). We only recorded their medical histories, data of HPV DNA and Pap tests and biopsy pathological diagnoses. These data were used for the cross-sectional diagnostic studies.  The initially enrolled women, if appropriate, were subenrolled in the subquent 2-year cohort studies. In the three observational prospective studies, the cohorts were established by subenrolling an enough number of HPV-positive Pap-normal (Cohort 1), biopsy-negative Pap-abnormal (Cohort 2) and biopsy-confirmed LSIL (Cohort 3) women, respectively. For instance, if women enrolled in an initial cross-sectional study were biopsy-negative, they were then subenrolled into Cohort 1 or 2; and if they were diagnosed as histological LSILs, they were subenrolled into Cohort 3; and etc. However, if there were any women confirmed as histological HSILs (hHSILs), they would be immediately treated and disenrolled from further investigations. *, see Figure S2 for details. |

| 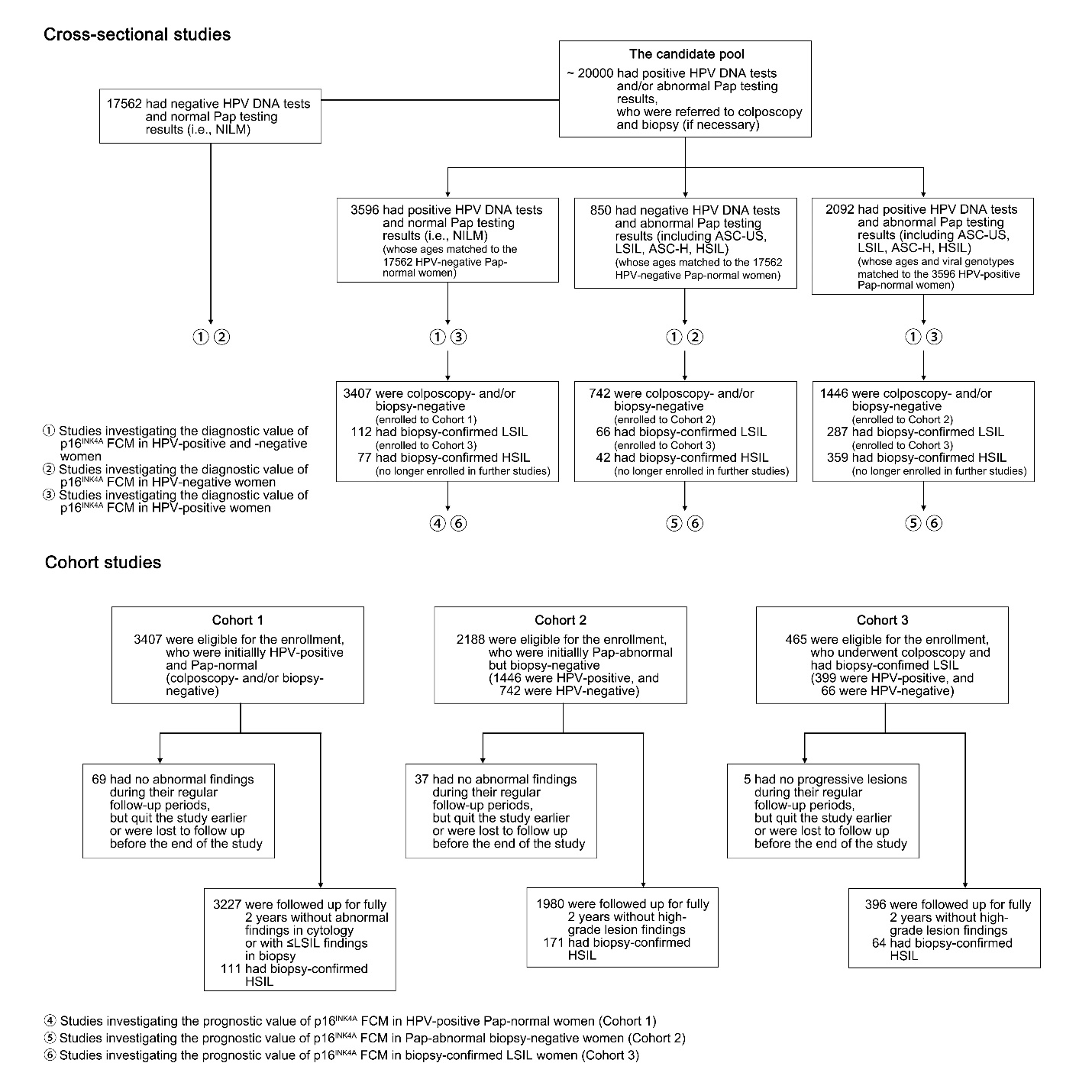 |
| --- |
| Figure S2. p16^INK4A^ FCM study protocol at the research stage  Total numbers of women in the HPV-negative Pap-normal, HPV-positive Pap-normal, HPV-negative Pap-abnormal and HPV-positive Pap-abnormal populations as well as those of women entering the three study cohorts are listed. The entire study comprised two tiers of investigations. One was the cross-sectional investigation to determine the diagnostic parameters of p16^INK4A^ FCM for histological HSIL+; the other was the observational prospective investigation of the 2-year prognostic values of p16^INK4A^ FCM for three cervical pathological situations, namely, HPV-positive Pap-normal, biopsy-negative Pap-abnormal and biopsy-confirmed LSIL. The prospective study was conducted in parallel among the three cohorts.  The cross-sectional diagnostic tests (studies) involved three rounds of investigations (analyses), namely, the analysis of diagnostic performance of p16^INK4A^ FCM in HPV-positive and HPV-negative women (refer to ①), the analysis of diagnostic performance of p16^INK4A^ FCM in HPV-negative women (refer to ②), the analysis of diagnostic performance of p16^INK4A^ FCM in HPV-positive women (refer to ③). To perform analysis ①, the data of all 24100 women were combined together. To perform analysis ②, the data of 17562 HPV-negative Pap-normal and 850 HPV-negative Pap-abnormal women were combined. To perform analysis ③, the data of 3596 HPV-positive Pap-normal and 2092 HPV-positive Pap-abnormal women were combined.  In cohort studies, HPV-positive Pap-normal biopsy-negative women subenrolled by Cohort 1 (refer to ④) were selected from the 3596 HPV-positive Pap-normal women. And those enrolled in Cohort 2 (refer to ⑤) were from the 850 HPV-negative Pap-abnormal women and 2092 HPV-positive Pap-abnormal women, who have undergone colposcopy and were histopathologically normal. Those subenrolled in Cohort 3 (refer to ⑥) were the ones selected from the initially enrolled HPV-positive/negative Pap-normal/abnormal women who were diagnosed as LSILs by colposcopy-guided biopsy. The numbers of women who were lost to follow-up and those who were confirmed to be hHSILs have been seriatim listed for each cohort. |

| 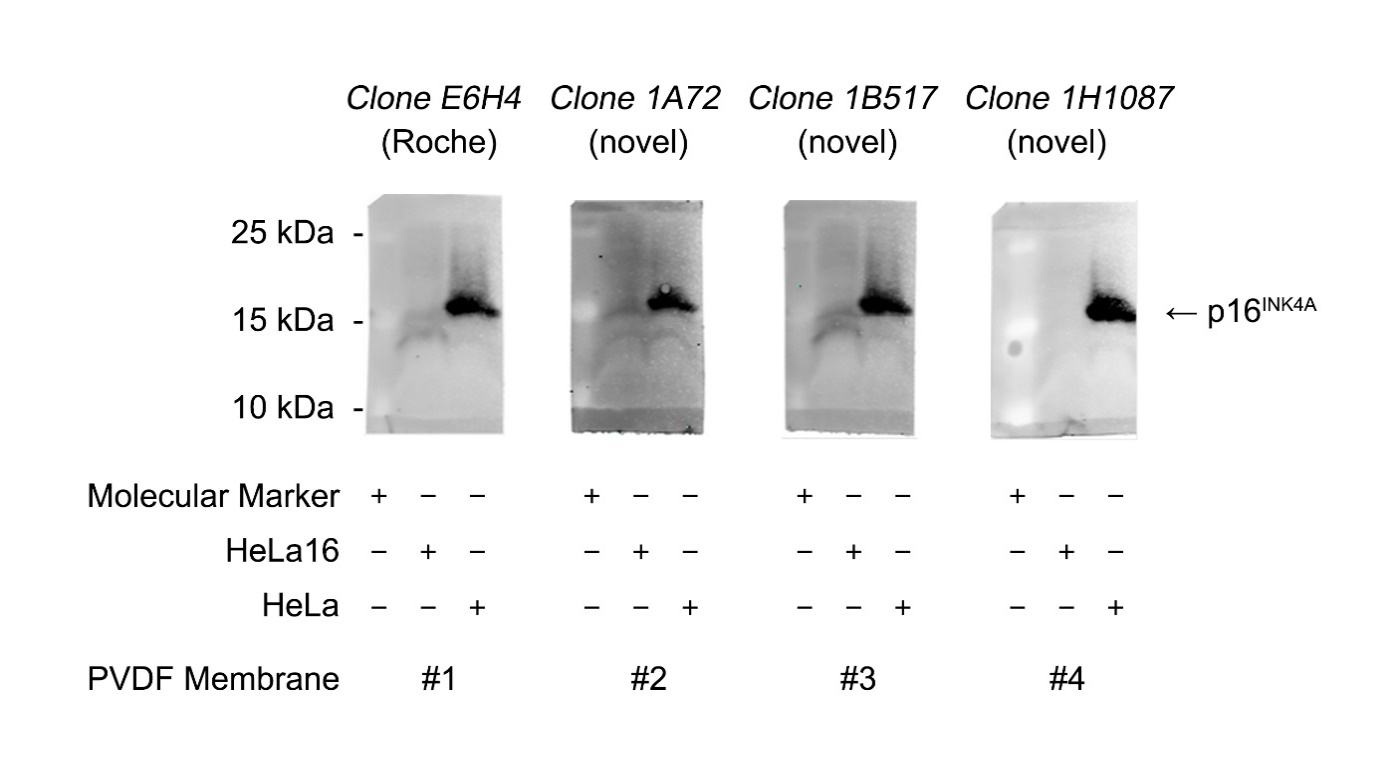 |
| --- |
| Figure S3. The immunoreactivities of commercial and newly prepared p16^INK4A^-specific antibodies  Immunoreactivities of the commercially available and newly prepared clones of human p16^INK4A^-specific antibodies were examined by Western blotting. #1, the E6H4 clone of p16^INK4A^-specific antibody was purchased from Roche. #2 - #4, the novel p16^INK4A^-specific antibodies, namely, clones 1A72, 1B517 and 1H1087 were prepared from mouse hybridomas. The whole-cell protein extracts of HeLa and HeLa16 cells were used as p16^INK4A^-positive and p16^INK4A^-negative standards, respectively. |

| 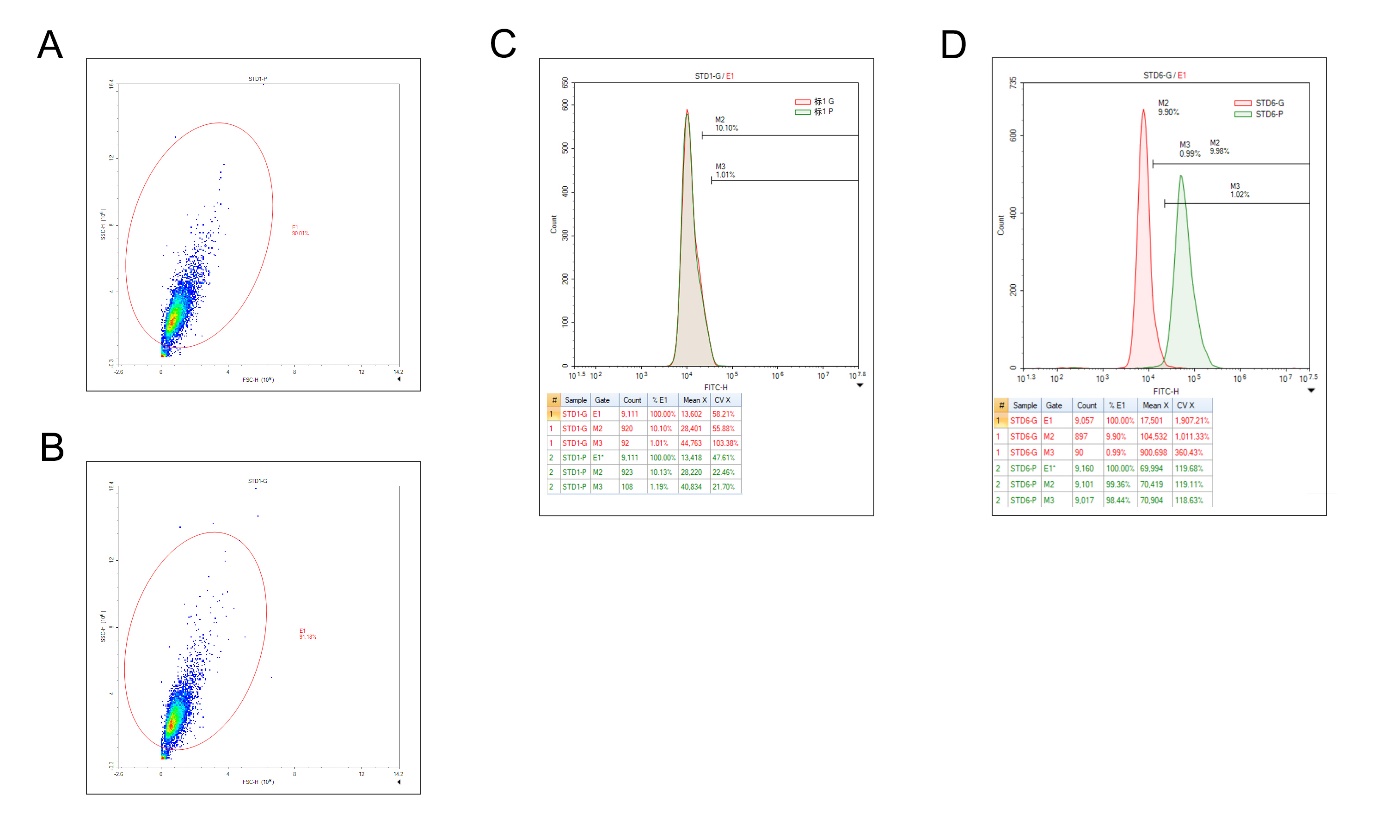 |
| --- |
| Figure S4. FCM analysis of the cytological samples  A. The scatter plot of flow cytometer-detected cells. The cells (i.e., the test portion) were sequentially labeled with a mouse antihuman p16^INK4A^ monoclonal antibody and an FITC-conjugated rabbit antimouse IgG monoclonal antibody. A group of detected cells were properly selected by the primary gate (E1) as candidates for the next analysis.  B. The scatter plot of cells (i.e., the reference portion) labeled only with the FITC-conjugated rabbit antimouse IgG monoclonal antibody. The gate used here was the same as that for the test portion of the sample cells.  C. The plot combined two cell number vs. p16^INK4A^-signal intensity two-dimensional curves; one (red) is the curve reporting the FCM results of the test portion of the sample cells; the other (green) is the curve reporting the FCM results of the reference portion of the sample cells. The 10% and 1% gates (M2 and M3) were set to determine the p16^INK4A^-positive ratios at the reference cutoff = 10% and 1%, respectively. Note that the tested sample cells were made of a 1-mL HeLa/HeLa16 mixture, which was used as a negative standard [HeLa/(HeLa+HeLa16) = 0].  D. The plot of cell number vs. p16^INK4A^-signal intensity two-dimensional curves reporting the FCM results for a positive standard [HeLa/(HeLa+HeLa16) = 1]. The peaks of the two curves were seperate. The data sheet beneath the plot showed that the M2 gate cuts 10.0% of the reference portion of the sample cells into its area, while for the test portion of the sample cells, 99.4% were in the gate (i.e., p16^INK4A^-positive ratio = 99.4% @ reference cutoff = 10%). As for the M3 gate, the in-gate percentages were 1.0% (green, the reference portion) and 98.4% (red, the test portion; i.e., p16^INK4A^-positive ratio = 98.4% @ reference cutoff = 1%). |

| Table S1. The resolution capability of p16^INK4A^ FCM relative to a background level of p16^INK4A^-positive ratios (mimicked using HeLa/HeLa16 mixtures) ^a^ | | | | | | | | |
| --- | --- | --- | --- | --- | --- | --- | --- | --- |
| **Background p16^INK4A^- positive ratios** ^a^ | **FCM values** ^b^  (for standard cell mixtures) | **Δ1%** | | **Δ0.1%** | | | **Δ0.01%** | |
|  |  | **FCM values** | **p value** ^c^ | **FCM values** | **p value** | | **FCM values** | **p value** |
| **Reference cutoff=10%** ^c^ | | | | | | | | |
| 75% | 64.5%±1.1% | 65.3%±1.0% | 0.028* | 64.6%±1.0% | | 0.817 | 64.5%±0.9% | 0.996 |
| 50% | 45.3%±0.8% | 45.9%±0.9% | 0.018* | 45.3%±0.8% | | 0.759 | 45.3%±0.7% | 0.959 |
| 25% | 28.2%±0.4% | 28.7%±0.3% | <0.001* | 28.3%±0.3% | | 0.413 | 28.2%±0.3% | 0.527 |
| 10% | 19.3%±0.5% | 19.9%±0.4% | <0.001* | 19.6%±0.2% | | 0.033* | 19.3%±0.5% | 0.833 |
| 0% | 10.2%±1.2% | 13.5%±1.1% | <0.001* | 11.4%±1.1% | | 0.002* | 10.6%±1.2% | 0.319 |
| **Reference cutoff=1%** | | | | | | | | |
| 75% | 58.8%±1.2% | 60.0%±0.6% | <0.001* | 59.1%±0.5% | | 0.234 | 59.1%±0.5% | 0.300 |
| 50% | 41.9%±0.8% | 42.4%±0.6% | 0.027* | 42.0%±0.7% | | 0.610 | 41.9%±0.9% | 0.797 |
| 25% | 25.5%±0.7% | 26.3%±0.5% | <0.001* | 25.7%±0.6% | | 0.313 | 25.6%±0.3% | 0.801 |
| 10% | 13.2%±0.5% | 14.1%±1.0% | <0.001* | 13.5%±0.4% | | 0.017* | 13.2%±0.3% | 0.914 |
| 0% | 1.0%±0.4% | 5.5%±1.4% | <0.001* | 2.2%±0.6% | | <0.001* | 1.1%±0.5% | 0.604 |
| **Reference cutoff=0.1%** | | | | | | | | |
| 75% | 14.5%±1.1% | 14.8%±1.0% | 0.465 | 14.7%±1.0% | | 0.743 | 14.6%±1.1% | 0.841 |
| 50% | 8.8%±1.0% | 9.0%±0.9% | 0.511 | 8.9%±1.0% | | 0.694 | 8.8%±0.9% | 0.978 |
| 25% | 4.5%±0.3% | 5.0%±0.2% | <0.001* | 4.8%±0.2% | | 0.001* | 4.5%±0.5% | 0.713 |
| 10% | 2.3%±0.2% | 2.6%±0.1% | <0.001* | 2.3%±0.1% | | 0.273 | 2.3%±0.1% | 0.574 |
| 0% | 0.1%±0.1% | 0.6%±0.1% | <0.001* | 0.3%±0.1% | | <0.001* | 0.2%±0.1% | <0.001* |
| a. This is a dataset supplementary to Figure 1D. To determine the detection resolution capabilities of p16^INK4A^ FCM at a variety of background levels of p16^INK4A^-positive ratios, a series of standard HeLa/HeLa16 mixtures and their related minimal increment samples were prepared and tested. The experiments were performed independent of the work in Figure 1D. The minimal increment test for each standard mixture [HeLa/(HeLa+HeLa16) ratios: 0%, 10%, 25%, 50% and 75%] included a 1%-, 0.1%- and 0.01%-increment sample, where 1%, 0.1% and 0.01% HeLa cells were respectively added into the original standard HeLa/Hela16 mixture with the ratio indicated. FCM experiments for each minimal increment sample at each background HeLa/(HeLa+HeLa16) ratio were repeated 20 times.  b. Data are presented as the mean ± SD. For each increment, the obtained p16^INK4A^ FCM data were given for three referent cutoffs, i.e., 10%, 1% and 0.1%.  c. The two-sided Student’s t test was used. *, statistically significant. | | | | | | | | |

| Table S2a**.** The demographic, clinical and pathological characteristics of the enrolled populations | | | | | |
| --- | --- | --- | --- | --- | --- |
| **Characteristics** | **HPV-negative Pap-normal** ^a^ (n=17562) | **HPV-positive Pap-normal** (n=3596) | **HPV-negative Pap-abnormal** (n=850) | **HPV-positive Pap-abnormal** (n=2092) | **p value** ^b^ |
| **Age** (years) ^c^  16-19  20-29  30-39  40-49  50-59  60-69  70-79  80-89 | 203 (1)  3373 (19)  6351 (36)  4727 (27)  2396 (14)  475 (3)  35 (0.2)  2 (0.01) | 52 (2)  692 (19)  1312 (36)  966 (27)  489 (14)  79 (2)  6 (0.2)  0 (0) | 11 (1)  160 (19)  306 (36)  232 (27)  114 (14)  25 (3)  2 (0.2)  0 (0) | 21 (1)  379 (18)  808 (39)  560 (27)  275 (13)  41 (2)  8 (0.4)  0 (0) | 0.621 |
| **Gravidity**  0  1-2  3-4  ≥5 | 1986 (11)  7924 (45)  6396 (37)  1256 (7) | 395 (11)  1613 (45)  1320 (37)  268 (7) | 99 (12)  373 (44)  323 (38)  55 (6) | 227 (11)  938 (45)  782 (37)  145 (7) | 0.592 |
| **Parity**  0  1-2  ≥3 | 5982 (34)  11215 (64)  365 (2) | 1221 (34)  2297 (64)  78 (2) | 275 (32)  553 (65)  22 (3) | 721 (34)  1325 (63)  46 (3) | 0.236 |
| **Menopause**  No  Yes | 15756 (90)  1806 (10) | 3231 (90)  365 (10) | 754 (89)  96 (11) | 1891 (90)  201 (10) | 0.543 |
| **CS history** ^d^  No  Yes | 13203 (75)  4359 (25) | 2687 (75)  909 (25) | 657 (77)  193 (23) | 1608 (77)  484 (23) | 0.148 |
| **OC history** ^e^  Never  <3 months  ≥3 months | 15813 (90)  896 (5)  853 (5) | 3209 (89)  214 (6)  173 (5) | 747 (88)  58 (7)  45 (5) | 1862 (89)  124 (6)  106 (5) | 0.108 |
| **Pap test** ^f^  NILM  ASC-US  LSIL  ASC-H  HSIL | 17562 (100)  0 (0)  0 (0)  0 (0)  0 (0) | 3596 (100)  0 (0)  0 (0)  0 (0)  0 (0) | 0 (0)  521 (61)  312 (37)  9 (1)  8 (1) | 0 (0)  1206 (58)  768 (37)  22 (1)  96 (4) | - |
| **p16 ^INK4A^ quantification**  Normal  Abnormal | 16505 (94)  1057 (6) | 3419 (95)  177 (5) | 326 (38)  524 (62) | 698 (33)  1394 (67) | - |
| **Biopsy pathology**  Normal/Cervicitis  LSIL  HSIL | 17562 (100)  0 (0)  0 (0) | 3407 (95)  112 (3)  77 (2) | 742 (87)  66 (8)  42 (5) | 1446 (69)  287 (14)  359 (17) | - |
| a. For each population of women, data are presented as number (%). For percentages >0.5%, the data are given as an integer, while for those ≤0.5%, at least one significant digit is given in parentheses. The percentages were calculated based on the number of women enrolled in a study population.  b. The two-sided χ^2^ test was adopted to compare the differences in demographic, clinical and pathological characteristics among the study populations.  c. The differences in age-compositions between two study populations were additionally compared in pairs using the two-sided χ^2^ test in Table S2b.  d. “Cesarean section history” refers to at least one cesarean section experience in the life-time of the enrolled woman. CS, cesarean section.  e. “Oral contraceptive history” refers to the use of an oral contraceptive within 1 year before enrollment. Use of an oral contraceptive that had been ceased for >1 year before the enrollment was defined as “never used oral contraceptive”. OC, oral contraceptive.  f. Herein, pathological characteristics, namely, Pap test, p16^INK4A^ quantification and biopsy pathology results, were not compared for statistical significance. They were listed as an overview of their distributional patterns in each study population. | | | | | |

| **Table S2b.** Comparisons of the age-composition between study populations ^a^ | | | | |
| --- | --- | --- | --- | --- |
| **Study populations** | **HPV-negative Pap-normal**  (n=17562) | **HPV-positive Pap-normal** (n=3596) | **HPV-negative Pap-abnormal** (n=850) | **HPV-positive Pap-abnormal** (n=2092) |
| HPV-negative Pap-normal population | - | - | - | - |
| HPV-positive Pap-normal population | 0.576 | - | - | - |
| HPV-negative Pap-abnormal population | 0.999 | 0.911 | - | - |
| HPV-positive Pap-abnormal population | 0.099 | 0.270 | 0.569 | - |
| a. The differences in age-compositions between any two study populations were compared using the two-sided χ^2^ test. The p values are given and no statistical significance was found. | | | | |

| Table 3a**.** The age- and viral genotype-compositions of HPV-positive Pap-normal women (single-infections) and their corresponding p16^INK4A^ increments | | | | | | | | | | |
| --- | --- | --- | --- | --- | --- | --- | --- | --- | --- | --- |
| **HPV genotypes** | **16-19** ^a^ n (%) | **20-29** n (%) | **30-39** n (%) | **40-49** n (%) | **50-59** n (%) | **60-69** n (%) | **70-79** n (%) | **χ^2^** | **p value** ^b^ | **p16^INK4A^ increment** (%) ^c^ |
| HPV-6 (n=18) | 1 (6) | 2 (11) | 7 (39) | 4 (22) | 3 (17) | 1 (6) | 0 (0) | 4.496 | 0.721 | 0.2±0.4 |
| HPV-11 (n=9) | 0 (0) | 2 (22) | 4 (44) | 3 (33) | 0 (0) | 0 (0) | 0 (0) | 1.945 | 0.963 | 0.0±0.6 |
| HPV-16 (n=489) | 8 (2) | 100 (20) | 163 (33) | 132 (27) | 71 (15) | 13 (3) | 2 (0) | 3.725 | 0.811 | 1.6±0.1 |
| HPV-18 (n=172) | 3 (2) | 28 (16) | 61 (35) | 48 (28) | 27 (16) | 4 (2) | 1 (1) | 3.215 | 0.864 | 1.4±0.1 |
| HPV-31 (n=58) | 0 (0) | 11 (19) | 25 (43) | 18 (31) | 4 (7) | 0 (0) | 0 (0) | 5.428 | 0.608 | 1.4±0.2 |
| HPV-33 (n=54) | 0 (0) | 9 (17) | 22 (41) | 15 (28) | 7 (13) | 1 (2) | 0 (0) | 1.409 | 0.985 | 1.2±0.2 |
| HPV-35 (n=23) | 0 (0) | 3 (13) | 6 (26) | 7 (30) | 6 (26) | 1 (4) | 0 (0) | 4.353 | 0.738 | 1.1±0.6 |
| HPV-39 (n=84) | 1 (1) | 9 (11) | 33 (39) | 29 (35) | 11 (13) | 1 (1) | 0 (0) | 6.073 | 0.531 | 0.8±0.2 |
| HPV-42 (n=34) | 0 (0) | 7 (21) | 7 (21) | 13 (38) | 6 (18) | 1 (3) | 0 (0) | 4.796 | 0.685 | 1.4±0.2 |
| HPV-43 (n=14) | 0 (0) | 1 (7) | 8 (57) | 4 (29) | 0 (0) | 1 (7) | 0 (0) | 5.895 | 0.552 | 0.0±0.5 |
| HPV-44 (n=16) | 0 (0) | 2 (13) | 6 (38) | 4 (25) | 3 (19) | 1 (6) | 0 (0) | 1.670 | 0.976 | 0.6±0.4 |
| HPV-45 (n=23) | 0 (0) | 7 (30) | 6 (26) | 6 (26) | 4 (17) | 0 (0) | 0 (0) | 3.331 | 0.853 | 1.2±0.4 |
| HPV-51 (n=89) | 2 (2) | 11 (12) | 40 (45) | 23 (26) | 12 (13) | 1 (1) | 0 (0) | 6.007 | 0.539 | 1.1±0.1 |
| HPV-52 (n=288) | 5 (2) | 63 (22) | 108 (38) | 68 (24) | 37 (13) | 7 (2) | 0 (0) | 3.976 | 0.782 | 1.2±0.1 |
| HPV-53 (n=131) | 2 (2) | 30 (23) | 54 (41) | 28 (21) | 17 (13) | 0 (0) | 0 (0) | 7.344 | 0.394 | 0.8±0.2 |
| HPV-54 (n=1) | 0 (0) | 1 (100) | 0 (0) | 0 (0) | 0 (0) | 0 (0) | 0 (0) | 4.206 | 0.756 | 0.3±1.8 |
| HPV-55 (n=2) | 0 (0) | 1 (50) | 0 (0) | 1 (50) | 0 (0) | 0 (0) | 0 (0) | 2.461 | 0.930 | 0.6±1.3 |
| HPV-56 (n=100) | 1 (1) | 20 (20) | 37 (37) | 27 (27) | 12 (12) | 3 (3) | 0 (0) | 0.513 | 0.999 | 0.8±0.1 |
| HPV-58 (n=262) | 4 (2) | 47 (18) | 93 (35) | 75 (29) | 35 (13) | 7 (3) | 1 (0) | 1.305 | 0.988 | 1.3±0.1 |
| HPV-59 (n=74) | 0 (0) | 12 (16) | 27 (36) | 21 (28) | 12 (16) | 2 (3) | 0 (0) | 1.773 | 0.971 | 0.6±0.1 |
| HPV-60 (n=4) | 0 (0) | 1 (25) | 2 (50) | 1 (25) | 0 (0) | 0 (0) | 0 (0) | 0.996 | 0.995 | 0.3±0.9 |
| HPV-66 (n=64) | 0 (0) | 15 (23) | 20 (31) | 14 (22) | 12 (19) | 2 (3) | 1 (2) | 9.430 | 0.223 | 0.9±0.1 |
| HPV-67 (n=1) | 0 (0) | 1 (100) | 0 (0) | 0 (0) | 0 (0) | 0 (0) | 0 (0) | 4.206 | 0.756 | 0.8±1.8 |
| HPV-68 (n=103) | 2 (2) | 19 (18) | 44 (43) | 24 (23) | 11 (11) | 3 (3) | 0 (0) | 3.182 | 0.868 | 1.1±0.2 |
| HPV-73 (n=16) | 1 (6) | 1 (6) | 6 (38) | 5 (31) | 3 (19) | 0 (0) | 0 (0) | 5.864 | 0.556 | 0.9±0.4 |
| HPV-81 (n=59) | 1 (2) | 12 (20) | 19 (32) | 17 (29) | 8 (14) | 2 (3) | 0 (0) | 0.747 | 0.998 | 0.7±0.2 |
| HPV-82 (n=17) | 1 (6) | 5 (29) | 6 (35) | 5 (29) | 0 (0) | 0 (0) | 0 (0) | 7.048 | 0.424 | 1.2±0.2 |
| a. For each age group, the data are presented as number (%). The percentage data were calculated based on the number of women infected with a specific genotype of HPV for each line and given in the form of integers in the parentheses.  b. For each viral genotype, the age-related distributional characteristics of the infected women were compared seriatim with that of the HPV-negative women. The two-sided χ^2^ test was used for the analysis. Additionally, for the age groups, the viral genotype-related distributional characteristics of the infected women were compared with one another using the two-sided χ^2^ test. All the differences compared in this table were of no statistical significance; details of the statistical analyses can be found in Supplementary Table S3.  c. Data are presented as the mean ± SD. The p16^INK4A^ increment caused by a specific viral genotype was calculated based on the FCM data of those with single infections. The p16^INK4A^-positive ratios of the women infected with each specific genotype of HPV were seriatim compared with that of the women with no viral infections using the two-sided Student’s t test. The genotype-specific p16^INK4A^ increments and their related standard deviations (SD) were given. The statistical significances obtained by each comparison have been shown in Figure 2D. | | | | | | | | | | |

| **Table S3b.** The age- and viral genotype-compositions of HPV-positive Pap-normal women (single-infections) | | | | | | | | |
| --- | --- | --- | --- | --- | --- | --- | --- | --- |
| **HPV genotypes** | **16-19** ^a^ (n=32) | **20-29** (n=420) | **30-39** (n=804) | **40-49** (n=592) | **50-59** (n=301) | **60-69** (n=51) | **70-79** (n=5) | **Total** ^b^ (n=2205) |
| HPV-6 | 1 (1) | 2 (3) | 7 (0.5) | 4 (1) | 3 (1) | 1 (2) | 0 (0) | 18(1) |
| HPV-11 | 0 (0) | 2 (0.5) | 4 (0.5) | 3 (1) | 0 (0) | 0 (0) | 0 (0) | 9 (0.4) |
| HPV-16 | 8 (25) | 100 (24) | 163 (20) | 132 (22) | 71 (24) | 13 (25) | 2 (40) | 489 (22) |
| HPV-18 | 3 (9) | 28 (7) | 61 (8) | 48 (8) | 27 (9) | 4 (8) | 1 (20) | 172 (8) |
| HPV-31 | 0 (0) | 11 (3) | 25 (3) | 18 (3) | 4 (1) | 0 (0) | 0 (0) | 58 (3) |
| HPV-33 | 0 (0) | 9 (2) | 22 (3) | 15 (3) | 7 (2) | 1 (2) | 0 (0) | 54 (2) |
| HPV-35 | 0 (0) | 3 (1) | 6 (1) | 7 (1) | 6 (2) | 1 (2) | 0 (0) | 23 (1) |
| HPV-39 | 1 (3) | 9 (2) | 33 (4) | 29 (5) | 11 (4) | 1 (2) | 0 (0) | 84 (4) |
| HPV-42 | 0 (0) | 7 (2) | 7 (1) | 13 (2) | 6 (2) | 1 (2) | 0 (0) | 34 (2) |
| HPV-43 | 0 (0) | 1 (0.2) | 8 (1) | 4 (1) | 0 (0) | 1 (2) | 0 (0) | 14 (1) |
| HPV-44 | 0 (0) | 2 (0.5) | 6 (1) | 4 (1) | 3 (1) | 1 (2) | 0 (0) | 16 (1) |
| HPV-45 | 0 (0) | 7 (2) | 6 (1) | 6 (1) | 4 (1) | 0 (0) | 0 (0) | 23 (1) |
| HPV-51 | 2 (6) | 11 (3) | 40 (5) | 23 (4) | 12 (4) | 1 (2) | 0 (0) | 89 (4) |
| HPV-52 | 5 (16) | 63 (15) | 108 (13) | 68 (11) | 37 (12) | 7 (14) | 0 (0) | 288 (13) |
| HPV-53 | 2 (6) | 30 (7) | 54 (7) | 28 (5) | 17 (6) | 0 (0) | 0 (0) | 131 (6) |
| HPV-54 | 0 (0) | 1 (0.2) | 0 (0) | 0 (0) | 0 (0) | 0 (0) | 0 (0) | 1 (0.05) |
| HPV-55 | 0 (0) | 1 (0.2) | 0 (0) | 1 (0.2) | 0 (0) | 0 (0) | 0 (0) | 2 (0.1) |
| HPV-56 | 1 (3) | 20 (5) | 37 (5) | 27 (5) | 12 (4) | 3 (6) | 0 (0) | 100 (5) |
| HPV-58 | 4 (13) | 47 (11) | 93 (12) | 75 (13) | 35 (12) | 7 (14) | 1 (20) | 262 (12) |
| HPV-59 | 0 (0) | 12 (3) | 27 (3) | 21 (4) | 12 (4) | 2 (4) | 0 (0) | 74 (3) |
| HPV-60 | 0 (0) | 1 (0.2) | 2 (0.2) | 1 (0.2) | 0 (0) | 0 (0) | 0 (0) | 4 (0.2) |
| HPV-66 | 0 (0) | 15 (4) | 20 (2) | 14 (2) | 12 (4) | 2 (4) | 1 (20) | 64 (3) |
| HPV-67 | 0 (0) | 1 (0.2) | 0 (0) | 0 (0) | 0 (0) | 0 (0) | 0 (0) | 1 (0.05) |
| HPV-68 | 2 (6) | 19 (5) | 44 (5) | 24 (4) | 11 (4) | 3 (6) | 0 (0) | 103 (5) |
| HPV-73 | 1 (3) | 1 (0.2) | 6 (1) | 5 (1) | 3 (1) | 0 (0) | 0 (0) | 16 (1) |
| HPV-81 | 1 (3) | 12 (3) | 19 (2) | 17 (3) | 8 (3) | 2 (4) | 0 (0) | 59 (3) |
| HPV-82 | 1 (3) | 5 (1) | 6 (1) | 5 (1) | 0 (0) | 0 (0) | 0 (0) | 17 (1) |
| **χ^2^** | 14.721 | 18.005 | 10.903 | 11.828 | 19.815 | 12.848 | 9.618 | - |
| **p value** ^b. c^ | 0.962 | 0.875 | 0.996 | 0.992 | 0.800 | 0.985 | 0.999 | - |
| a. For each age-group, the data are presented as number (%). The percentage data were calculated based on the number of women in the age-group as indicated. For percentages >0.5%, the data are given as an integer, while for those ≤0.5%, at least one significant digit is given in parentheses.  b. The viral genotype-composition of each age-group was compared with that of a population of HPV-positive Pap-normal women with single-infections only. The two-sided χ^2^ test was used, while no statistical significance was found.  c. The differences in viral genotype-composition between two age-groups were additionally compared in pairs using the two-sided χ^2^ test in Table S3b. | | | | | | | | |

| **Table S3c.** Comparisons of the viral genotype-composition (single-infections) between age groups ^a^ | | | | | | | | |
| --- | --- | --- | --- | --- | --- | --- | --- | --- |
| **Age groups** | | **16-19** ^a^ (n=32) | **20-29** (n=420) | **30-39** (n=804) | **40-49** (n=592) | **50-59** (n=301) | **60-69** (n=51) | **70-79** (n=5) |
| 16-19 | χ^2^ p ^b^ | - | - | - | - | - | - | - |
| 20-29 | χ^2^ p | 17.428  0.896 | - | - | - | - | - | - |
| 30-39 | χ^2^ p | 12.123  0.969 | 26.384  0.442 | - | - | - | - | - |
| 40-49 | χ^2^ p | 13.210  0.963 | 22.174  0.679 | 14.983  0.921 | - | - | - | - |
| 50-59 | χ^2^ p | 18.603  0.548 | 22.338  0.670 | 23.453  0.435 | 15.028  0.920 | - | - | - |
| 60-69 | χ^2^ p | 13.721  0.800 | 15.668  0.944 | 13.392  0.943 | 12.060  0.979 | 13.757  0.843 | - | - |
| 70-79 | χ^2^ p | 10.048  0.690 | 8.447  1.000 | 11.026  0.983 | 10.621  0.991 | 7.062  0.994 | 5.885  0.989 | - |
| a. The differences in viral genotype-compositions between any two age-groups (of the HPV-positive Pap-normal population) were compared using the two-sided χ^2^ test. Only women with single-infections were involved in these comparisons. For each comparison, χ^2^ and p values are given; no statistical significance was found.  b. The “p” refers to p value. | | | | | | | | |

| **Table S3d.** Comparisons of the age-composition among women infected with various HPV genotypes (single-infections) ^a^ | | | | | | | | | | | | | | | | |
| --- | --- | --- | --- | --- | --- | --- | --- | --- | --- | --- | --- | --- | --- | --- | --- | --- |
| **HPV genotypes** | | **HPV-6**  (n=18) | **HPV-11**  (n=9) | **HPV-16**  (n=489) | **HPV-18**  (n=172) | **HPV-31**  (n=58) | **HPV-33**  (n=54) | **HPV-35**  (n=23) | **HPV-39**  (n=84) | **HPV-42**  (n=34) | **HPV-43**  (n=14) | **HPV-44**  (n=16) | **HPV-45**  (n=23) | **HPV-51**  (n=89) | **HPV-52**  (n=288) | **HPV-53**  (n=131) |
| HPV-6 | χ^2^ p ^b^ | - | - | - | - | - | - | - | - | - | - | - | - | - | - | - |
| HPV-11 | χ^2^ p | 3.331  0.649 | - | - | - | - | - | - | - | - | - | - | - | - | - | - |
| HPV-16 | χ^2^ p | 3.218  0.781 | 2.200  0.900 | - | - | - | - | - | - | - | - | - | - | - | - | - |
| HPV-18 | χ^2^ p | 2.406  0.879 | 2.295  0.891 | 1.619  0.951 | - | - | - | - | - | - | - | - | - | - | - | - |
| HPV-31 | χ^2^ p | 8.790  0.118 | 0.680  0.878 | 6.730  0.346 | 6.200  0.401 | - | - | - | - | - | - | - | - | - | - | - |
| HPV-33 | χ^2^ p | 4.242  0.515 | 1.579  0.813 | 2.450  0.874 | 1.817  0.936 | 2.343  0.673 | - | - | - | - | - | - | - | - | - | - |
| HPV-35 | χ^2^ p | 2.523  0.773 | 3.803  0.433 | 3.698  0.717 | 2.851  0.827 | 9.017  0.061 | 3.161  0.531 | - | - | - | - | - | - | - | - | - |
| HPV-39 | χ^2^ p | 3.715 0.591 | 2.361  0.797 | 6.929  0.327 | 3.484  0.746 | 4.536  0.475 | 2.122  0.832 | 4.195  0.522 | - | - | - | - | - | - | - | - |
| HPV-42 | χ^2^ p | 5.102 0.404 | 3.491  0.479 | 3.954  0.683 | 4.139  0.658 | 7.468  0.113 | 3.884  0.422 | 1.406  0.843 | 5.521  0.356 | - | - | - | - | - | - | - |
| HPV-43 | χ^2^ p | 3.962 0.555 | 1.808  0.613 | 6.785  0.341 | 5.976  0.426 | 6.562  0.161 | 4.239  0.375 | 6.287  0.179 | 5.274  0.383 | 8.468 0.076 | - | - | - | - | - | - |
| HPV-44 | χ^2^ p | 0.963 0.966 | 2.803  0.591 | 1.825  0.935 | 1.498  0.960 | 6.034  0.197 | 1.329  0.856 | 0.787  0.940 | 2.637 0.756 | 2.458 0.652 | 3.501 0.478 | - | - | - | - | - |
| HPV-45 | χ^2^ p | 4.860 0.433 | 2.539  0.468 | 2.610  0.856 | 3.915  0.688 | 4.194  0.241 | 3.088  0.543 | 3.077  0.545 | 6.737 0.241 | 2.008 0.734 | 8.499 0.075 | 3.166 0.530 | - | - | - | - |
| HPV-51 | χ^2^ p | 2.488 0.778 | 2.290  0.808 | 6.809  0.339 | 3.286  0.772 | 4.745  0.448 | 1.978  0.852 | 5.120  0.401 | 1.797 0.876 | 7.804 0.167 | 5.163 0.396 | 2.670 0.751 | 6.269 0.281 | - | - | - |
| HPV-52 | χ^2^ p | 3.029 0.696 | 1.988  0.851 | 3.473  0.747 | 4.922  0.554 | 5.515  0.356 | 2.040  0.844 | 5.355  0.374 | 7.798 0.168 | 6.191 0.288 | 6.007 0.306 | 2.155 0.827 | 2.797 0.731 | 4.900 0.428 | - | - |
| HPV-53 | χ^2^ p | 9.765 0.082 | 1.872  0.759 | 7.596  0.269 | 7.826  0.251 | 4.086  0.395 | 4.632  0.462 | 11.042  0.051 | 9.177 0.102 | 11.056 0.050 | 13.903 0.016 | 9.624 0.087 | 2.440 0.655 | 5.422 0.367 | 3.771 0.583 | - |
| HPV-54 | χ^2^ p | 5.630 0.344 | 2.593  0.274 | 3.859  0.696 | 4.994  0.545 | 3.984  0.263 | 4.583  0.333 | 5.217  0.266 | 7.589 0.180 | 3.474 0.482 | 6.964 0.073 | 4.958 0.292 | 2.087 0.555 | 6.573 0.254 | 3.528 0.619 | 3.283 0.512 |
| HPV-55 | χ^2^ p | 3.704 0.593 | 1.477  0.478 | 2.286  0.892 | 2.808  0.833 | 2.151  0.542 | 2.644  0.619 | 2.921  0.571 | 3.822 0.575 | 1.626 0.804 | 4.114 0.249 | 3.150 0.533 | 1.465 0.690 | 3.770 0.583 | 2.384 0.794 | 2.475 0.649 |
| HPV-56 | χ^2^ p | 3.217 0.667 | 1.702  0.889 | 1.375  0.967 | 2.091  0.911 | 3.842  0.572 | 1.087  0.955 | 4.071  0.539 | 4.156 0.527 | 4.101 0.535 | 4.796 0.441 | 1.499 0.913 | 2.908 0.714 | 3.756 0.585 | 0.871 0.972 | 5.325 0.378 |
| HPV-58 | χ^2^ p | 2.973 0.812 | 1.964  0.923 | 1.127  0.980 | 0.752  0.993 | 5.151  0.525 | 1.545  0.956 | 3.883  0.692 | 4.016 0.675 | 4.087 0.665 | 5.646 0.464 | 1.637 0.950 | 3.634 0.726 | 4.242 0.644 | 3.735 0.712 | 7.625 0.267 |
| HPV-59 | χ^2^ p | 4.947 0.422 | 2.078  0.721 | 2.453  0.874 | 1.779  0.939 | 4.478  0.345 | 0.474  0.976 | 1.773  0.778 | 3.065 0.690 | 2.876 0.579 | 4.870 0.301 | 0.722 0.949 | 3.038 0.551 | 3.641 0.602 | 3.266 0.659 | 7.291 0.200 |
| HPV-60 | χ^2^ p | 1.684 0.891 | 0.090  0.956 | 1.144  0.980 | 1.237  0.975 | 0.432  0.934 | 0.830  0.934 | 2.238  0.692 | 1.517 0.911 | 2.334 0.675 | 1.221 0.748 | 1.458 0.834 | 1.389 0.708 | 1.171 0.948 | 0.865 0.973 | 0.683 0.953 |
| HPV-66 | χ^2^ p | 5.376 0.497 | 2.977  0.704 | 4.034  0.672 | 4.226  0.646 | 8.396  0.136 | 3.456  0.630 | 2.421  0.788 | 9.774 0.135 | 3.702 0.593 | 7.181 0.207 | 1.513 0.912 | 1.704 0.888 | 8.959 0.176 | 7.708 0.260 | 9.218 0.162 |
| HPV-67 | χ^2^ p | 5.630 0.344 | 2.593  0.274 | 3.859  0.696 | 4.994  0.545 | 3.984  0.263 | 4.583  0.333 | 5.217  0.266 | 7.589 0.180 | 3.474 0.482 | 6.964 0.073 | 4.958 0.292 | 2.087 0.555 | 6.573 0.254 | 3.528 0.619 | 3.283 0.512 |
| HPV-68 | χ^2^ p | 2.142 0.829 | 1.812  0.874 | 4.148  0.657 | 3.461  0.749 | 4.243  0.515 | 1.748  0.883 | 5.890  0.317 | 5.070 0.407 | 7.196 0.206 | 4.053 0.542 | 1.921 0.860 | 4.509 0.479 | 2.380 0.794 | 1.337 0.931 | 4.802 0.441 |
| HPV-73 | χ^2^ p | 1.409 0.923 | 3.552  0.470 | 4.277  0.639 | 2.977  0.812 | 6.832  0.145 | 4.983  0.418 | 3.179  0.672 | 2.539 0.771 | 5.345 0.375 | 5.287 0.382 | 2.444 0.785 | 4.626 0.328 | 1.967 0.854 | 4.481 0.482 | 4.450 0.348 |
| HPV-81 | χ^2^ p | 2.099 0.835 | 2.092  0.836 | 0.464  0.998 | 1.234  0.975 | 5.215  0.390 | 1.956  0.855 | 2.652  0.753 | 3.883 0.566 | 2.464 0.782 | 5.533 0.354 | 1.345 0.930 | 2.310 0.805 | 3.967 0.554 | 1.163 0.948 | 6.391 0.270 |
| HPV-82 | χ^2^ p | 5.450 0.364 | 0.800  0.849 | 5.346  0.500 | 5.955  0.428 | 5.462  0.243 | 6.870  0.231 | 8.116  0.150 | 7.734 0.171 | 7.087 0.214 | 4.818 0.306 | 6.372 0.272 | 4.628 0.328 | 6.274 0.280 | 4.656 0.459 | 4.451 0.348 |
| All genotypes | χ^2^ p | 3.752 0.710 | 1.932  0.926 | 2.526  0.866 | 2.131  0.907 | 5.276  0.509 | 1.411  0.965 | 4.560  0.601 | 5.628 0.466 | 4.995 0.544 | 6.211 0.400 | 2.005 0.919 | 3.370 0.761 | 4.845 0.564 | 3.063 0.801 | 6.485 0.371 |
| (To be continued) | | | | | | | | | | | | | | | | |

| **Table S3d.** (continued) | | | | | | | | | | | | | | | | |
| --- | --- | --- | --- | --- | --- | --- | --- | --- | --- | --- | --- | --- | --- | --- | --- | --- |
| **HPV genotypes** | | **HPV-54**  (n=1) | **HPV-55**  (n=2) | **HPV-56**  (n=100) | **HPV-58**  (n=262) | **HPV-59**  (n=74) | **HPV-60**  (n=4) | **HPV-66**  (n=64) | **HPV-67**  (n=1) | **HPV-68**  (n=103) | **HPV-73**  (n=16) | **HPV-81**  (n=59) | **HPV-82**  (n=17) |  |  |  |
| HPV-6 | χ^2^ p ^b^ | - | - | - | - | - | - | - | - | - | - | - | - |  |  |  |
| HPV-11 | χ^2^ p | - | - | - | - | - | - | - | - | - | - | - | - |  |  |  |
| HPV-16 | χ^2^ p | - | - | - | - | - | - | - | - | - | - | - | - |  |  |  |
| HPV-18 | χ^2^ p | - | - | - | - | - | - | - | - | - | - | - | - |  |  |  |
| HPV-31 | χ^2^ p | - | - | - | - | - | - | - | - | - | - | - | - |  |  |  |
| HPV-33 | χ^2^ p | - | - | - | - | - | - | - | - | - | - | - | - |  |  |  |
| HPV-35 | χ^2^ p | - | - | - | - | - | - | - | - | - | - | - | - |  |  |  |
| HPV-39 | χ^2^ p | - | - | - | - | - | - | - | - | - | - | - | - |  |  |  |
| HPV-42 | χ^2^ p | - | - | - | - | - | - | - | - | - | - | - | - |  |  |  |
| HPV-43 | χ^2^ p | - | - | - | - | - | - | - | - | - | - | - | - |  |  |  |
| HPV-44 | χ^2^ p | - | - | - | - | - | - | - | - | - | - | - | - |  |  |  |
| HPV-45 | χ^2^ p | - | - | - | - | - | - | - | - | - | - | - | - |  |  |  |
| HPV-51 | χ^2^ p | - | - | - | - | - | - | - | - | - | - | - | - |  |  |  |
| HPV-52 | χ^2^ p | - | - | - | - | - | - | - | - | - | - | - | - |  |  |  |
| HPV-53 | χ^2^ p | - | - | - | - | - | - | - | - | - | - | - | - |  |  |  |
| HPV-54 | χ^2^ p | - | - | - | - | - | - | - | - | - | - | - | - |  |  |  |
| HPV-55 | χ^2^ p | 0.750 0.386 | - | - | - | - | - | - | - | - | - | - | - |  |  |  |
| HPV-56 | χ^2^ p | 3.848 0.572 | 2.295 0.807 | - | - | - | - | - | - | - | - | - | - |  |  |  |
| HPV-58 | χ^2^ p | 4.496 0.610 | 2.506 0.868 | 0.938 0.988 | - | - | - | - | - | - | - | - | - |  |  |  |
| HPV-59 | χ^2^ p | 4.834 0.305 | 2.722 0.605 | 1.665 0.893 | 1.864 0.932 | - | - | - | - | - | - | - | - |  |  |  |
| HPV-60 | χ^2^ p | 1.875 0.392 | 1.500 0.472 | 0.867 0.973 | 1.077 0.983 | 1.134 0.889 | - | - | - | - | - | - | - |  |  |  |
| HPV-66 | χ^2^ p | 3.110 0.683 | 2.333 0.801 | 4.417 0.620 | 5.165 0.523 | 3.067 0.690 | 1.367 0.928 | - | - | - | - | - | - |  |  |  |
| HPV-67 | χ^2^ p | NA ^c^ | 0.750 0.386 | 3.848 0.572 | 4.496 0.610 | 4.834 0.305 | 1.875 0.392 | 3.110 0.683 | - | - | - | - | - |  |  |  |
| HPV-68 | χ^2^ p | 4.241 0.515 | 2.778 0.734 | 1.140 0.951 | 2.718 0.843 | 3.435 0.633 | 0.762 0.979 | 6.598 0.360 | 4.241 0.515 | - | - | - | - |  |  |  |
| HPV-73 | χ^2^ p | 7.969 0.093 | 4.500 0.343 | 4.703 0.453 | 3.926 0.687 | 6.054 0.301 | 2.292 0.682 | 7.268 0.297 | 7.969 0.093 | 3.927 0.560 | - | - | - |  |  |  |
| HPV-81 | χ^2^ p | 3.677 0.597 | 2.110 0.834 | 0.521 0.991 | 0.625 0.996 | 1.945 0.857 | 1.160 0.949 | 3.251 0.777 | 3.677 0.597 | 1.892 0.864 | 3.326 0.650 | - | - |  |  |  |
| HPV-82 | χ^2^ p | 2.118 0.548 | 1.304 0.728 | 5.221 0.390 | 5.611 0.468 | 8.868 0.114 | 0.463 0.927 | 8.337 0.214 | 2.118 0.548 | 4.513 0.478 | 5.642 0.228 | 4.286 0.509 | - |  |  |  |
| All genotypes ^d^ | χ^2^ p | 4.242 0.644 | 2.484 0.870 | 0.801 0.992 | 0.869 0.990 | 1.997 0.920 | 0.986 0.986 | 8.140 0.228 | 4.242 0.644 | 2.719 0.843 | 4.662 0.588 | 0.862 0.990 | 5.978 0.426 |  |  |  |
| a. The differences in age-composition were pairwise compared among NILM women infected with various HPV genotypes and only those with single-infections were involved.  b. The two-sided χ^2^ test was used; and the “p” refers to p value. No statistically significant differences were found.  c. The comparison was not performed because there was only 1 case for each genotype (HPV-54 and HPV-67) compared. NA, not available.  d. The general age-compositions of the women with single infections were compared seriatim with that of women infected with a specific viral genotype. | | | | | | | | | | | | | | | | |

| **Table S3e.** Comparisons of the age-compositions between women with multiple- and single-infections ^a^ | | | | | | | | | | |
| --- | --- | --- | --- | --- | --- | --- | --- | --- | --- | --- |
| **HPV genotypes & multiplicity** | | **16-19** ^b^ n (%) | **20-29** n (%) | **30-39** n (%) | **40-49** n (%) | **50-59** n (%) | **60-69** n (%) | **70-79** n (%) | **χ^2^** | **p value** ^c^ |
| HPV-6 | M (n=31) S (n=18) | 1 (3) 1 (6) | 5 (16) 2 (11) | 12 (39) 7 (39) | 7 (23) 4 (22) | 5 (16) 3 (17) | 1 (3) 1 (6) | 0 (0) 0 (0) | 0.506 | 0.992 |
| HPV-11 | M (n=15) S (n=9) | 0 (0) 0 (0) | 4 (27) 2 (22) | 5 (33) 4 (44) | 2 (13) 3 (33) | 3 (20) 0 (0) | 1 (7) 0 (0) | 0 (0) 0 (0) | 3.710 | 0.447 |
| HPV-16 | M (n=719) S (n=489) | 8 (1) 8 (2) | 149 (21) 100 (20) | 272 (38) 163 (33) | 196 (27) 132 (27) | 80 (11) 71 (15) | 14 (2) 13 (3) | 0 (0) 2 (0.4) | 8.534 | 0.202 |
| HPV-18 | M (n=377) S (n=172) | 7 (2) 3 (2) | 59 (16) 28 (16) | 138 (37) 61 (35) | 105 (28) 48 (28) | 60 (16) 27 (16) | 8 (2) 4 (2) | 0 (0) 1 (1) | 2.298 | 0.890 |
| HPV-31 | M (n=71) S (n=58) | 1 (1) 0 (0) | 9 (13) 11 (19) | 31 (44) 25 (43) | 22 (31) 18 (31) | 6 (8) 4 (7) | 2 (3) 0 (0) | 0 (0) 0 (0) | 3.367 | 0.644 |
| HPV-33 | M (n=68) S (n=54) | 1 (1) 0 (0) | 11 (16) 9 (17) | 27 (40) 22 (41) | 22 (32) 15 (28) | 6 (9) 7 (13) | 1 (1) 1 (2) | 0 (0) 0 (0) | 1.525 | 0.910 |
| HPV-35 | M (n=65) S (n=23) | 2 (3) 0 (0) | 10 (15) 3 (13) | 18 (28) 6 (26) | 19 (29) 7 (30) | 15 (23) 6 (26) | 1 (2) 1 (4) | 0 (0) 0 (0) | 1.450 | 0.919 |
| HPV-39 | M (n=124) S (n=84) | 1 (1) 1 (1) | 16 (13) 9 (11) | 47 (38) 33 (39) | 44 (35) 29 (35) | 13 (10) 11 (13) | 3 (2) 1 (1) | 0 (0) 0 (0) | 1.004 | 0.962 |
| HPV-42 | M (n=54) S (n=34) | 2 (4) 0 (0) | 11 (20) 7 (21) | 12 (22) 7 (21) | 19 (35) 13 (38) | 9 (17) 6 (18) | 1 (2) 1 (3) | 0 (0) 0 (0) | 1.460 | 0.918 |
| HPV-43 | M (n=70) S (n=14) | 2 (3) 0 (0) | 5 (7) 1 (7) | 40 (57) 8 (57) | 18 (26) 4 (29) | 3 (4) 0 (0) | 2 (3) 1 (7) | 0 (0) 0 (0) | 1.636 | 0.897 |
| HPV-44 | M (n=20) S (n=16) | 1 (5) 0 (0) | 3 (15) 2 (13) | 7 (35) 6 (38) | 5 (25) 4 (25) | 3 (15) 3 (19) | 1 (5) 1 (6) | 0 (0) 0 (0) | 0.955 | 0.966 |
| HPV-45 | M (n=33) S (n=23) | 0 (0) 0 (0) | 9 (27) 7 (30) | 10 (30) 6 (26) | 8 (24) 6 (26) | 5 (15) 4 (17) | 1 (3) 0 (0) | 0 (0) 0 (0) | 0.889 | 0.926 |
| HPV-51 | M (n=133) S (n=89) | 1 (1) 2 (2) | 23 (17) 11 (12) | 57 (43) 40 (45) | 29 (22) 23 (26) | 21 (16) 12 (13) | 2 (2) 1 (1) | 0 (0) 0 (0) | 2.402 | 0.791 |
| HPV-52 | M (n=368) S (n=288) | 4 (1) 5 (2) | 91 (25) 63 (22) | 153 (42) 108 (38) | 76 (21) 68 (24) | 37 (10) 37 (13) | 7 (2) 7 (2) | 0 (0) 0 (0) | 3.704 | 0.593 |
| HPV-53 | M (n=226) S (n=131) | 3 (1) 2 (2) | 60 (27) 30 (23) | 86 (38) 54 (41) | 48 (21) 28 (21) | 25 (11) 17 (13) | 4 (2) 0 (0) | 0 (0) 0 (0) | 3.251 | 0.661 |
| HPV-54 | M (n=7) S (n=1) | 0 (0) 0 (0) | 1 (14) 1 (100) | 2 (29) 0 (0) | 2 (29) 0 (0) | 1 (14) 0 (0) | 1 (14) 0 (0) | 0 (0) 0 (0) | 3.429 | 0.489 |
| HPV-55 | M (n=6) S (n=2) | 0 (0) 0 (0) | 1 (17) 1 (50) | 2 (33) 0 (0) | 1 (17) 1 (50) | 1 (17) 0 (0) | 1 (17) 0 (0) | 0 (0) 0 (0) | 2.667 | 0.615 |
| HPV-56 | M (n=142) S (n=100) | 3 (2) 1 (1) | 29 (20) 20 (20) | 59 (42) 37 (37) | 32 (23) 27 (27) | 16 (11) 12 (12) | 2 (1) 3 (3) | 1 (1) 0 (0) | 3.787 | 0.706 |
| HPV-58 | M (n=256) S (n=262) | 3 (1) 4 (2) | 56 (22) 47 (18) | 103 (40) 93 (35) | 65 (25) 75 (29) | 26 (10) 35 (13) | 3 (1) 7 (3) | 0 (0) 1 (0) | 6.013 | 0.422 |
| HPV-59 | M (n=101) S (n=74) | 2 (2) 0 (0) | 20 (20) 12 (16) | 38 (38) 27 (36) | 26 (26) 21 (28) | 13 (13) 12 (16) | 2 (2) 2 (3) | 0 (0) 0 (0) | 2.323 | 0.803 |
| HPV-60 | M (n=4) S (n=4) | 0 (0) 0 (0) | 1 (25) 1 (25) | 1 (25) 2 (50) | 1 (25) 1 (25) | 0 (0) 0 (0) | 1 (25) 0 (0) | 0 (0) 0 (0) | 1.333 | 0.721 |
| HPV-66 | M (n=122) S (n=64) | 0 (0) 0 (0) | 33 (27) 15 (23) | 41 (34) 20 (31) | 24 (20) 14 (22) | 21 (17) 12 (19) | 2 (2) 2 (3) | 1 (1) 1 (2) | 1.085 | 0.955 |
| HPV-67 | M (n=7) S (n=1) | 0 (0) 0 (0) | 1 (14) 1 (100) | 2 (29) 0 (0) | 2 (29) 0 (0) | 1 (14) 0 (0) | 1 (14) 0 (0) | 0 (0) 0 (0) | 3.429 | 0.489 |
| HPV-68 | M (n=152) S (n=103) | 1 (1) 2 (2) | 26 (17) 19 (18) | 66 (43) 44 (43) | 37 (24) 24 (23) | 19 (13) 11 (11) | 2 (1) 3 (3) | 1 (1) 0 (0) | 2.607 | 0.856 |
| HPV-73 | M (n=26) S (n=16) | 0 (0) 1 (6) | 3 (12) 1 (6) | 11 (42) 6 (38) | 7 (27) 5 (31) | 4 (15) 3 (19) | 1 (4) 0 (0) | 0 (0) 0 (0) | 2.720 | 0.743 |
| HPV-81 | M (n=142) S (n=59) | 2 (1) 1 (2) | 31 (22) 12 (20) | 49 (35) 19 (32) | 39 (27) 17 (29) | 19 (13) 8 (14) | 2 (1) 2 (3) | 0 (0) 0 (0) | 0.982 | 0.964 |
| HPV-82 | M (n=18) S (n=17) | 0 (0) 1 (6) | 5 (28) 5 (29) | 7 (39) 6 (35) | 4 (22) 5 (29) | 1 (6) 0 (0) | 1 (6) 0 (0) | 0 (0) 0 (0) | 3.162 | 0.675 |
| a. The differences in age-compositions were pairwise compared between HPV-positive NILM women with multiple-infections and single-infections for each viral genotype. M, multiple-infection; S, single-infection.  b. Data are presented as number (%). The percentages were calculated based on the number of women with the indicated viral genotype and infection multiplicity (single vs. multiple). For percentages ≥0.5%, the data are given as an integer, while for those <0.5%, at least one significant digit is given in parentheses.  c. The two-sided χ^2^ test was used, and no statistical significance was found.  d. Together with Table 2, the tables (Tables S3a-d) indicated a homogeneous base (viral genotype-composition and age-composition) for comparisons of the age- or genotype-specific p16^INK4A^-positive ratios (increments) in Figure 2. | | | | | | | | | | |

| Table S4. Comparison of the age-compositions among HPV-negative women with Pap abnormalities ^a^ | | | | | | |
| --- | --- | --- | --- | --- | --- | --- |
| **Age groups** (years) | **ASC-US**  (n=521) ^b^ | **LSIL**  (n=312) | **ASC-H**  (n=9) | **HSIL**  (n=8) | **χ^2^** | **p value** |
| 16-19 | 5 (1) | 5 (2) | 1 (11) | 0 (0) | 11.032 | 0.893 |
| 20-29 | 101 (19) | 57 (18) | 1 (11) | 1 (13) |  |  |
| 30-39 | 189 (36) | 109 (35) | 4 (44) | 4 (50) |  |  |
| 40-49 | 139 (27) | 89 (29) | 2 (22) | 2 (25) |  |  |
| 50-59 | 70 (13) | 42 (13) | 1 (11) | 1 (13) |  |  |
| 60-69 | 15 (3) | 10 (3) | 0 (0) | 0 (0) |  |  |
| 70-79 | 2 (0.4) | 0 (0) | 0 (0) | 0 (0) |  |  |
| a. The differences in age-compositions among women enrolled with a variety of Pap abnormalities in the HPV-negative condition were compared using the two-sided χ^2^ test.  b. Data are presented as number (%). The percentages were calculated based on the number of women with the indicated Pap abnormality in a column. For percentages >0.5%, the data are given as an integer, while for those ≤0.5%, at least one significant digit is given in parentheses. | | | | | | |

| Table S5a**.** Comparison of the age-compositions among HPV-positive women with Pap abnormalities ^a^ | | | | | | |
| --- | --- | --- | --- | --- | --- | --- |
| **Age groups** | **ASC-US**  (n=1206) ^b^ | **LSIL**  (n=768) | **ASC-H**  (n=22) | **HSIL**  (n=96) | **χ^2^** | **p value** |
| 16-19 | 12 (2) | 9 (3) | 0 (0) | 0 (0) | 8.540 | 0.969 |
| 20-29 | 226 (43) | 133 (43) | 5 (56) | 15 (188) |  |  |
| 30-39 | 471 (90) | 289 (93) | 9 (100) | 39 (488) |  |  |
| 40-49 | 306 (59) | 221 (71) | 5 (56) | 28 (350) |  |  |
| 50-59 | 163 (31) | 98 (31) | 2 (22) | 12 (150) |  |  |
| 60-69 | 23 (4) | 16 (5) | 1 (11) | 1 (13) |  |  |
| 70-79 | 5 (1) | 2 (1) | 0 (0) | 1 (13) |  |  |
| a. The differences in age-compositions among women enrolled with a variety of Pap abnormalities in the HPV-positive condition were compared using the two-sided χ^2^ test.  b. Data are presented as number (%). The percentages were calculated based on the number of women with the indicated Pap abnormality in a column and are given as integers in parentheses. | | | | | | |

| **Table S5b.** Comparison of the viral genotype-compositions among HPV-positive women with Pap abnormalities ^a,c^ | | | | | | |
| --- | --- | --- | --- | --- | --- | --- |
| **HPV genotypes** | **ASC-US**  (n=1206) ^b^ | **LSIL**  (n=768) | **ASC-H**  (n=22) | **HSIL**  (n=96) | **χ^2^** | **p value** |
| HPV-6 | 18 (1) | 10 (1) | 0 (0) | 0 (0) | 57.405 | 0.961 |
| HPV-11 | 8 (0.4) | 8 (1) | 0 (0) | 0 (0) |  |  |
| HPV-16 | 423 (22) | 255 (21) | 10 (24) | 36 (25) |  |  |
| HPV-18 | 182 (10) | 114 (10) | 8 (20) | 19 (13) |  |  |
| HPV-31 | 43 (2) | 26 (2) | 0 (0) | 3 (2) |  |  |
| HPV-33 | 41 (2) | 24 (2) | 0 (0) | 3 (2) |  |  |
| HPV-35 | 32 (2) | 17 (1) | 0 (0) | 0 (0) |  |  |
| HPV-39 | 70 (4) | 44 (4) | 3 (7) | 4 (3) |  |  |
| HPV-42 | 33 (2) | 17 (1) | 0 (0) | 0 (0) |  |  |
| HPV-43 | 28 (1) | 17 (1) | 0 (0) | 1 (1) |  |  |
| HPV-44 | 12 (1) | 7 (1) | 0 (0) | 0 (0) |  |  |
| HPV-45 | 19 (1) | 11 (1) | 0 (0) | 0 (0) |  |  |
| HPV-51 | 73 (4) | 45 (4) | 1 (2) | 7 (5) |  |  |
| HPV-52 | 222 (12) | 138 (12) | 8 (20) | 18 (12) |  |  |
| HPV-53 | 120 (6) | 75 (6) | 1 (2) | 10 (7) |  |  |
| HPV-54 | 7 (0.4) | 7 (1) | 0 (0) | 0 (0) |  |  |
| HPV-55 | 6 (0.3) | 8 (1) | 0 (0) | 0 (0) |  |  |
| HPV-56 | 84 (4) | 52 (4) | 0 (0) | 7 (5) |  |  |
| HPV-58 | 179 (9) | 114 (10) | 4 (10) | 14 (10) |  |  |
| HPV-59 | 62 (3) | 36 (3) | 0 (0) | 5 (3) |  |  |
| HPV-60 | 3 (0.2) | 4 (0.3) | 0 (0) | 0 (0) |  |  |
| HPV-66 | 62 (3) | 38 (3) | 0 (0) | 7 (5) |  |  |
| HPV-67 | 3 (0.2) | 9 (1) | 0 (0) | 0 (0) |  |  |
| HPV-68 | 85 (4) | 55 (5) | 5 (12) | 6 (4) |  |  |
| HPV-73 | 12 (1) | 11 (1) | 0 (0) | 0 (0) |  |  |
| HPV-81 | 68 (4) | 42 (4) | 1 (2) | 5 (3) |  |  |
| HPV-82 | 11 (1) | 6 (1) | 0 (0) | 0 (0) |  |  |
| a. The differences in viral genotype-compositions among women enrolled with a variety of Pap abnormalities in the HPV-positive condition were compared using the two-sided χ^2^ test.  b. Data are presented as number (%). The percentages were calculated based on the number of women with the Pap abnormality indicated in a column. For percentages >0.5%, the data are given as an integer, while for those ≤0.5%, at least one significant digit is given in parentheses.  c. Tables S4 and S5a-b indicated a homogeneous base (age- and viral genotype-compositions) for the comparisons of TBS-categorized lesion-specific p16^INK4A^-positive ratios (increments) in Figure 3. | | | | | | |

| Table S6a**.** The age-dependent p16^INK4A^-positive ratios among HPV-negative women ^a^ | | | | | | | |
| --- | --- | --- | --- | --- | --- | --- | --- |
| **Age groups** | **NILM**  (n=17562) ^b^ | **ASC-US**  (n=521) | **LSIL**  (n=312) | **ASC-H**  (n=9) | **HSIL**  (n=8) | **F** | **p value** |
| 16-19 | 13.6%±1.8% | 21.2%±9.9% | 17.2%±3.7% | 16.7% | - | 21.917 | <0.001* |
| 20-29 | 13.7%±1.9% | 20.3%±5.7% | 17.4%±5.1% | 23.8% | 20.4% | 275.486 | <0.001* |
| 30-39 | 14.0%±1.7% | 19.5%±5.9% | 17.5%±4.8% | 20.0%±6.0% | 21.4%±8.2% | 415.393 | <0.001* |
| 40-49 | 14.1%±1.7% | 18.3%±5.9% | 18.3%±4.9% | 19.6%±3.8% | 21.6%±14.6% | 230.703 | <0.001* |
| 50-59 | 14.0%±1.8% | 18.3%±5.8% | 17.3%±5.9% | 22.4% | 22.2% | 98.471 | <0.001* |
| 60-69 | 13.1%±1.7% | 22.1%±5.8% | 17.0%±4.1% | - | - | 168.294 | <0.001* |
| 70-79 | 13.1%±1.6% | 23.5%±4.3% | - | - | - | 69.645 | <0.001* |
| 80-89 | 13.6%±4.1% | - | - | - | - | - | - |
| All ages | 13.9%±1.8% | 19.3%±5.9% | 17.7%±5.0% | 20.2%±4.4% | 21.4%±7.7% | 1070.231 | <0.001* |
| a. The differences in the p16^INK4A^-positive ratios were compared among HPV-negative women with various Pap test results (Pap-normal and Pap-abnormal) for each age-group. ANOVA was used; and F and p values are given in the table. *, statistically significant.  b. Data are presented as the mean ± SD. For age-groups with 1 case, only a mean is given. | | | | | | | |

| **Table S6b.** The age-dependent p16^INK4A^-positive ratios among HPV-positive women ^a^ | | | | | | | |
| --- | --- | --- | --- | --- | --- | --- | --- |
| **Age groups** | **NILM**  (n=3596) ^b^ | **ASC-US**  (n=1206) | **LSIL**  (n=768) | **ASC-H**  (n=22) | **HSIL**  (n=96) | **F** | **p value** |
| 16-19 | 14.5%±1.3% | 21.6%±7.1% | 17.3%±5.2% | - | - | 21.206 | <0.001* |
| 20-29 | 14.5%±1.8% | 19.9%±5.1% | 17.6%±5.2% | 18.9%±12.7% | 17.6%±7.7% | 109.709 | <0.001* |
| 30-39 | 15.2%±1.6% | 19.6%±5.2% | 18.1%±5.2% | 21.3%±9.4% | 20.2%±8.0% | 158.365 | <0.001* |
| 40-49 | 15.4%±1.4% | 19.8%±5.4% | 18.2%±5.1% | 20.4%±9.2% | 19.9%±8.2% | 108.751 | <0.001* |
| 50-59 | 15.2%±1.6% | 19.3%±5.5% | 17.8%±5.1% | 12.5%±13.8% | 18.5%±8.7% | 45.975 | <0.001* |
| 60-69 | 14.4%±1.6% | 19.0%±2.9% | 15.7%±5.0% | 27.1% | 26.0% | 22.802 | <0.001* |
| 70-79 | 14.0%±0.7% | 21.9%±5.1% | 15.7%±0.7% | - | 7.1% | 8.486 | 0.004* |
| All ages | 15.1%±1.6% | 19.7%±5.2% | 18.0%±5.2% | 20.0%±9.9% | 19.4%±8.1% | 436.099 | <0.001* |
| a. The differences in the p16^INK4A^-positive ratios were compared among HPV-positive women with various Pap test results (Pap-normal and Pap-abnormal) for each age-group. ANOVA was used; and F and p values were given in the table. *, statistically significant.  b. Data are presented as the mean ± SD. For age-groups with 1 case, only a mean is given. | | | | | | | |

| **Table S6c.** The numbers (rates) of p16^INK4A^-abnormal cases among HPV-negative women ^a^ | | | | | | | |
| --- | --- | --- | --- | --- | --- | --- | --- |
| **Age groups** | **NILM**  (n=17562) ^b^ | **ASC-US**  (n=521) | **LSIL**  (n=312) | **ASC-H**  (n=9) | **HSIL**  (n=8) | **F** | **p value** |
| 16-19 | 10 (4) | 3 (60) | 3 (60) | 0 (0) | - | 41.869 | <0.001* |
| 20-29 | 243 (7) | 65 (64) | 39 (68) | 1 (100) | 1 (100) | 601.484 | <0.001* |
| 30-39 | 326 (5) | 111 (58) | 77 (70) | 2 (50) | 3 (75) | 1378.676 | <0.001* |
| 40-49 | 290 (6) | 69 (49) | 58 (65) | 1 (50) | 2 (100) | 732.305 | <0.001* |
| 50-59 | 170 (7) | 34 (48) | 30 (71) | 1 (100) | 1 (100) | 350.009 | <0.001* |
| 60-69 | 15 (3) | 14 (93) | 7 (70) | - | - | 237.194 | <0.001* |
| 70-79 | 3 (8) | 2 (100) | - | - | - | -** | 0.015* |
| 80-89 | 0 (0) | - | - | - | - | - | - |
| All ages | 1057 (6) | 298 (57) | 214 (68) | 5 (55) | 7 (87) | 3235.817 | <0.001* |
| a. The numbers and rates (%) of p16^INK4A^-abnormal cases were compared among HPV-negative women with various Pap test results (Pap-normal and Pap-abnormal) for each age-group. The normal range of p16^INK4A^ expression in the cervix was defined as age-adjusted p16^INK4A^-positive ratio ≥11.2% and <18.6% in FCM. The two-sided χ^2^ test was used; and F and p values are given in the table. *, statistically significant. **, Fisher’s exact test.  b. Data are presented as number (%). | | | | | | | |

| **Table S6d.** The numbers (rates) of p16^INK4A^-abnormal cases among HPV-positive women ^a^ | | | | | | | |
| --- | --- | --- | --- | --- | --- | --- | --- |
| **Age groups** | **NILM**  (n=3596) ^b^ | **ASC-US**  (n=1206) | **LSIL**  (n=768) | **ASC-H**  (n=22) | **HSIL**  (n=96) | **F** | **p value** |
| 16-19 | 1 (1) | 11 (91) | 8 (88) | - | - | 58.992 | <0.001* |
| 20-29 | 52 (7) | 178 (78) | 82 (61) | 3 (60) | 10 (66) | 494.406 | <0.001* |
| 30-39 | 57 (4) | 315 (66) | 178 (61) | 5 (55) | 34 (87) | 954.718 | <0.001* |
| 40-49 | 36 (3) | 202 (66) | 126 (57) | 4 (80) | 26 (92) | 689.926 | <0.001* |
| 50-59 | 18 (3) | 105 (64) | 54 (55) | 2 (100) | 8 (66) | 322.513 | <0.001* |
| 60-69 | 13 (16) | 21 (91) | 12 (75) | 1 (100) | 1 (100) | 54.638 | <0.001* |
| 70-79 | 0 (0) | 5 (100) | 2 (100) | - | 1 (100) | 14.000 | 0.003* |
| All ages | 177 (4) | 837 (69) | 462 (60) | 15 (68) | 80 (83) | 2553.769 | <0.001* |
| a. The numbers and rates (%) of p16^INK4A^-abnormal cases were compared among HPV-positive women with various Pap test results (Pap-normal and Pap-abnormal) for each age-group. The normal range of p16^INK4A^ expression in the cervix was defined as age-adjusted p16^INK4A^-positive ratio ≥11.9% and <18.3% in FCM. The two-sided χ^2^ test was used; and F and p values are given in the table. *, statistically significant.  b. Data are presented as number (%). | | | | | | | |

| **Table S6e.** The relationships between age, type of transformation zone and p16^INK4A^ expression in HPV-positive HSILs ^a^ | | | | |
| --- | --- | --- | --- | --- |
| **Characteristics** ^b^ | **HSILs with p16^INK4A^ insufficient expression**  (p16^INK4A^-positive ratio <17.2%) | **HSILs with p16^INK4A^ overexpression**  (p16^INK4A^-positive ratio ≥17.2%) | **χ^2^** | **p value** |
| **Age** (years)  20-29  30-39  40-49  50-59  60-69  70-79 | 4 (17)  7 (30)  6 (26)  5 (22)  0 (0)  1 (4) | 11 (15)  32 (44)  22 (30)  7 (10)  1 (1)  0 (0) | 6.486 | 0.262 |
| **Type of trans-formation zone**  1  2  3 | 8 (35)  6 (26)  9 (39) | 30 (41)  26 (36)  17 (23) | 2.273 | 0.321 |
| a. The possible influences of age and type of transformation zone on the expression of p16^INK4A^ were analyzed using the two-sided χ^2^ test in the HPV-positive HSIL cases. We observed no significant relationships between age, type of transformation zone and p16^INK4A^ expression in the cases analyzed, demonstrating that the insufficient expression (i.e., lower-than-normal) of p16^INK4A^ should not result from the inadequate sampling at the type 3 transformation zone of cervix in the corresponding HSIL cases. Or, inadequate sampling at Type 3 transformation zone is not a contributing factor to the lower-than-normal expression of p16^INK4A^ observed in a part of HPV-positive HSILs (i.e., p16^INK4A^-positive ratio <17.2%).  b. Data are presented as number (%). | | | | |

| **Table S6f.** The relationship between age and type of transformation zone in HPV-positive HSILs ^a^ | | | | | |
| --- | --- | --- | --- | --- | --- |
| **Age groups** ^b^ | **Types of transformation zone** | | | **χ^2^** | **p value** |
|  | 1 | 2 | 3 |  |  |
| 20-29  30-39  40-49  50-59  60-69  70-79 | 7 (18)  20 (53)  9 (24)  2 (5)  0 (0)  0 (0) | 6 (19)  14 (44)  10 (31)  2 (6)  0 (0)  0 (0) | 2 (8)  5 (19)  9 (35)  8 (31)  1 (4)  1 (4) | 21.415 | 0.018 * |
| a. The relationship between age and type of transformation zone was analyzed using the two-sided χ^2^ test in HPV-positive HSIL cases. It could be found that Type 1 transformation zone was more frequently detected in younger women and Type 3 transformation zone more often appeared in the elderly women, while the observed distributional differences were of statistical significance (*).  b. Data are presented as number (%). | | | | | |

| Table S7a**.** The detailed performance of p16^INK4A^ FCM and other known HSIL+-triaging strategies in the 24100 women enrolled ^a^ | | | |
| --- | --- | --- | --- |
| **HSIL+-triaging strategies** | **Lesions <HSIL** (n=23622) ^b^ | **Biopsy-confirmed HSIL** (n=478) | **p value** ^c^ |
| **The single-cutoff-ratio strategy**  Normal (p16^INK4A^-positive ratio <17.2%)  Abnormal (p16^INK4A^-positive ratio ≥17.2%) | 20674 (88)  2948 (12) | 67 (14)  411 (86) | <0.001* |
| **The double-cutoff-ratio strategy**  Normal (p16^INK4A^-positive ratio ≥11.4% and <18.0%)  Abnormal (p16^INK4A^-positive ratio <11.4% or ≥18.0%) | 20450 (87)  3172 (13) | 46 (10)  432 (90) | <0.001* |
| **The HPV DNA-combined double-cutoff-ratio strategy**  Normal  Abnormal  (Normal range: p16^INK4A^-positive ratio ≥11.2% and <18.6% in the HPV-negative condition; ≥11.9% and <18.3% in the HPV-positive condition) | 20911 (89)  2711 (11) | 50 (10)  428 (90) | <0.001* |
| **HPV DNA test strategy 1**  HPV DNA negative  Any HPV DNA positive | 18370 (78)  5252 (22) | 42 (9)  436 (91) | <0.001* |
| **HPV DNA test strategy 2**  HR HPV DNA negative  HR HPV DNA positive | 18634 (79)  4988 (21) | 47 (10)  431 (90) | <0.001* |
| **HPV DNA test strategy 3**  HPV-16 and HPV-18 DNA negative  HPV-16/18 DNA positive | 21334 (90)  2288 (10) | 227 (47)  251 (53) | <0.001* |
| **Pap test strategy 1**  NILM  ≥ASC-US | 21081 (89)  2541 (11) | 77 (16)  401 (84) | <0.001* |
| **Pap test strategy 2**  NILM/ASC-US  ≥LSIL | 22697 (96)  925 (4) | 188 (39)  290 (61) | <0.001* |
| **Pap test strategy 3**  NILM/ASC-US/LSIL  ≥ASC-H | 23590 (100)  32 (0.1) | 375 (78)  103 (22) | <0.001* |
| **Pap test strategy 3**  NILM/ASC-US/LSIL/ASC-H  ≥HSIL | 23602 (100)  20 (0.08) | 394 (82)  84 (18) | <0.001* |
| **HPV DNA and Pap cotest strategy**  Normal  Abnormal  (Normal criteria: i. HPV-16 and HPV-18 negative; and ii, other HR HPV-negative and Pap ≤LSIL or iii, other HR HPV-positive but Pap =NILM) | 20407 (86)  3215 (14) | 67 (14)  411 (86) | <0.001* |
| a. This is a dataset supplementary to Table 3. Note that the term “p16^INK4A^-positive ratio” refers to the age-adjusted p16^INK4A^-positive ratio in this table as well as in Table 3.  b. Data are presented as number (%). The percentages were calculated based on the number of women with the indicated histological lesions in a column. For percentages >0.5%, the data are given as an integer, while for those ≤0.5%, at least one significant digit is given for each one in the parentheses.  c. The two-sided χ^2^ test was used. *, statistically significant. | | | |

| **Table S7b.** The detailed performance of p16^INK4A^ FCM and other known HSIL+-triaging strategies in the HPV-negative women enrolled ^a^ | | | |
| --- | --- | --- | --- |
| **HSIL+-triaging strategies** | **Lesions <HSIL** (n=18370) ^b^ | **Biopsy-confirmed HSIL** (n=42) | **p value** ^c^ |
| **The single-cutoff-ratio strategy**  Normal (p16^INK4A^-positive ratio <17.2%)  Abnormal (p16^INK4A^-positive ratio ≥17.2%) | 16932 (92)  1438 (8) | 11 (26)  31 (74) | <0.001* |
| **The double-cutoff-ratio strategy**  Normal (p16^INK4A^-positive ratio ≥11.4% and <18.0%)  Abnormal (p16^INK4A^-positive ratio <11.4% or ≥18.0%) | 16468 (90)  1902 (10) | 4 (10)  38 (90) | <0.001* |
| **The HPV DNA-combined double-cutoff-ratio strategy**  Normal  Abnormal  (Normal range: p16^INK4A^-positive ratio ≥11.2% and <18.6%) | 16847 (92)  1523 (18) | 6 (14)  36 (86) | <0.001* |
| **Pap test strategy 1**  NILM  ≥ASC-US | 17562 (96)  808 (4) | 0 (0)  42 (100) | <0.001* |
| **Pap test strategy 2**  NILM/ASC-US  ≥LSIL | 18070 (98)  300 (2) | 13 (31)  29 (69) | <0.001* |
| **Pap test strategy 3**  NILM/ASC-US/LSIL  ≥ASC-H | 18361 (100)  9 (0.04) | 34 (81)  8 (19) | <0.001* |
| **Pap test strategy 3**  NILM/ASC-US/LSIL/ASC-H  ≥HSIL | 18367 (100)  3 (0.01) | 37 (88)  5 (12) | <0.001* |
| a. This is a dataset supplementary to Table S7a. Note that the term “p16^INK4A^-positive ratio” refers to the age-adjusted p16^INK4A^-positive ratio in this table as in Table 7a.  b. Data are presented as number (%). The percentages were calculated based on the number of women with the indicated histological lesions in a column. For percentages >0.5%, the data are given as an integer, while for those ≤0.5%, at least one significant digit is given for each one in the parentheses.  c. The two-sided χ^2^ test was used. *, statistically significant. | | | |

| **Table S7c.** The detailed performance of p16^INK4A^ FCM and other known HSIL+-triaging strategies in the HPV-positive women enrolled ^a^ | | | |
| --- | --- | --- | --- |
| **HSIL+-triaging strategies** | **Lesions <HSIL** (n=5252) ^b^ | **Biopsy-confirmed HSIL** (n=436) | **p value** ^c^ |
| **The single-cutoff-ratio strategy**  Normal (p16^INK4A^-positive ratio <17.2%)  Abnormal (p16^INK4A^-positive ratio ≥17.2%) | 3742 (71)  1510 (29) | 56 (13)  380 (87) | <0.001* |
| **The double-cutoff-ratio strategy**  Normal (p16^INK4A^-positive ratio ≥11.4% and <18.0%)  Abnormal (p16^INK4A^-positive ratio <11.4% or ≥18.0%) | 3982 (76)  1270 (24) | 42 (10)  394 (90) | <0.001* |
| **The HPV DNA-combined double-cutoff-ratio strategy**  Normal  Abnormal  (Normal range: p16^INK4A^-positive ratio ≥11.9% and <18.3%) | 4064 (77)  1188 (23) | 44 (10)  392 (90) | <0.001* |
| **HPV DNA test strategy 2**  HR HPV DNA negative  HR HPV DNA positive | 264 (5)  4988 (95) | 5 (1)  431 (99) | <0.001* |
| **HPV DNA test strategy 3**  HPV-16 and HPV-18 DNA negative  HPV-16/18 DNA positive | 2964 (56)  2288 (44) | 185 (42)  251 (58) | <0.001* |
| **Pap test strategy 1**  NILM  ≥ASC-US | 3519 (67)  1733 (33) | 77 (18)  359 (82) | <0.001* |
| **Pap test strategy 2**  NILM/ASC-US  ≥LSIL | 4627 (88)  625 (12) | 157 (40)  261 (60) | <0.001* |
| **Pap test strategy 3**  NILM/ASC-US/LSIL  ≥ASC-H | 5229 (100)  23 (0.4) | 341 (78)  95 (22) | <0.001* |
| **Pap test strategy 3**  NILM/ASC-US/LSIL/ASC-H  ≥HSIL | 5235 (100)  17 (0.3) | 357 (82)  79 (18) | <0.001* |
| **HPV DNA and Pap cotest strategy**  Normal  Abnormal  (Abnormal criteria: i. HPV-16 and HPV-18 positive; or ii, other HR HPV-positive and Pap ≥ASC-US) | 2046 (39)  3206 (61) | 33 (8)  403 (92) | <0.001* |
| a. This is a dataset supplementary to Table S7a. Note that the term “p16^INK4A^-positive ratio” refers to the age-adjusted p16^INK4A^-positive ratio in this table as in Table 7a.  b. Data are presented as number (%). The percentages were calculated based on the number of women with the indicated histological lesions in a column. For percentages >0.5%, the data are given as an integer, while for those ≤0.5%, at least one significant digit is given for each one in the parentheses.  c. The two-sided χ^2^ test was used. *, statistically significant. | | | |

| **Table S7d.** The detailed information on the HSIL+-triaging performances of viral genotypes and infection multiplicity as using an HPV-genotyping strategy ^a^ | | | | | |
| --- | --- | --- | --- | --- | --- |
| **HPV genotypes** | **Lesions <HSIL** (n=23622) ^b^ | **Biopsy-confirmed HSIL** (n=478) | **Infection Multiplicity** ^c^ | **Immediate risk of HSIL+** ^d^ | **p value** ^e^ |
| **HPV-6** |  |  | A (n=77) | 3.9% |  |
| Positive | 74 (0.3) | 3 (1) | S (n=27) | 0% | 0.095 |
| Negative | 23548 (100) | 475 (99) | M (n=50) | 6.0% |  |
| **HPV-11** |  |  | A (n=40) | 7.5% |  |
| Positive | 37 (0.2) | 3 (1) | S (n=15) | 6.7% | 0.042* |
| Negative | 23585 (100) | 475 (99) | M (n=25) | 8% |  |
| **HPV-16** |  |  | A (n=1932) | 10.0% |  |
| Positive | 1738 (7) | 194 (41) | S (n=774) | 13.0% | <0.001* |
| Negative | 21884 (93) | 284 (59) | M (n=1158) | 8.0% |  |
| **HPV-18** |  |  | A (n=872) | 9.5% |  |
| Positive | 789 (3) | 83 (17) | S (n=276) | 10.1% | <0.001* |
| Negative | 22833 (97) | 395 (83) | M (n=596) | 9.2% |  |
| **HPV-31** |  |  | A (n=201) | 7.0% |  |
| Positive | 187 (1) | 14 (3) | S (n=90) | 6.7% | <0.001* |
| Negative | 23435 (99) | 464 (97) | M (n=111) | 7.2% |  |
| **HPV-33** |  |  | A (n=190) | 5.3% |  |
| Positive | 180 (1) | 10 (2) | S (n=84) | 4.8% | 0.005* |
| Negative | 23442 (99) | 468 (98) | M (n=106) | 5.7% |  |
| **HPV-35** |  |  | A (n=137) | 5.8% |  |
| Positive | 129 (0.5) | 8 (2) | S (n=35) | 5.7% | 0.005* |
| Negative | 23493 (100) | 470 (98) | M (n=102) | 5.9% |  |
| **HPV-39** |  |  | A (n=329) | 4.0% |  |
| Positive | 316 (1) | 13 (3) | S (n=132) | 1.5% | 0.001* |
| Negative | 23306 (99) | 465 (97) | M (n=197) | 5.6% |  |
| **HPV-42** |  |  | A (n=138) | 5.8% |  |
| Positive | 130 (1) | 8 (2) | S (n=51) | 2% | <0.001* |
| Negative | 23492 (99) | 470 (98) | M (n=87) | 8% |  |
| **HPV-43** |  |  | A (n=130) | 8.5% |  |
| Positive | 119 (0.5) | 11 (2) | S (n=22) | 0% | <0.001* |
| Negative | 23503 (100) | 467 (98) | M (n=108) | 10.2% |  |
| **HPV-44** |  |  | A (n=55) | 1.8% |  |
| Positive | 54 (0.2) | 1 (0.2) | S (n=24) | 0% | 0.694 |
| Negative | 23568 (100) | 477 (100) | M (n=31) | 3.2% |  |
| **HPV-45** |  |  | A (n=86) | 8.1% |  |
| Positive | 79 (0.3) | 7 (1) | S (n=35) | 8.6% | <0.001* |
| Negative | 23543 (100) | 471 (99) | M (n=51) | 7.8% |  |
| **HPV-51** |  |  | A (n=348) | 6.0% |  |
| Positive | 327 (1) | 21 (4) | S (n=138) | 5.1% | <0.001* |
| Negative | 23295 (99) | 457 (96) | M (n=210) | 6.7% |  |
| **HPV-52** |  |  | A (n=1042) | 7.8% |  |
| Positive | 961 (4) | 81 (17) | S (n=455) | 6.4% |  |
| Negative | 22661 (96) | 397 (83) | M (n=587) | 8.9% |  |
| **HPV-53** |  |  | A (n=563) | 5.3% |  |
| Positive | 533 (2) | 30 (6) | S (n=206) | 3.4% | <0.001* |
| Negative | 23089 (98) | 448 (94) | M (n=357) | 6.4% |  |
| **HPV-54** |  |  | A (n=22) | 9.1% |  |
| Positive | 20 (0.1) | 2 (0.4) | S (n=4) | 0% | 0.02* |
| Negative | 23602 (100) | 476 (100) | M (n=18) | 11.1% |  |
| **HPV-55** |  |  | A (n=22) | 9.1% |  |
| Positive | 20 (0.1) | 2 (0.4) | S (n=6) | 0% | 0.01* |
| Negative | 23602 (100) | 476 (100) | M (n=16) | 12.5% |  |
| **HPV-56** |  |  | A (n=385) | 8.3% |  |
| Positive | 353 (2) | 32 (7) | S (n=159) | 5.0% | <0.001* |
| Negative | 23269 (98) | 446 (93) | M (n=226) | 10.6% |  |
| **HPV-58** |  |  | A (n=829) | 8.1% |  |
| Positive | 762 (3) | 67 (14) | S (n=416) | 7.0% | <0.001* |
| Negative | 22860 (97) | 411 (86) | M (n=413) | 9.2% |  |
| **HPV-59** |  |  | A (n=278) | 7.9% |  |
| Positive | 256 (1) | 22 (5) | S (n=116) | 6.0% | <0.001* |
| Negative | 23366 (99) | 456 (95) | M (n=162) | 9.3% |  |
| **HPV-60** |  |  | A (n=15) | 6.7% |  |
| Positive | 14 (0.06) | 1 (0.2) | S (n=6) | 0% | 0.137 |
| Negative | 23608 (100) | 477 (100) | M (n=9) | 11.1% |  |
| **HPV-66** |  |  | A (n=293) | 7.8% |  |
| Positive | 270 (1) | 23 (5) | S (n=100) | 5.0% | <0.001* |
| Negative | 23352 (99) | 455 (95) | M (n=193) | 9.3% |  |
| **HPV-67** |  |  | A (n=20) | 10.0% |  |
| Positive | 18 (0.1) | 2 (0.4) | S (n=4) | 0% | 0.01* |
| Negative | 23604 (100) | 476 (100) | M (n=16) | 12.5% |  |
| **HPV-68** |  |  | A (n=406) | 9.1% |  |
| Positive | 369 (2) | 37 (8) | S (n=162) | 7.4% | <0.001* |
| Negative | 23253 (98) | 441 (92) | M (n=244) | 10.2% |  |
| **HPV-73** |  |  | A (n=65) | 7.7% |  |
| Positive | 60 (0.3) | 5 (1) | S (n=24) | 8.3% | 0.004* |
| Negative | 23562 (100) | 473 (99) | M (n=41) | 7.3% |  |
| **HPV-81** |  |  | A (n=317) | 7.3% |  |
| Positive | 294 (1) | 23 (5) | S (n=94) | 3.2% | <0.001* |
| Negative | 23328 (99) | 455 (95) | M (n=223) | 9.0% |  |
| **HPV-82** |  |  | A (n=52) | 3.8% |  |
| Positive | 50 (0.2) | 2 (0.4) | S (n=26) | 7.7% | 0.087 |
| Negative | 23572 (100) | 476 (100) | M (n=26) | 0% |  |
| a. This table presented additional information to Table S7a, which was focused on the HSIL+-triaging performance of an HPV genotyping-based DNA test. The influence of multiple-infections on the carcinogenetic ability of a particular genotype of HPV was evaluated in this table, which could be reflected by the altered immediate risk of HSIL+ in the multiple-infection condition.  b. Data are presented as number (%). The percentages were calculated based on the number of women with the indicated histological lesions in a column. For percentages >0.5%, the data are given as an integer, while for those ≤0.5%, at least one significant digit is given for each one in the parentheses.  c. The viral infections were divided into two forms, single-infection and multiple-infection, and their respective and general carcinogenetic abilities were evaluated. A, any infection (including the single- and multiple-infection forms); S, single-infection; M, multiple-infection.  d. The immediate risk of HSIL+ is equivalent to the positive predictive value (PPV) calculated on the basis of viral infection with a particular genotype and multiplicity. The difference of immediate risk of HSIL+ between single-infection and multiple-infections of a particular genotype of HPV should be correlated to the alteration in the p16^INK4A^-positive ratios after the coinfection of this genotype with another genotype(s) of HPV (Figure 2G).  e. The difference in immediate risks (i.e., PPVs) of histological HSIL+ under absent-, single- and multiple-infection conditions was compared using the two-sided χ^2^ test for each genotype of HPV. *, statistically significant. | | | | | |

| Table S8a**.** Detailed outcomes of HPV-positive women with single-infections in Cohort 1 (classified based on p16^INK4A^ quantification) ^a^ | | | | | |
| --- | --- | --- | --- | --- | --- |
| **HPV genotypes** | **p16^INK4A^** **quantification** | **1-year risk of  HSIL+** | **p value** ^b^ | **2-year risk of  HSIL+** | **p value** |
| **HPV-6** | O (n=18) | 0% |  | 0% |  |
|  | N ^c^ (n=18) | 0% | NA | 0% | NA |
|  | A (n=0) | NA |  | NA |  |
| **HPV-11** | O (n=9) | 0% |  | 0% |  |
|  | N (n=9) | 0% | NA | 0% | NA |
|  | A (n=0) | NA |  | NA |  |
| **HPV-16** | O (n=443) | 2.9% |  | 4.1% |  |
|  | N (n=430) | 2.8% | 0.302 | 4.0% | 0.501 |
|  | A (n=13) | 7.7% |  | 7.7% |  |
| **HPV-18** | O (n=159) | 5.7% |  | 6.3% |  |
|  | N (n=149) | 5.4% | 0.540 | 5.4% | 0.065 |
|  | A (n=10) | 10.0% |  | 20.0% |  |
| **HPV-31** | O (n=57) | 1.8% |  | 1.8% |  |
|  | N (n=56) | 1.8% | 0.893 | 1.8% | 0.893 |
|  | A (n=1) | 0% |  | 0% |  |
| **HPV-33** | O (n=54) | 1.9% |  | 3.7% |  |
|  | N (n=53) | 1.9% | 0.890 | 3.8% | 0.843 |
|  | A (n=1) | 0% |  | 0% |  |
| **HPV-35** | O (n=18) | 0% |  | 5.6% |  |
|  | N (n=17) | 0% | NA | 0% | <0.001* |
|  | A (n=1) | 0% |  | 100% |  |
| **HPV-39** | O (n=82) | 1.2% |  | 1.2% |  |
|  | N (n=81) | 1.2% | 0.911 | 1.2% | 0.911 |
|  | A (n=1) | 0% |  | 0% |  |
| **HPV-42** | O (n=33) | 0% |  | 3.0% |  |
|  | N (n=32) | 0% | NA | 3.1% | 0.858 |
|  | A (n=1) | 0% |  | 0% |  |
| **HPV-43** | O (n=43) | 7.7% |  | 7.7% |  |
|  | N (n=42) | 8.3% | 0.764 | 8.3% | 0.764 |
|  | A (n=1) | 0% |  | 0% |  |
| **HPV-44** | O (n=16) | 0% |  | 0% |  |
|  | N (n=16) | 0% | NA | 0% | NA |
|  | A (n=0) | NA |  | NA |  |
| **HPV-45** | O (n=23) | 4.3% |  | 4.3% |  |
|  | N (n=23) | 4.3% | NA | 4.3% | NA |
|  | A (n=0) | NA |  | NA |  |
| **HPV-51** | O (n=86) | 1.2% |  | 1.2% |  |
|  | N (n=86) | 1.2% | NA | 1.2% | NA |
|  | A (n=0) | NA |  | NA |  |
| **HPV-52** | O (n=273) | 1.5% |  | 3.3% |  |
|  | N (n=269) | 1.5% | 0.806 | 3.3% | 0.710 |
|  | A (n=4) | 0% |  | 0% |  |
| **HPV-53** | O (n=129) | 0.8% |  | 2.3% |  |
|  | N (n=127) | 0.8% | 0.900 | 2.4% | 0.826 |
|  | A (n=2) | 0% |  | 0% |  |
| **HPV-54** | O (n=1) | 0% |  | 0% |  |
|  | N (n=1) | 0% | NA | 0% | NA |
|  | A (n=0) | NA |  | NA |  |
| **HPV-55** | O (n=2) | 0% |  | 0% |  |
|  | N (n=2) | 0% | NA | 0% | NA |
|  | A (n=0) | NA |  | NA |  |
| **HPV-56** | O (n=99) | 4% |  | 5.1% |  |
|  | N (n=9) | 4% | NA | 5.1% | NA |
|  | A (n=0) | NA |  | NA |  |
| **HPV-58** | O (n=247) | 2% |  | 3.6% |  |
|  | N (n=243) | 2.1% | 0.772 | 3.7% | 0.695 |
|  | A (n=4) | 0% |  | 0% |  |
| **HPV-59** | O (n=74) | 0% |  | 0% |  |
|  | N (n=73) | 0% | NA | 0% | NA |
|  | A (n=1) | 0% |  | 0% |  |
| **HPV-60** | O (n=4) | 0% |  | 0% |  |
|  | N (n=4) | 0% | NA | 0% | NA |
|  | A (n=0) | NA |  | NA |  |
| **HPV-66** | O (n=64) | 1.6% |  | 1.6% |  |
|  | N (n=64) | 1.6% | NA | 1.6% | NA |
|  | A (n=0) | NA |  | NA |  |
| **HPV-67** | O (n=1) | 0% |  | 0% |  |
|  | N (n=1) | 0% | NA | 0% | NA |
|  | A (n=0) | NA |  | NA |  |
| **HPV-68** | O (n=96) | 3.1% |  | 5.2% |  |
|  | N (n=94) | 3.2% | 0.797 | 4.3% | 0.004* |
|  | A (n=2) | 0% |  | 50% |  |
| **HPV-73** | O (n=15) | 0% |  | 0% |  |
|  | N (n=14) | 0% | NA | 0% | NA |
|  | A (n=1) | 0% |  | 0% |  |
| **HPV-81** | O (n=56) | 0% |  | 0% |  |
|  | N (n=55) | 0% | NA | 0% | NA |
|  | A (n=1) | 0% |  | 0% |  |
| **HPV-82** | O (n=17) | 0% |  | 0% |  |
|  | N (n=17) | 0% | NA | 0% | NA |
|  | A (n=0) | NA |  | NA |  |
| a. The 1-year and 2-year HSIL+ outcomes of women infected with various genotypes of HPV in Cohort 1 are presented in this table in three subsituations, namely, overall (O), p16^INK4A^-normal (N) and p16^INK4A^-abnormal (A). Only women with single-infections were included.  b. Differences in the 1-year and 2-year risks (i.e., PPVs) of histological HSIL+ of the related women (in Cohort 1) were seriatim compared between p16^INK4A^-normal and p16^INK4A^-abnormal cases as per their viral infection genotypes. The two-sided χ^2^ test was used. NA, not available. *, statistically significant.  c. Definition of a normal p16^INK4A^ test: positive ratio ≥11.2% and <18.6% in the HPV-negative condition, or ≥11.9% and <18.3% in the HPV-positive condition. | | | | | |

| **Table S8b.** Detailed outcomes of HPV-positive women with multiple-infections in Cohort 1 (classified based on p16^INK4A^ quantification) ^a^ | | | | | |
| --- | --- | --- | --- | --- | --- |
| **HPV genotypes** | **p16^INK4A^** **quantification** | **1-year risk of  HSIL+** | **p value** ^b^ | **2-year risk of  HSIL+** | **p value** |
| **HPV-6** | O (n=30) | 0% |  | 0% |  |
|  | N ^c^ (n=30) | 0% | NA | 0% | NA |
|  | A (n=0) | NA |  | NA |  |
| **HPV-11** | O (n=14) | 0% |  | 0% |  |
|  | N (n=14) | 0% | NA | 0% | NA |
|  | A (n=0) | NA |  | NA |  |
| **HPV-16** | O (n=681) | 3.4% |  | 4.6% |  |
|  | N (n=667) | 3.1% | 0.022* | 4.3% | 0.077 |
|  | A (n=14) | 14.3% |  | 14.3% |  |
| **HPV-18** | O (n=355) | 1.1% |  | 2.3% |  |
|  | N (n=350) | 0.9% | <0.001* | 2% | 0.007* |
|  | A (n=5) | 20.0% |  | 20.0% |  |
| **HPV-31** | O (n=68) | 1.5% |  | 4.4% |  |
|  | N (n=66) | 1.5% | 0.861 | 3.0% | 0.001* |
|  | A (n=2) | 0% |  | 50.0% |  |
| **HPV-33** | O (n=63) | 3.2% |  | 3.2% |  |
|  | N (n=63) | 3.2% | NA | 3.2% | NA |
|  | A (n=0) | NA |  | NA |  |
| **HPV-35** | O (n=63) | 1.6% |  | 3.2% |  |
|  | N (n=61) | 0% | <0.001* | 1.6% | <0.001* |
|  | A (n=2) | 50% |  | 50% |  |
| **HPV-39** | O (n=120) | 0.8% |  | 3.3% |  |
|  | N (n=116) | 0.9% | 0.852 | 3.4% | 0.706 |
|  | A (n=4) | 0% |  | 0% |  |
| **HPV-42** | O (n=52) | 7.7% |  | 7.7% |  |
|  | N (n=52) | 7.7% | NA | 7.7% | NA |
|  | A (n=0) | NA |  | NA |  |
| **HPV-43** | O (n=63) | 0% |  | 0% |  |
|  | N (n=63) | 0% | NA | 0% | NA |
|  | A (n=0) | NA |  | NA |  |
| **HPV-44** | O (n=20) | 5.0% |  | 10.0% |  |
|  | N (n=20) | 5.0% | NA | 10.0% | NA |
|  | A (n=0) | NA |  | NA |  |
| **HPV-45** | O (n=32) | 3.1% |  | 6.3% |  |
|  | N (n=32) | 3.1% | NA | 6.3% | NA |
|  | A (n=0) | NA |  | NA |  |
| **HPV-51** | O (n=126) | 0.8% |  | 0.8% |  |
|  | N (n=123) | 0% | <0.001* | 0% | <0.001* |
|  | A (n=3) | 33.3% |  | 33.3% |  |
| **HPV-52** | O (n=348) | 2.6% |  | 3.7% |  |
|  | N (n=341) | 2.3% | 0.049* | 3.5% | 0.137 |
|  | A (n=7) | 14.3% |  | 14.3% |  |
| **HPV-53** | O (n=215) | 1.9% |  | 2.3% |  |
|  | N (n=207) | 1.4% | 0.023* | 1.4% | <0.001* |
|  | A (n=8) | 12.5% |  | 25.0% |  |
| **HPV-54** | O (n=6) | 0% |  | 0% |  |
|  | N (n=6) | 0% | NA | 0% | NA |
|  | A (n=0) | NA |  | NA |  |
| **HPV-55** | O (n=6) | 0% |  | 0% |  |
|  | N (n=6) | 0% | NA | 0% | NA |
|  | A (n=0) | NA |  | NA |  |
| **HPV-56** | O (n=130) | 0.8% |  | 0.8% |  |
|  | N (n=128) | 0% | <0.001* | 0% | <0.001* |
|  | A (n=2) | 50.0% |  | 50.0% |  |
| **HPV-58** | O (n=248) | 2.4% |  | 2.8% |  |
|  | N (n=246) | 2.4% | 0.823 | 2.8% | 0.809 |
|  | A (n=2) | 0% |  | 0% |  |
| **HPV-59** | O (n=92) | 1.1% |  | 1.1% |  |
|  | N (n=89) | 0% | <0.001* | 0% | <0.001* |
|  | A (n=3) | 33.3% |  | 33.3% |  |
| **HPV-60** | O (n=4) | 0% |  | 0% |  |
|  | N (n=4) | 0% | NA | 0% | NA |
|  | A (n=0) | NA |  | NA |  |
| **HPV-66** | O (n=115) | 3.5% |  | 4.3% |  |
|  | N (n=113) | 3.5% | NA | 4.4% | NA |
|  | A (n=2) | NA |  | NA |  |
| **HPV-67** | O (n=6) | 0% |  | 0% |  |
|  | N (n=6) | 0% | NA | 0% | NA |
|  | A (n=0) | NA |  | NA |  |
| **HPV-68** | O (n=142) | 2.1% |  | 2.1% |  |
|  | N (n=137) | 1.5% | 0.005* | 1.5% | 0.005* |
|  | A (n=5) | 20.0% |  | 20.0% |  |
| **HPV-73** | O (n=22) | 0% |  | 0% |  |
|  | N (n=22) | 0% | NA | 0% | NA |
|  | A (n=0) | NA |  | NA |  |
| **HPV-81** | O (n=137) | 2.2% |  | 4.4% |  |
|  | N (n=133) | 2.3% | 0.761 | 4.5% | 0.664 |
|  | A (n=4) | 0% |  | 0% |  |
| **HPV-82** | O (n=18) | 0% |  | 0% |  |
|  | N (n=17) | 0% | NA | 0% | NA |
|  | A (n=1) | 0% |  | 0% |  |
| a. The 1-year and 2-year HSIL+ outcomes of women infected with various genotypes of HPV in Cohort 1 are presented in this table in three subsituations, namely overall (O), p16^INK4A^-normal (N) and p16^INK4A^-abnormal (A). Only women with multiple-infections were included.  b. Differences in the 1-year and 2-year risks (i.e., PPVs) of histological HSIL+ of the related women (in Cohort 1) were seriatim compared between p16^INK4A^-normal and p16^INK4A^-abnormal cases as per their viral infection genotypes. The two-sided χ^2^ test was used. NA, not available. *, statistically significant.  c. Definition of a normal p16^INK4A^ test: positive ratio ≥11.2% and <18.6% in the HPV-negative condition, or ≥11.9% and <18.3% in the HPV-positive condition. | | | | | |

| **Table S8c.** Detailed outcomes of HPV-positive women with single- vs. multiple-infections in Cohort 1 (p16^INK4A^ as a cofounding factor) ^a, c^ | | | | | |
| --- | --- | --- | --- | --- | --- |
| **HPV genotypes** | **Infection Multiplicity** ^c^ | **1-year risk of  HSIL+** | **p value** ^b^ | **2-year risk of  HSIL+** | **p value** |
| **HPV-6** | A (n=48) | 2.1% |  | 4.2% |  |
|  | S (n=18) | 0% | 0.747 | 0% | 0.424 |
|  | M (n=30) | 3.3% |  | 6.7% |  |
| **HPV-11** | A (n=23) | 4.3% |  | 4.3% |  |
|  | S (n=9) | 0% | 0.408 | 0% | 0.614 |
|  | M (n=14) | 7.1% |  | 7.1% |  |
| **HPV-16** | A (n=1124) | 3.2% |  | 4.4% |  |
|  | S (n=443) | 3.0% | 0.018* | 4.0% | 0.036* |
|  | M (n=618) | 3.4% |  | 4.6% |  |
| **HPV-18** | A (n=514) | 2.5% |  | 3.5% |  |
|  | S (n=159) | 6.0% | 0.005* | 6.0% | 0.055 |
|  | M (n=355) | 1.1% |  | 2.3% |  |
| **HPV-31** | A (n=125) | 1.6% |  | 3.2% |  |
|  | S (n=57) | 2.0% | 0.892 | 2.0% | 0.706 |
|  | M (n=68) | 1.5% |  | 4.4% |  |
| **HPV-33** | A (n=117) | 2.6% |  | 3.4% |  |
|  | S (n=54) | 2.0% | 0.856 | 4.0% | 0.982 |
|  | M (n=63) | 3.2% |  | 3.2% |  |
| **HPV-35** | A (n=81) | 1.2% |  | 3.7% |  |
|  | S (n=18) | 0% | 0.769 | 6.0% | 0.859 |
|  | M (n=63) | 1.6% |  | 3.2% |  |
| **HPV-39** | A (n=202) | 1.0% |  | 2.5% |  |
|  | S (n=82) | 1.0% | 0.473 | 1.0% | 0.575 |
|  | M (n=120) | 0.8% |  | 3.3% |  |
| **HPV-42** | A (n=85) | 4.7% |  | 5.9% |  |
|  | S (n=33) | 0% | 0.018* | 3.0% | 0.192 |
|  | M (n=52) | 7.7% |  | 7.7% |  |
| **HPV-43** | A (n=76) | 1.3% |  | 1.3% |  |
|  | S (n=13) | 8.0% | 0.197 | 8.0% | 0.228 |
|  | M (n=63) | 0.0% |  | 0.0% |  |
| **HPV-44** | A (n=36) | 2.8% |  | 5.6% |  |
|  | S (n=16) | 0% | 0.580 | 0% | 0.180 |
|  | M (n=20) | 5.0% |  | 10.0% |  |
| **HPV-45** | A (n=55) | 3.6% |  | 5.5% |  |
|  | S (n=23) | 4.0% | 0.731 | 4.0% | 0.604 |
|  | M (n=32) | 3.1% |  | 6.3% |  |
| **HPV-51** | A (n=212) | 0.9% |  | 0.9% |  |
|  | S (n=86) | 1.0% | 0.429 | 1.0% | 0.145 |
|  | M (n=126) | 0.8% |  | 0.8% |  |
| **HPV-52** | A (n=621) | 2.1% |  | 3.5% |  |
|  | S (n=273) | 2.0% | 0.627 | 3.0% | 0.866 |
|  | M (n=348) | 2.6% |  | 3.7% |  |
| **HPV-53** | A (n=344) | 1.5% |  | 2.3% |  |
|  | S (n=129) | 1.0% | 0.488 | 2.0% | 0.590 |
|  | M (n=215) | 1.9% |  | 2.3% |  |
| **HPV-54** | A (n=7) | 0% |  | 0% |  |
|  | S (n=1) | 0% | 0.924 | 0% | 0.889 |
|  | M (n=6) | 0% |  | 0% |  |
| **HPV-55** | A (n=8) | 0% |  | 0% |  |
|  | S (n=2) | 0% | 0.914 | 0% | 0.874 |
|  | M (n=6) | 0% |  | 0% |  |
| **HPV-56** | A (n=229) | 2.2% |  | 2.6% |  |
|  | S (n=99) | 4.0% | 0.247 | 5.0% | 0.167 |
|  | M (n=130) | 0.8% |  | 0.8% |  |
| **HPV-58** | A (n=495) | 2.2% |  | 3.2% |  |
|  | S (n=247) | 2.0% | 0.956 | 4.0% | 0.875 |
|  | M (n=248) | 2.4% |  | 2.8% |  |
| **HPV-59** | A (n=166) | 0.6% |  | 0.6% |  |
|  | S (n=74) | 0% | 0.317 | 0% | 0.131 |
|  | M (n=92) | 1.1% |  | 1.1% |  |
| **HPV-60** | A (n=8) | 0% |  | 0% |  |
|  | S (n=4) | 0% | 0.914 | 0% | 0.874 |
|  | M (n=4) | 0% |  | 0% |  |
| **HPV-66** | A (n=179) | 2.8% |  | 3.4% |  |
|  | S (n=64) | 2.0% | 0.604 | 2.0% | 0.601 |
|  | M (n=115) | 3.5% |  | 4.3% |  |
| **HPV-67** | A (n=7) | 0% |  | 0% |  |
|  | S (n=1) | 0% | 0.924 | 0% | 0.889 |
|  | M (n=6) | 0% |  | 0% |  |
| **HPV-68** | A (n=238) | 2.5% |  | 3.4% |  |
|  | S (n=96) | 3% | 0.821 | 5% | 0.417 |
|  | M (n=142) | 2.1% |  | 2.1% |  |
| **HPV-73** | A (n=37) | 0% |  | 0% |  |
|  | S (n=15) | 0% | 0.656 | 0% | 0.533 |
|  | M (n=22) | 0% |  | 0% |  |
| **HPV-81** | A (n=193) | 1.6% |  | 3.1% |  |
|  | S (n=56) | 0% | 0.526 | 0% | 0.296 |
|  | M (n=137) | 2.2% |  | 4.4% |  |
| **HPV-82** | A (n=35) | 0% |  | 0% |  |
|  | S (n=17) | 0% | 0.672 | 0% | 0.551 |
|  | M (n=18) | 0% |  | 0% |  |
| a. The 1-year and 2-year HSIL+ outcomes of women (in Cohort 1) with single-infections and multiple-infections were given as per the viral genotype indicated. A, any infection (including the single- and multiple-infection forms); S, single-infection; M, multiple-infection.  b. The difference in outcomes among absent-, single- and multiple-infection cases was compared using the two-sided χ^2^ test for each viral genotype. *, statistically significant.  c. The difference in HSIL+ outcomes (i.e., 1-year or 2-year risks) between single-infections and multiple-infections of a particular genotype of HPV could be correlated to the degree of p16^INK4A^-positive ratio alteration after its coinfection with another genotype(s) of HPV (Figure 2G). | | | | | |

| **Table S8d.** Detailed outcomes of women with Pap abnormalities in Cohort 2 (classified based on p16^INK4A^ quantification) ^a^ | | | | | |  |
| --- | --- | --- | --- | --- | --- | --- |
| **Initial Pap abnormalities** | **p16^INK4A^** **quantification** ^b^ | **1-year risk of  HSIL+** | **p value** ^c^ | **2-year risk of  HSIL+** | **p value** |  |
| **In the HPV-positive subsituation** | | | | | |  |
| ASC-US | Normal (n=216) | 0% | 0.119 | 0.5% | 0.430 | |
|  | Abnormal (n=485) | 1.1% |  | 1.1% |  |  |
| LSIL | Normal (n=86) | 1.2% | 0.977 | 1.2% | 0.977 | |
|  | Abnormal (n=166) | 1.2% |  | 1.2% |  |  |
| ASC-H | Normal (n=3) | 0% | NA | 0% | 0.046* | |
|  | Abnormal (n=1) | 0% |  | 100% |  |  |
| HSIL | Normal (n=0) | NA | NA | NA | NA | |
|  | Abnormal (n=1) | 100% |  | 100% |  |  |
| **In the HPV-negative subsituation** | | | | | |  |
| ASC-US | Normal (n=303) | 2.3% | <0.001* | 3.6% | <0.001* | |
|  | Abnormal (n=676) | 11.8% |  | 16% |  |  |
| LSIL | Normal (n=218) | 1.4% | <0.001* | 3.2% | <0.001* | |
|  | Abnormal (n=246) | 10.2% |  | 14.2% |  |  |
| ASC-H | Normal (n=2) | 0% | 0.083 | 0% | 0.083 | |
|  | Abnormal (n=1) | 100% |  | 100% |  |  |
| HSIL | Normal (n=0) | NA | NA | NA | NA | |
|  | Abnormal (n=0) | NA |  | NA |  |  |
| a. The influence of initial HPV DNA and Pap test results on the HSIL+ outcomes of Pap-abnormal biopsy-negative women in Cohort 2 are presented in detail in this table.  b. Differences in the 1-year and 2-year risks (i.e., PPVs) of histological HSIL+ of the related women (in Cohort 2) were seriatim compared between p16^INK4A^-normal and p16^INK4A^-abnormal cases as per their initial HPV DNA and Pap test results.  c. The two-sided χ^2^ test was used. NA, not available. *, statistically significant. | | | | | |  |

| **Table S8e.** Detailed outcomes of women with biopsy-confirmed LSIL in Cohort 3 (classified based on p16^INK4A^ quantification) ^a^ | | | | | |  |
| --- | --- | --- | --- | --- | --- | --- |
| **Initial Pap**  **test results** | **p16^INK4A^** **quantification** ^b^ | **1-year risk of  HSIL+** | **p value** ^c^ | **2-year risk of  HSIL+** | **p value** |  |
| **In the HPV-positive subsituation** | | | | | |  |
| NILM | Normal (n=0) | 0% | NA | 0% | NA | |
|  | Abnormal (n=0) | 0% |  | 0% |  |  |
| ASC-US | Normal (n=6) | 0% | NA | 0% | NA | |
|  | Abnormal (n=17) | 0% |  | 0% |  |  |
| LSIL | Normal (n=9) | 0% | NA | 0% | 0.579 | |
|  | Abnormal (n=30) | 0% |  | 3.3% |  |  |
| ASC-H | Normal (n=1) | 0% | NA | 0% | NA | |
|  | Abnormal (n=1) | 0% |  | 0% |  |  |
| HSIL | Normal (n=1) | 0% | NA | 0% | NA | |
|  | Abnormal (n=1) | 0% |  | 0% |  |  |
| **In the HPV-negative subsituation** | | | | | |  |
| NILM | Normal (n=80) | 0% | 0.006* | 1.3% | 0.036* | |
|  | Abnormal (n=32) | 9.4% |  | 9.4% |  |  |
| ASC-US | Normal (n=59) | 5.1% | 0.013* | 5.1% | <0.001* | |
|  | Abnormal (n=70) | 20.0% |  | 32.9% |  |  |
| LSIL | Normal (n=61) | 0% | <0.001* | 3.3% | <0.001* | |
|  | Abnormal (n=77) | 23.4% |  | 31.2% |  |  |
| ASC-H | Normal (n=1) | 0% | 0.386 | 0% | 0.386 | |
|  | Abnormal (n=2) | 50.0% |  | 50.0% |  |  |
| HSIL | Normal (n=10) | 0% | 0.006* | 20.0% | 0.115 | |
|  | Abnormal (n=7) | 57.1% |  | 57.1% |  |  |
| a. The influences of initial HPV DNA and Pap test results on the HSIL+ outcomes of biopsy-confirmed LSIL women in Cohort 3 have are presented in details in this table.  b. Differences in the 1-year and 2-year risks (i.e., PPVs) of histological HSIL+ of the related women (in Cohort 3) were seriatim compared between p16^INK4A^-normal and p16^INK4A^-abnormal cases as per their initial HPV DNA and Pap test results.  c. The two-sided χ^2^ test was used. NA, not available. *, statistically significant. | | | | | |  |
